# Plasma proteome profiling identified biomarkers for the differential diagnosis and molecular staging of neurodegenerative dementias

**DOI:** 10.1038/s43587-026-01162-7

**Published:** 2026-07-20

**Authors:** Giovanni Bellomo, Lisa Vermunt, Sjors in ‘t Veld, James D. Doecke, Yanaika S. Hok-A-Hin, Kateřina Veverová, Isabel M. Houtkamp, Daniel Alcolea, Steffen Halbgebauer, Carlos Quesada, Niklas Mattsson, Minerva Martinez-Castillo, María José López-Martínez, Alberto Rábano, Lynn Boonkamp, Christopher Fowler, Juan Fortea, Lorenzo Gaetani, Andrea Toja, Yolande Pijnenburg, Afina Lemstra, Wiesje M. van der Flier, Pascual Sánchez-Juan, Jakub Hort, Markus Otto, Sarah Anderl-Staub, Anne Sieben, Bart de Viel, Sebastiaan Engelborghs, Oskar Hansson, Colin Masters, Alberto Lleó, Lucilla Parnetti, Charlotte E. Teunissen, Marta del Campo

**Affiliations:** 1 Section of Neurology, Laboratory of Clinical Neurochemistry, Department of Medicine and Surgery, University of Perugia, Perugia, Italy; 2 Department of Life Science, Health, and Health Professions, Link Campus University, Rome, Italy; 3 Neurochemistry Laboratory, Department of Laboratory Medicine, Amsterdam Neuroscience, Amsterdam University Medical Center, Vrije Universiteit, Amsterdam, the Netherlands; 4 Translational AI in Laboratory Medicine, Department of Laboratory Medicine, Amsterdam UMC NL, Amsterdam, the Netherlands; 5 Australian E-Health Research Centre, CSIRO, Herston, Queensland Australia; 6 Memory Clinic, Department of Neurology, Charles University, Second Faculty of Medicine and Motol University Hospital, Prague, Czech Republic; 7 AI Technology for Life, Department of Information and Computing Sciences and Department of Biology, Utrecht University, Utrecht, the Netherlands; 8 Department of Neurology, Institut d’Investigacions Biomèdiques Sant Pau - Hospital de Sant Pau, Universitat Autònoma de Barcelona, Hospital de la Santa Creu i Sant Pau, Barcelona, Spain; 9 Department of Neurology, University of Ulm, Ulm, Germany; 10 German Center for Neurodegenerative Diseases (DZNE e.V.), Ulm, Germany; 11 Departamento de Matemática Aplicada a las TIC, Polytechnical University of Madrid, Madrid, Spain; 12 Clinical Memory Research Unit, Department of Clinical Sciences Malmö, Faculty of Medicine, Lund University, Lund, Sweden; 13 Neurology Clinic, Skåne University Hospital, Lund, Sweden; 14 Wallenberg Center for Molecular Medicine, Lund University, Lund, Sweden; 15 Reina Sofia Foundation Alzheimer Center, CIEN Foundation, ISCIII, Madrid, Spain; 16 The University of Melbourne, The Florey Institute, Melbourne, Victoria Australia; 17 Amsterdam Neuroscience, Neurodegeneration, Amsterdam University Medical Centers, Amsterdam, the Netherlands; 18 Alzheimer Center Amsterdam, Department of Neurology, Amsterdam University Medical Centers, Location VUmc, Amsterdam, the Netherlands; 19 CIBERNED, Network Center for Biomedical Research in Neurodegenerative Diseases, Madrid, Spain; 20 Department of Neurology, Martin-Luther University Halle-Wittenberg, Halle (Saale), Germany; 21 Lab of neuropathology, Neurobiobank, Institute Born-Bunge, Antwerp University, Edegem, Belgium; 22 Reference Center for Biological Markers of Dementia (BIODEM), Laboratory of Neurochemistry and Behavior, Institute Born-Bunge, University of Antwerp, Antwerp, Belgium; 23 Vrije Universiteit Brussel, Center for Neurosciences (C4N), Neuroprotection and Neuromodulation Research Group (NEUR), Brussels, Belgium; 24 Universitair Ziekenhuis Brussel, Department of Neurology and Bru-BRAIN, Brussels, Belgium; 25 Barcelonaβeta Brain Research Center (BBRC), Pasqual Maragall Foundation, Barcelona, Spain; 26 Hospital del Mar Research Institute, Barcelona, Spain

**Keywords:** Diagnostic markers, Proteomics, Ageing

## Abstract

Blood-based biomarkers are emerging as scalable tools for the diagnosis and monitoring of neurodegenerative diseases, but markers enabling differential diagnosis across major dementias remain limited. Here we show that large-scale plasma proteomics identifies disease-associated signatures across Alzheimer’s disease, dementia with Lewy bodies and frontotemporal dementia. We analyzed 1,318 plasma samples from well-characterized international cohorts and identified more than 200 dysregulated proteins across disease groups. Glial fibrillary acidic protein showed the strongest increase along the Alzheimer’s disease continuum, whereas integrin alpha-V and integrin alpha-M were consistently reduced in Lewy body disorders, including autopsy-confirmed cases. Elevated neurofilament light chain and lower glial fibrillary acidic protein were associated with frontotemporal dementia. We translated these findings into a 21-protein quantitative multiplex panel and validated it in an independent multicenter cohort (*n* = 805). These findings support plasma proteomics as an approach for biomarker-based differential diagnosis and disease staging across major neurodegenerative dementias.

## Abstract (web summary)

A large multicenter proteomics study identified blood biomarkers that distinguish Alzheimer’s disease, dementia with Lewy bodies and frontotemporal dementia, supporting biomarker-based differential diagnosis of neurodegenerative diseases.

## Main

Different causes of dementia, such as Alzheimer’s disease (AD), dementia with Lewy bodies (DLB) and frontotemporal dementia (FTD), present substantial diagnostic challenges because of their overlapping symptoms, especially in the prodromal phases^1^. Thus, biomarkers are essential to reach an early and accurate diagnosis and to facilitate the choice of adequate treatments. Blood-based biomarkers offer several advantages, including their minimally invasive nature, cost-effectiveness and ease of repeated sampling, making them particularly suitable for large-scale diagnostic use and monitoring over time. There are already effective blood-based biomarkers for detecting the main AD pathological hallmarks (that is, amyloid and Tau pathology)^2^. However, fluid biomarkers for AD staging or for reliably identifying FTD and DLB are still lacking. This poses a major challenge in distinguishing between AD, DLB and FTD, as these conditions often exhibit overlapping clinical symptoms and considerable heterogeneity, especially in the early stages or in atypical presentations. The presence of coexisting pathologies further complicates the diagnostic process by blurring disease-specific features and reducing diagnostic specificity^3^. In this blood Proteins for early Discrimination of dEmentias (bPRIDE) project, we aimed to identify specific diagnostic protein signatures associated to each dementia type by profiling the plasma proteome of six international cohorts, including controls (*n* = 374) and patients at different clinical stages of AD (*n* = 532), DLB (*n* = 196) and FTD (*n* = 216) with a large-scale proximity extension assay (PEA). Results were translated into a single multiplex tool for absolute quantification of selected plasma proteins and validated in an independent cohort of 876 samples from seven centers encompassing controls (*n* = 108) and different prodromal and clinical stages of AD (*n* = 395), DLB (*n* = 142) and FTD (*n* = 160). We also evaluated the association of the most promising candidates and key neuropathological hallmarks using independent and previously generated PEA data from the autopsy-confirmed Queen Sofia Foundation’s Vallecas Alzheimer Project (VARS) (*n* = 129). PEA data from the Parkinson’s Progression Markers Initiative 9000 study (PPMI) (*n* = 221) were also used to further investigate the association of DLB biomarker candidates with Parkinson’s disease (PD).

## Results

### Project workflow and cohort description

**Fig. 1:**
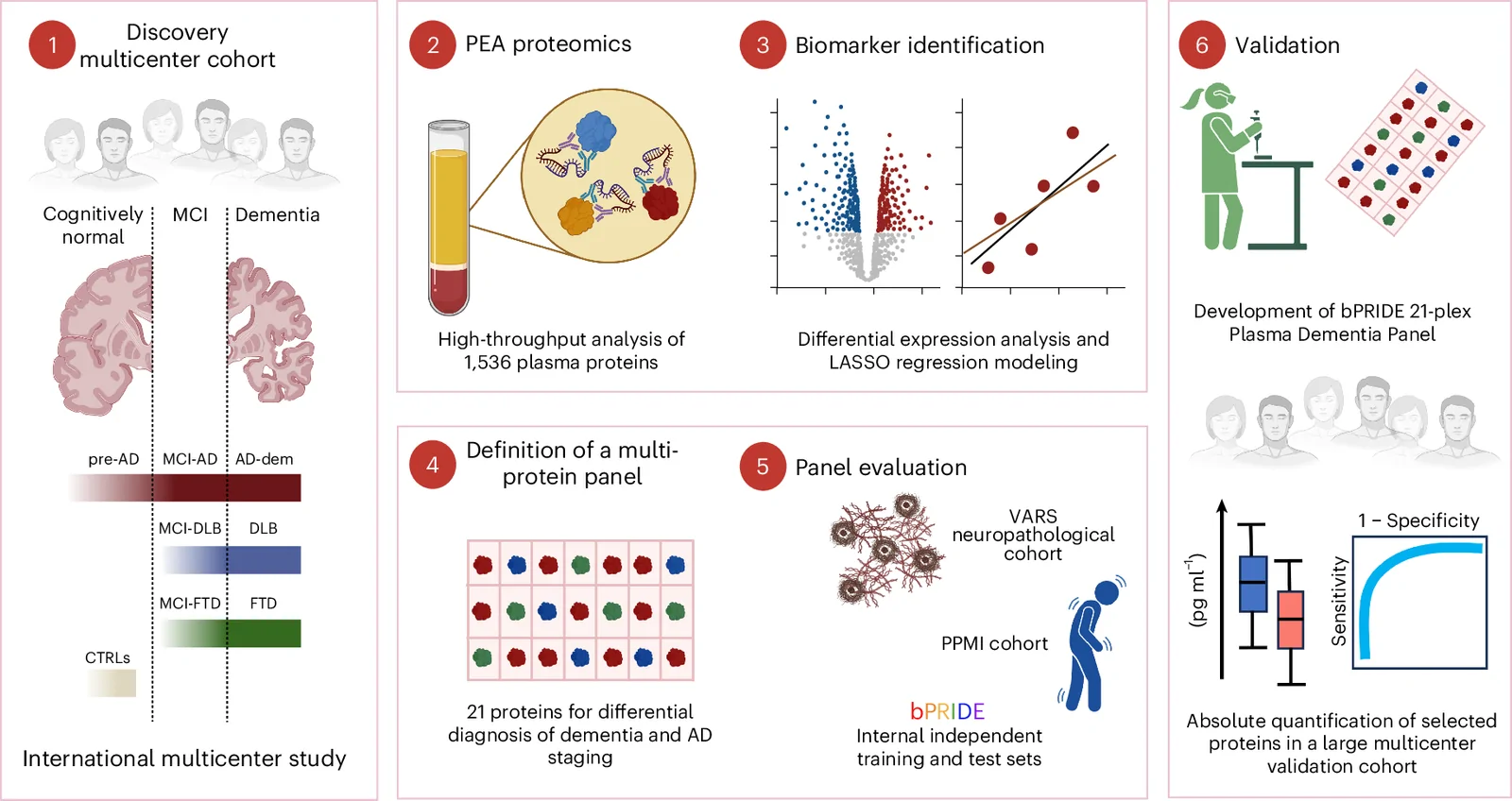
Overview of the bPRIDE study design. Schematic representation of the overall study workflow. Plasma samples from international cohorts underwent large-scale proteomic profiling, followed by quality control, differential protein expression analyses, machine learning, pathway analyses, neuropathological validation, and independent validation using a custom targeted biomarker panel. Aβ-PET, amyloid positron emission tomography. Created in BioRender; Hok-a-hin, Y. https://biorender.com/98ev0aa (2026).

Figure 1 summarizes the bPRIDE project workflow. Briefly, the final bPRIDE cohort included 1,277 participants (after precleaning; see the ‘Plasma protein profiling’ section in Methods for details), with 1,016 unique plasma protein measurements considered, including patients with AD ranging from preclinical to dementia stage, individuals with DLB and FTD at mild cognitive impairment (MCI) and dementia stage, as well as controls (CTRLs) selected from six different clinical neurological centers (see ‘Participants and inclusion criteria’ section in Methods for details). Univariate and multivariate pairwise group comparisons were performed to identify specific biomarkers or biomarker combinations for AD, DLB and FTD, as well as AD staging biomarkers that could be subsequently combined into a 21-plex PEA biomarker panel focused on discriminating dementia types. The custom bPRIDE Plasma Dementia Panel was tested on an independent validation cohort of 722 individuals (after precleaning). AD-associated and DLB-associated markers were further evaluated on independent PEA data generated in the neuropathological VARS cohort and in the PPMI cohort.

A summary of demographic and clinical features and the distribution of clinical groups included in the discovery and validation cohorts across centers are reported in Table 1. Demographic and pathological information regarding the external autopsy VARS cohort and the PPMI cohort are reported in Supplementary Tables 1 and 2, respectively. Mini-Mental State Examination (MMSE) scores progressively declined from the CTRL and preclinical AD (pre-AD) groups to the dementia groups, with the MCI groups showing intermediate values. The CTRL, MCI-FTD and FTD groups were slightly younger than the pre-AD, MCI-AD, AD at dementia stage (AD-dem) and DLB; however, in general, the groups were well balanced in age. Sex prevalence varied significantly among the groups: the highest percentage of male individuals was in the DLB and MCI-DLB groups, the highest percentage of females was in the AD groups. CTRLs had a sex prevalence similar to that of AD. In line with previous neuropathological studies^4^, approximately 50% of DLB and MCI-DLB, as well as 15% in FTD and MCI-FTD, cases had positive biomarkers of Aβ pathology. In the discovery cohort, principal component analysis applied to scaled proteomic data revealed significant center-associated effects (Supplementary Fig. 1a,b), which were partially driven by group imbalances among centers, that is, most CTRL samples were derived from the BioFINDER and Australian Imaging, Biomarkers & Lifestyle (AIBL) flagship study of ageing cohorts, which mainly consisted of patients within the AD-cont. In the validation cohort, we observed that the overall proteomic distribution of the autopsy Institute Born-Bunge (IBB) cohort completely differed from the rest (Supplementary Fig. 1c,d). Considering also its limited sample size, this cohort has been analyzed separately.

**Table 1:** Characteristics of the bPRIDE multicenter discovery and validation cohorts

Discovery (total *n* = 1,277)													
Group	*n*	Age (years)	Sex (% female)	MMSE	Aβ^+^ (%)	AIBL (%)	SPIN (%)	UU (%)	UNIPG (%)	BioFINDER (%)	ADC (%)	CBAS (%)	IBB (%)
CTRLs	362	69.2 ± 6.5	63%	28.9 ± 1.1	0%	7%	6%	3%	10%	68%	6%	0%	0%
Pre-AD	183	71.9 ± 6	67%	28.5 ± 1.3	100%	27%	6%	2%	7%	48%	10%	0%	0%
MCI-AD	157	71.8 ± 6	57%	26.2 ± 2.1	100%	23%	12%	15%	13%	23%	14%	0%	0%
AD-dem	175	70.6 ± 7.5	62%	20.3 ± 4.9	100%	21%	11%	27%	29%	0%	12%	0%	0%
MCI-DLB	25	70.8 ± 6	40%	25.8 ± 2.9	52%	0%	60%	0%	12%	0%	28%	0%	0%
DLB	167	72 ± 6.1	30%	21.7 ± 4.5	55%	0%	38%	7%	2%	0%	53%	0%	0%
MCI-FTD	45	68.2 ± 6	36%	25.8 ± 2.8	13%	0%	16%	9%	64%	0%	11%	0%	0%
FTD	163	68.1 ± 8	43%	22.6 ± 5.1	4%	0%	35%	39%	3%	0%	23%	0%	0%
Validation (total *n* = 722)													
CTRLs	96	68.4 ± 7.4	58%	28.8 ± 6.6	0%	20%	19%	5%	17%	0%	22%	18%	0%
Pre-AD	92	71.4 ± 5.4	49%	28.5 ± 1.3	100%	40%	2%	0%	13%	0%	36%	9%	0%
MCI-AD	109	72.9 ± 5.6	52%	25.1 ± 6.9	100%	13%	20%	0%	20%	0%	20%	27%	0%
AD-dem	109	72 ± 5.9	57%	19.3 ± 7.2	100%	19%	24%	0%	18%	0%	20%	18%	0%
MCI-DLB	30	73.9 ± 14.6	43%	26 ± 9.4	38%	0%	50%	0%	0%	0%	0%	50%	0%
DLB	81	71 ± 6.9	22%	21.8 ± 7.5	49%	0%	7%	0%	16%	0%	64%	12%	0%
MCI-FTD	32	67.7 ± 8.6	47%	26.5 ± 9.6	9%	0%	28%	0%	0%	0%	0%	72%	0%
FTD	109	68.5 ± 7.3	40%	23 ± 11.5	5%	0%	18%	28%	22%	0%	14%	18%	0%
AD (autopsy)	56	75.7 ± 9.1	43%			0%	0%	0%	0%	0%	0%	0%	100%
DLB (autopsy)	8	72 ± 4.4	0%			0%	0%	0%	0%	0%	0%	0%	100%

Continuous variables are reported as the mean ± s.d. Categorical variables are reported as percentages. Aβ^+^, positive marker (imaging or CSF) of brain amyloidosis. ADC, Amsterdam Dementia Cohort; AIBL, Australian Imaging, Biomarkers & Lifestyle flagship study of ageing cohort; BioFINDER, Swedish BioFINDER study cohort; CBAS, Czech Brain Ageing Study; DLB, dementia with Lewy bodies; FTD, frontotemporal dementia; IBB, Institute Born-Bunge^49^; MCI-AD, mild cognitive impairment because of AD; MCI-DLB, mild cognitive impairment because of Lewy body disease; MCI-FTD, mild cognitive impairment because of frontotemporal degeneration; MMSE, Mini-Mental State Examination; pre-AD, preclinical AD; SPIN, Sant Pau Initiative on Neurodegeneration; UU, University of Ulm cohort; UNIPG, University of Perugia cohort.

### Identification of plasma biomarkers for AD

**Fig. 2:**
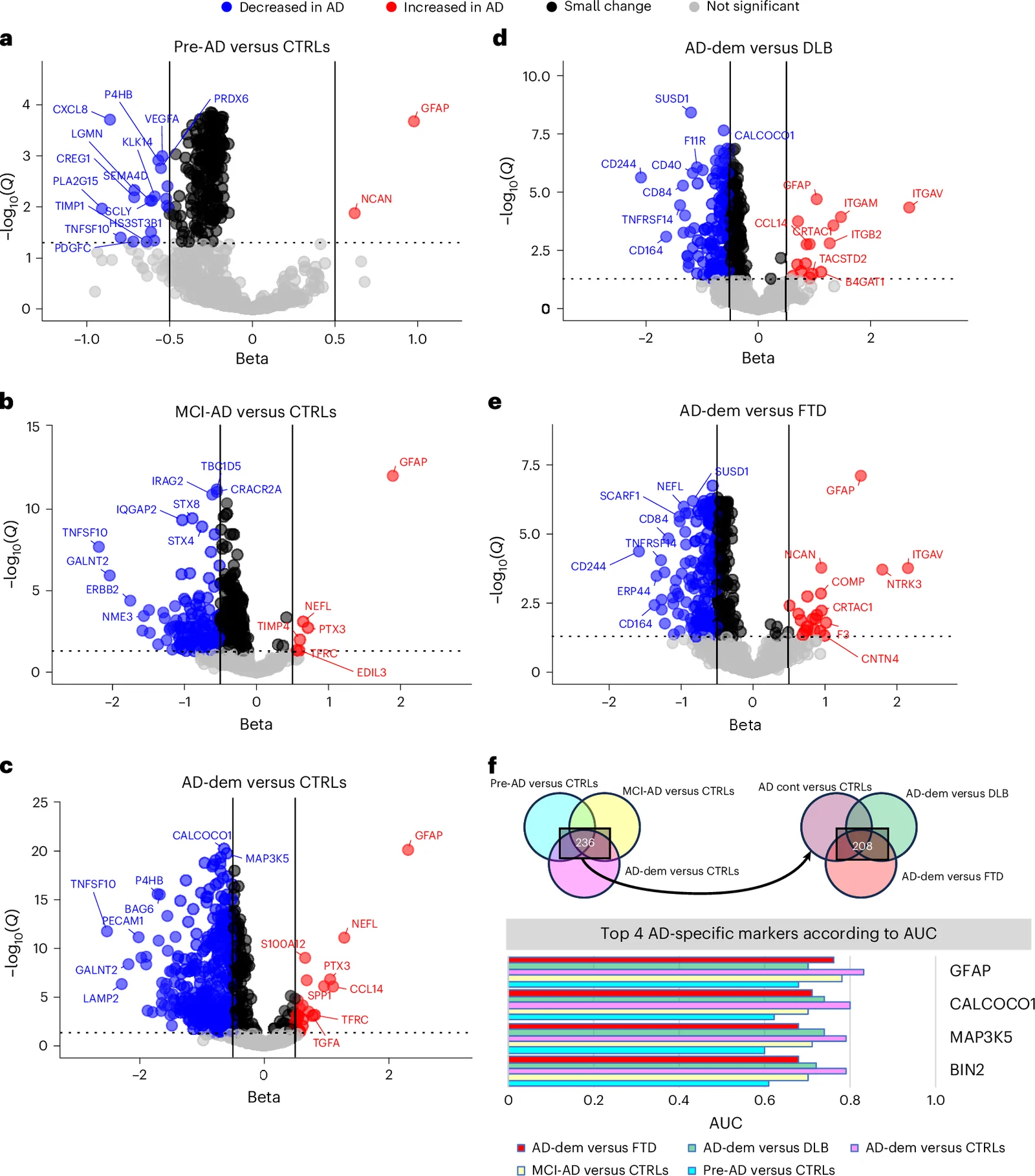
Plasma biomarkers dysregulated in AD. **a**–**e**, Volcano plots displaying −log_10_ of *Q* values (*P* values adjusted for age, sex and multiple comparisons) on the *y* axis and coefficients (Beta) resulting from applying logistic regression on *z*-scored normalized protein expression (NPX) data for pre-AD (*n* = 183) versus CTRLs (*n* = 362) (**a**), MCI-AD (*n* = 157) versus CTRLs (**b**), AD-dem (*n* = 175) versus CTRLs (**c**), AD-dem versus DLB (*n* = 163) (**d**) and AD-dem versus FTD (*n* = 167) (**e**). The horizontal line refers to the significance threshold (*Q* = 0.05); the two vertical lines represent arbitrary thresholds (±0.5) for Beta. **f**, Venn diagrams summarizing the proteins significantly dysregulated in each comparison; the AUC values of the four proteins showing the greatest average AUC among comparisons is also reported. Proteins are represented by gene names that encode them. Source data

Plasma proteome profiling revealed a total of 236 consistently dysregulated proteins across AD stages (preclinical, prodromal/MCI and dementia) compared to CTRL (Fig. 2a–c,f), from which 88% (208 proteins) were also significantly altered in AD-dem compared to DLB and FTD (Fig. 2d–f). Most of the proteins identified were downregulated across the AD continuum (>99%; Fig. 2a–e and Supplementary Table 3), only glial fibrillary acidic protein (GFAP) was consistently and significantly increased in AD in every comparison. The proteins showing the highest discriminative performance, based on average area under the curve (AUC), among the AD continuum (AD-cont) versus CTRL, AD-dem versus DLB and AD-dem versus FTD were GFAP, CALCOCO1, BIN2 and MAP3K5 (Fig. 2f).

**Extended Data Fig. 1:**
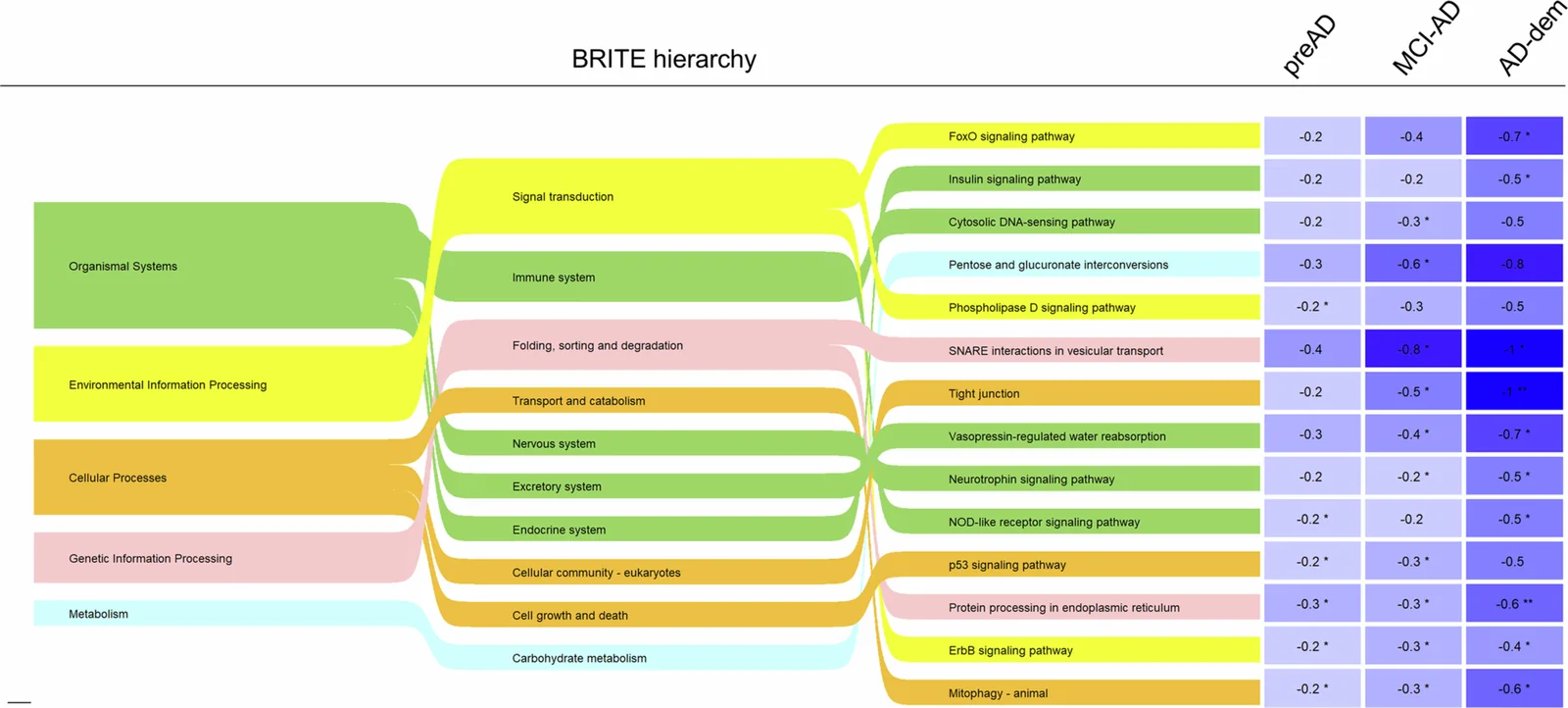
Enriched KEGG pathways and BRITE hierarchy for the AD continuum. The plot shows significantly enriched pathways (minimum of 3 measured proteins) comparing preAD, MCI-AD, and AD-dem with the CTRL group. The left panel (Sankey plot) illustrates the hierarchical classification of significantly enriched pathways based on the BRITE system, which organizes individual pathways into main classes and two levels of subclasses from left to right. Main classes are ranked according to the total number of associated pathways, with the largest class positioned at the top. The right panel summarizes the median effect (beta) for each pathway, with darker blue indicating stronger downregulation relative to controls and white indicating a median effect size of 0. Pathways are ordered according to the number of diagnostic groups in which they show significant enrichment. Exact p-values and FDR-adjusted p-values for all pathway enrichments are provided in the Source Data file. Source data

Pathway enrichment analysis revealed 14 pathways significantly enriched (false discovery rate (FDR) < 0.05) within at least one of the pre-AD, MCI-AD and AD-dem groups (Extended Data Fig. 1). All pathways were downregulated compared to CTRLs, and their effect was stronger along AD disease progression (that is, highest effect sizes in the AD-dem group). Three of the 14 significantly enriched pathways, including protein processing in the endoplasmic reticulum, ErbB signaling and mitophagy were significantly downregulated across the three AD clinical stages. Four of the pathways (SNARE interaction in vascular transport, tight junction, vasopressin-regulated water resorption and neurotrophin signaling) were significantly enriched only in the MCI and dementia AD groups. Two pathways (FoxO signaling and insulin signaling) were significantly enriched only in AD-dem. There were pathways dysregulated only in the pre-AD or MCI-AD groups, but with modest effects (that is, less than 0.4).

### Identification of biomarkers for the molecular staging of AD

**Fig. 3:**
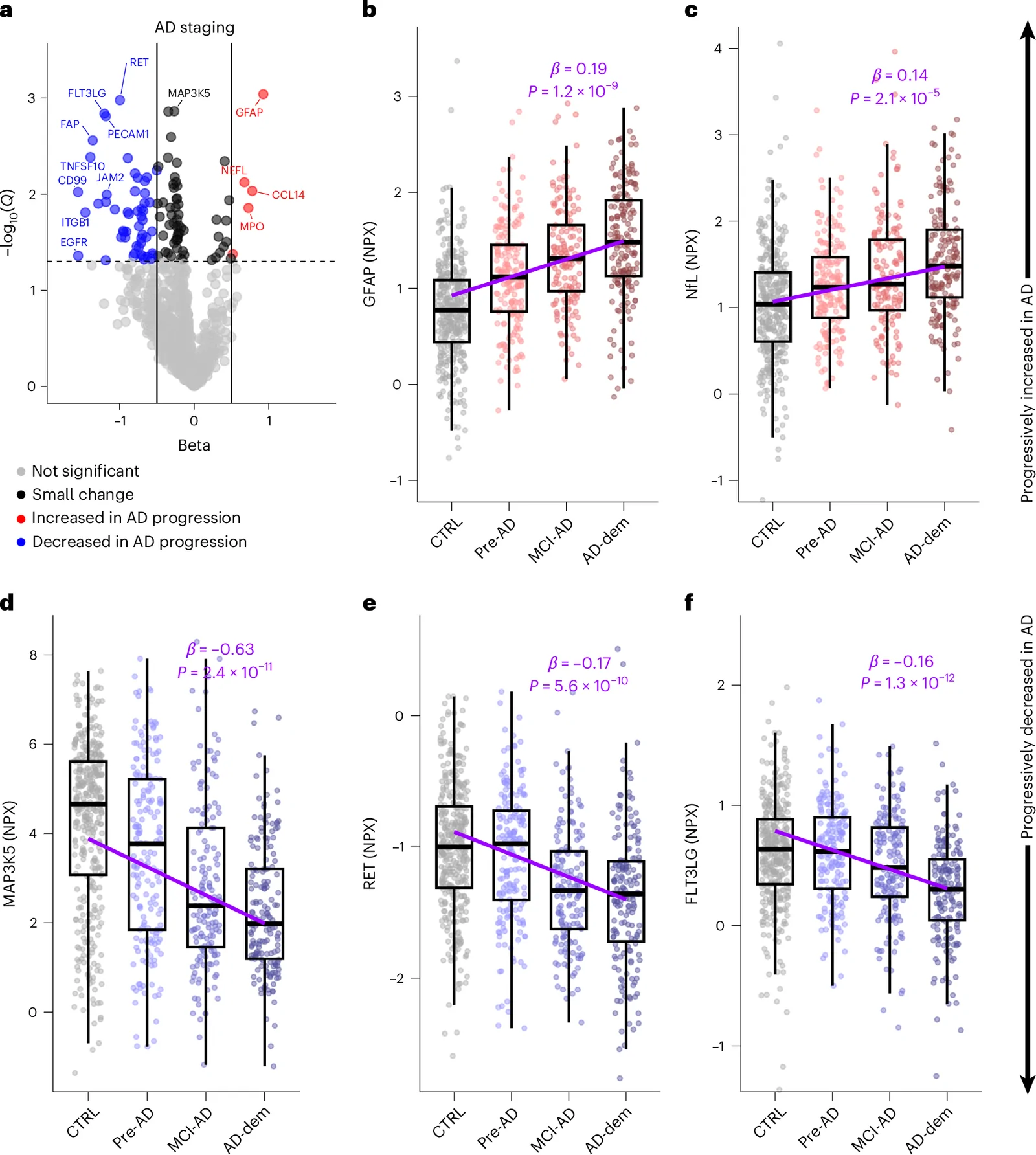
Biomarkers associated with AD clinical stage. **a**, Volcano plots displaying the average −log_10_ of *Q* values (*P* values adjusted for age, sex and multiple comparisons) on the *y* axis and average coefficients (Beta) resulting from applying logistic regression on *z*-scored NPX data for the pre-AD (*n* = 183) versus MCI-AD (*n* = 157), MCI-AD versus AD-dem (*n* = 175) and pre-AD versus AD-dem. The horizontal line refers to *Q* = 0.05; the two vertical lines represent arbitrary thresholds (±0.5) for Beta. **b**–**f**, Box plots representing raw NPX data for proteins displaying the greatest potential for the molecular staging of AD. GFAP (**b**) and NfL (**c**) were the proteins showing the most pronounced increase from pre-AD to AD-dem; MAP3K5 (**d**), RET (**e**) and FLT3LG (**f**) showed the most pronounced decrease from pre-AD to AD-dem. The boxes indicate the interquartile range (IQR) with the internal horizontal line representing the median; whiskers extend to 1.5 times the IQR. A nonparametric Theil–Sen regression line fitting the data from pre-AD to AD-dem (CTRL added only for reference) was also added for visualization purposes. Regression slopes (*β*) and relative *P* values are also shown. Proteins are represented using the names of genes that encode them. Source data

Most of the proteins specifically dysregulated in AD showed a progressively increased or decreased abundance along clinical stages (Fig. 3a), suggesting that these plasma protein levels may have potential as staging markers for AD. GFAP and neurofilament light chain (NfL) were the proteins showing the largest increase from pre-AD to AD-dem (Fig. 3b,c). Conversely, MAP3K5, RET and FLT3LG were those showing the strongest decrease from pre-AD to AD-dem (Fig. 3d–f). Specifically, MAP3K5 showed a significant progressive decrease between all AD stages (Fig. 3d), RET showed changes starting from the MCI stage (Fig. 3e) and FLT3LG was specifically decreased at the dementia stage (Fig. 3f). In general, GFAP showed the most marked change from pre-AD to MCI-AD and from MCI-AD to AD-dem.

### Identification of plasma biomarkers for DLB

**Fig. 4:**
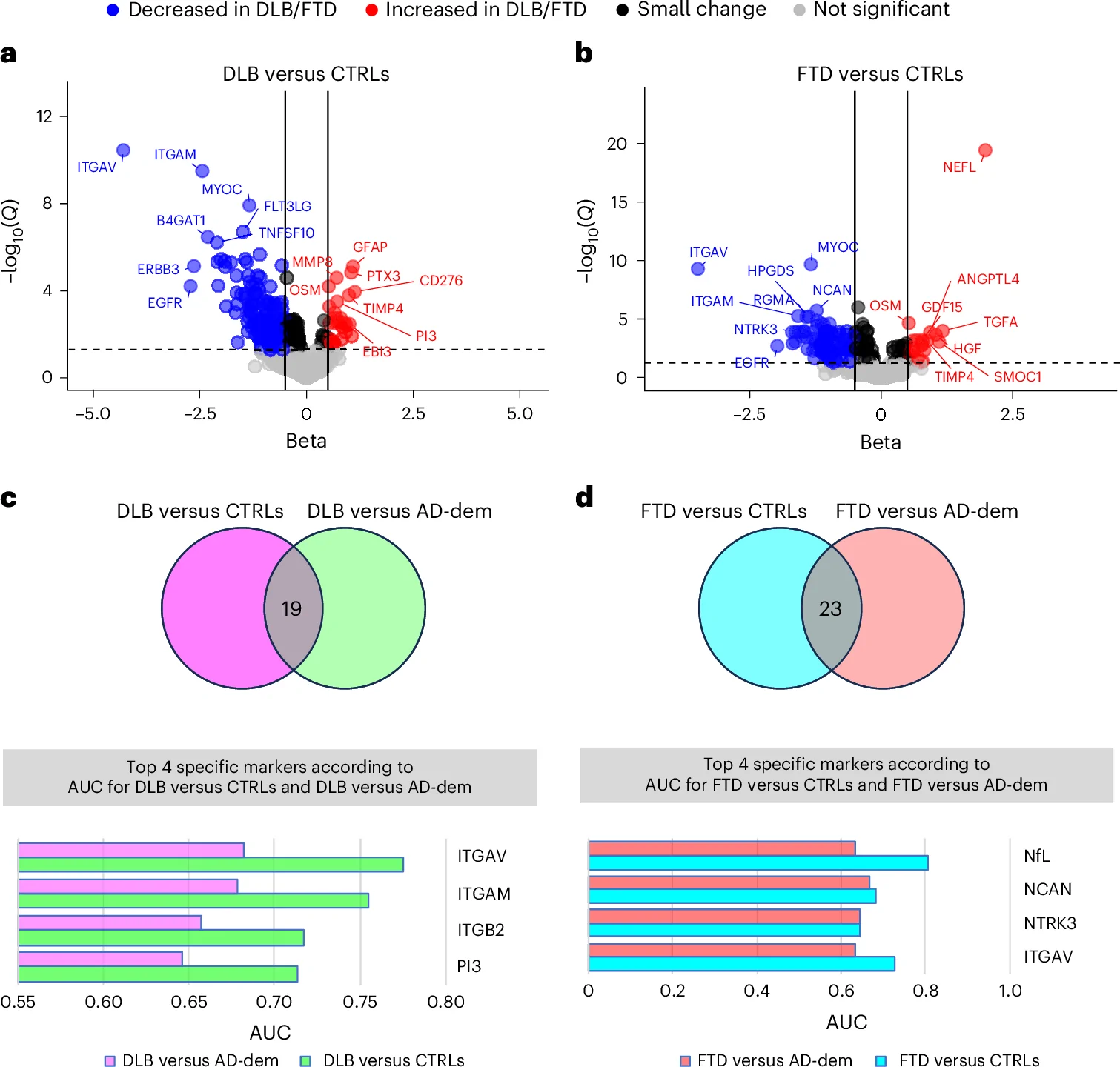
Plasma biomarkers associated with DLB and FTD. **a**,**b**, Volcano plots displaying −log_10_ of *Q* values (*P* values adjusted for age, sex and multiple comparisons) on the *y* axis and coefficients (Beta) resulting from applying logistic regression on *z*-scored NPX data for DLB (*n* = 167) versus CTRLs (*n* = 362) (**a**) and FTD (*n* = 163) versus CTRLs (**b**). The horizontal line refers to *Q* = 0.05; the two vertical lines represent arbitrary thresholds (±0.5) for Beta. **c**,**d**, Venn diagrams summarizing the proteins simultaneously dysregulated for DLB versus CTRLs and DLB versus AD-dem (**c**) and for FTD versus CTRLs and FTD versus AD-dem (**d**); the AUC values of the four proteins showing the greatest average AUC among comparisons is also reported. Proteins are represented using the names of the genes that encode them. Source data

**Extended Data Fig. 2:**
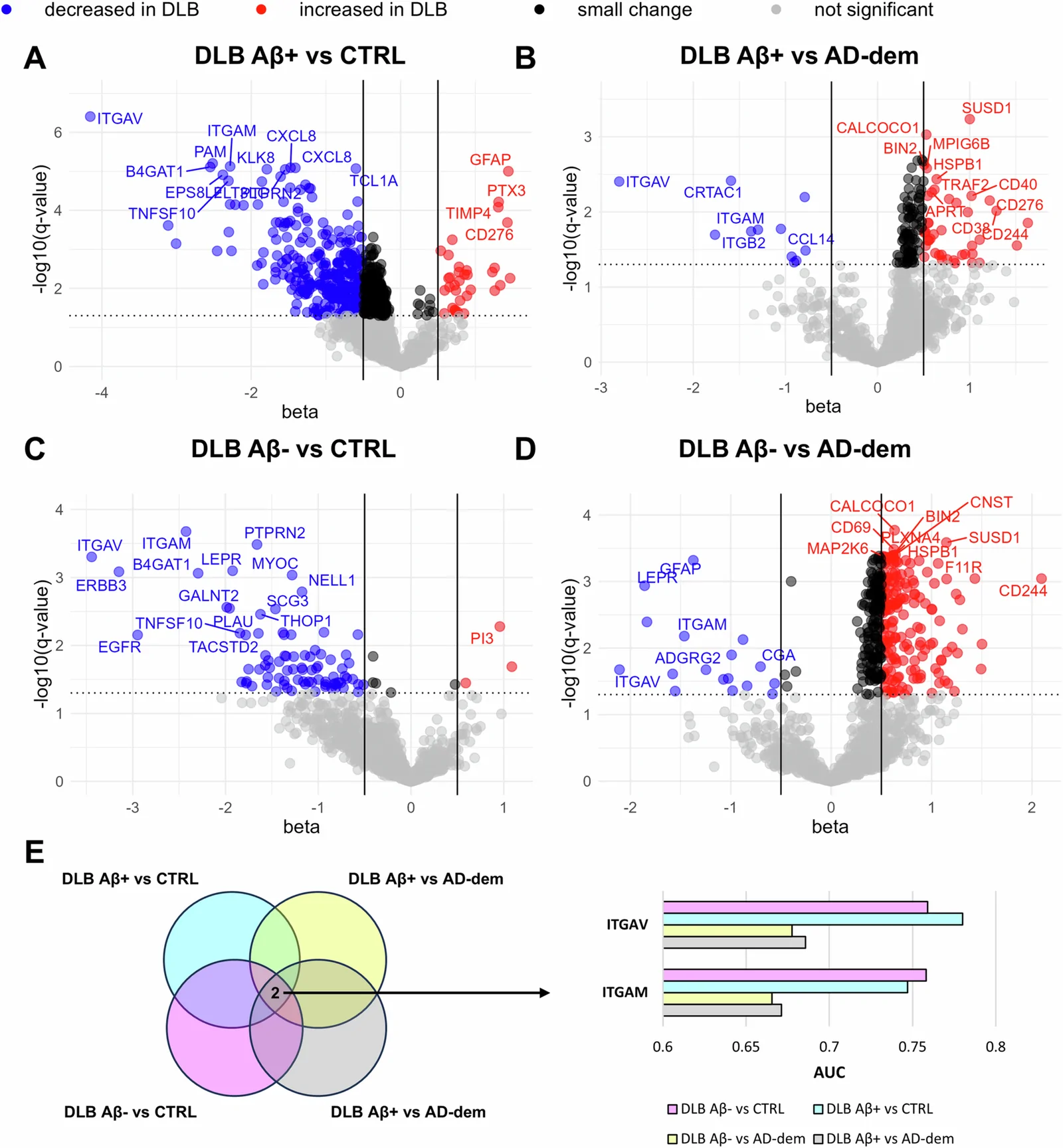
Specific markers for DLB with and without positive Aβ markers. **a–d**) Volcano plots displaying -log10 of q-values (p-values adjusted for age, sex, and multiple comparisons) on the y-axis and coefficients (beta) resulting from logistic regression on z-scored NPX data for DLB Aβ+ vs CTRL (n=86 vs n=362; A), DLB Aβ+ vs AD-dem (n=86 vs n=175; B), DLB Aβ− vs CTRL (n=70 vs n=362; C), and DLB Aβ− vs AD-dem (n=70 vs n=175; D). The horizontal line refers to q-value = 0.05, and the two vertical lines represent arbitrary thresholds (±0.5) for beta. **e**) Among the proteins considered, ITGAV and ITGAM were consistently downregulated in DLB Aβ± across all comparisons. The effect size (Cohen’s d) is reported for each comparison. Proteins are represented using the names of the genes encoding them. Other abbreviations include Aβ±: positive or negative biomarker of brain amyloidosis; AD: Alzheimer’s disease; AD-dem: AD at dementia stage; CTRL: controls; DLB: dementia with Lewy bodies. Source data

**Extended Data Fig. 3:**
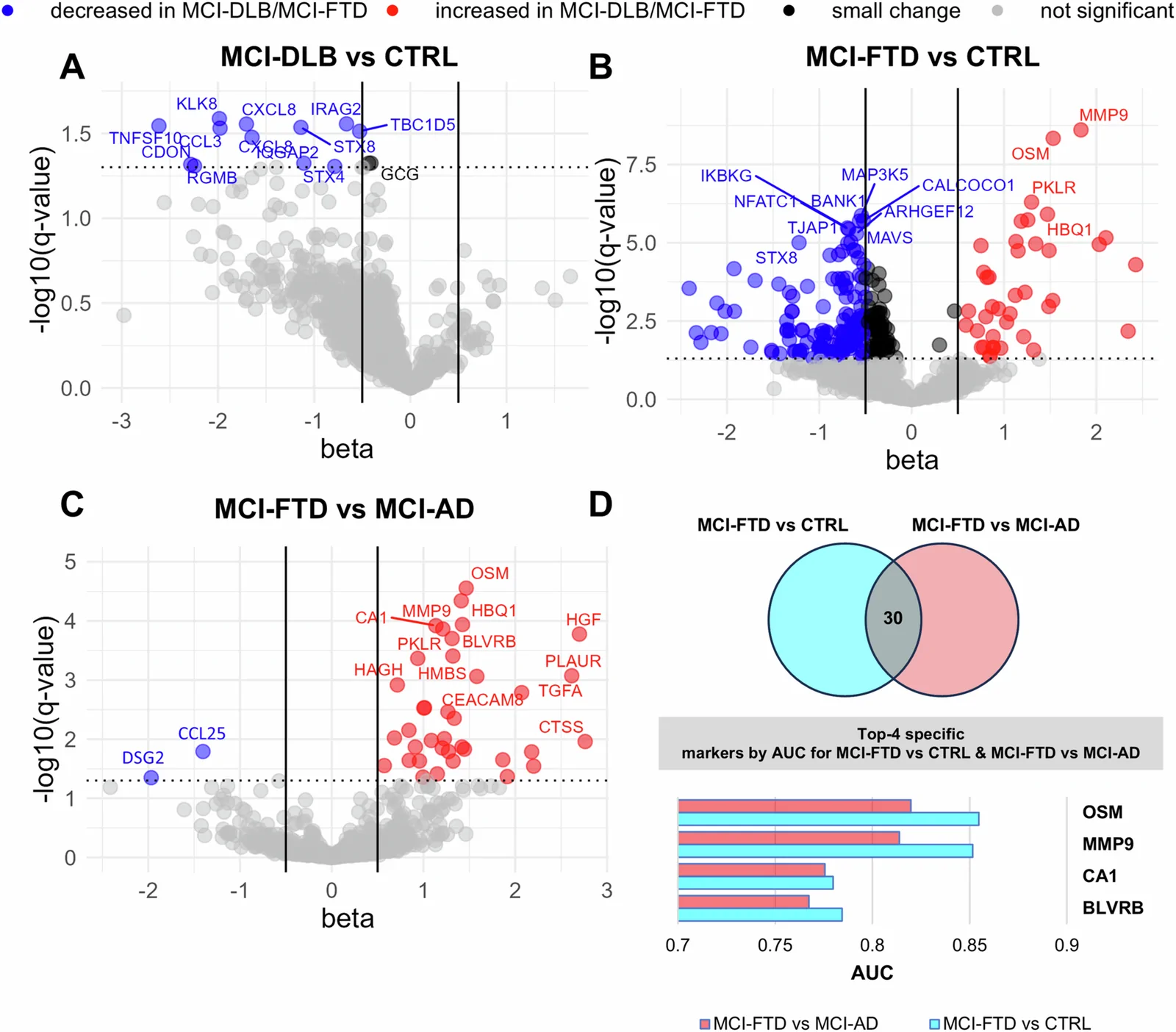
Specific markers for MCI-DLB and MCI-FTD. **a–c**) Volcano plots displaying -log_10_ of q-values (p-values adjusted for age, sex and multiple comparisons) on the y-axis and coefficients (beta) resulting from applying logistic regression on z-scored NPX data for: A) MCI-DLB (n=25) vs CTRL (n=362), B) MCI-FTD (n=45) vs CTRL, C) MCI-FTD vs MCI-AD (n=157). The horizontal line refers to q-value = 0.05, the two vertical represent arbitrary thresholds (± 0.5) for beta. None of the proteins analyzed showed a q < 0.05 for MCI-DLB vs MCI-AD. **d**) 31 proteins showed q < 0.05 for MCI-FTD vs CTRL and MCI-FTD vs MCI-AD. Among those, OSM, MMP9, CA1 and BLVRB were those showing the highest average AUC, which are shown with their effect size (Cohen’s d) for each comparison. Proteins are represented using the names of genes encoding for them, other abbreviations include AD: Alzheimer’s disease, AUC: area under the ROC curve, CTRL: controls, MCI-DLB: mild cognitive impairment due to Lewy body disease, MCI-FTD: mild cognitive impairment due to frontotemporal degeneration. Source data

We identified 71 plasma proteins dysregulated in DLB compared to CTRLs (Fig. 4a), from which 27% (19 proteins) were also dysregulated when compared to AD-dem (Fig. 4a,c and Supplementary Table 4). Again, most of the proteins were downregulated in DLB compared to CTRLs. The proteins showing the highest discriminative performance (based on the average AUC between DLB and CTRLs and AD-dem) were PI3 and three proteins from the integrin family, that is, integrin alpha-V (ITGAV), integrin alpha-M (ITGAM) and ITGB2 (Fig. 4c and Supplementary Table 4). As sensitivity analysis, we repeated the comparison by subgrouping DLB into DLB Aβ^+^ and DLB Aβ^−^ (Extended Data Fig. 2). In the stratified analysis, ITGAV and ITGAM were the only proteins significantly decreased in both DLB subgroups. For the MCI-DLB group, we found *n* = 18 proteins with decreased levels with respect to CTRLs (for example, STX8, IRAG2, TNFSF10 and KLK8), but none of these were differentially expressed when compared to MCI-AD (Extended Data Fig. 3), probably because of the limited group size.

No specific pathways were significantly enriched for the plasma proteins associated with DLB.

### Identification of plasma biomarkers for FTD

A total of 198 proteins were dysregulated in FTD compared with CTRLs (Fig. 4b), from which 12% (23 proteins) were also dysregulated when compared to AD-dem (Fig. 4d and Supplementary Table 5). The largest effect size was obtained for NfL, which was increased in FTD with respect to both CTRLs and AD-dem. This was followed by NCAN, NTRK3 and ITGAV, which were decreased in FTD (Fig. 4d and Supplementary Table 5). We detected 230 proteins dysregulated in MCI-FTD, from which 30 (13%) were also dysregulated when compared to the MCI-AD group (Extended Data Fig. 3). Levels of OSM, MMP9, CA1 and BLVRB generated the greatest average AUC across the two comparisons (AUC > 0.77; Extended Data Fig. 3). Of note, these proteins were not among the 23 proteins dysregulated at the FTD dementia stage, suggesting that they might be altered only in the early stages of the disease.

No specific pathways were significantly enriched for the proteins associated with FTD.

Finally, the comparison between DLB and FTD revealed only four proteins significantly dysregulated between these two groups (RGMA and DSG2 increased in DLB; SELE and NfL increased in FTD), from which NfL was the only one with a relatively large effect size (Supplementary Fig. 2).

### Multivariate plasma biomarker panel selection and impact of comorbidities on biomarker measurements

**Fig. 5:**
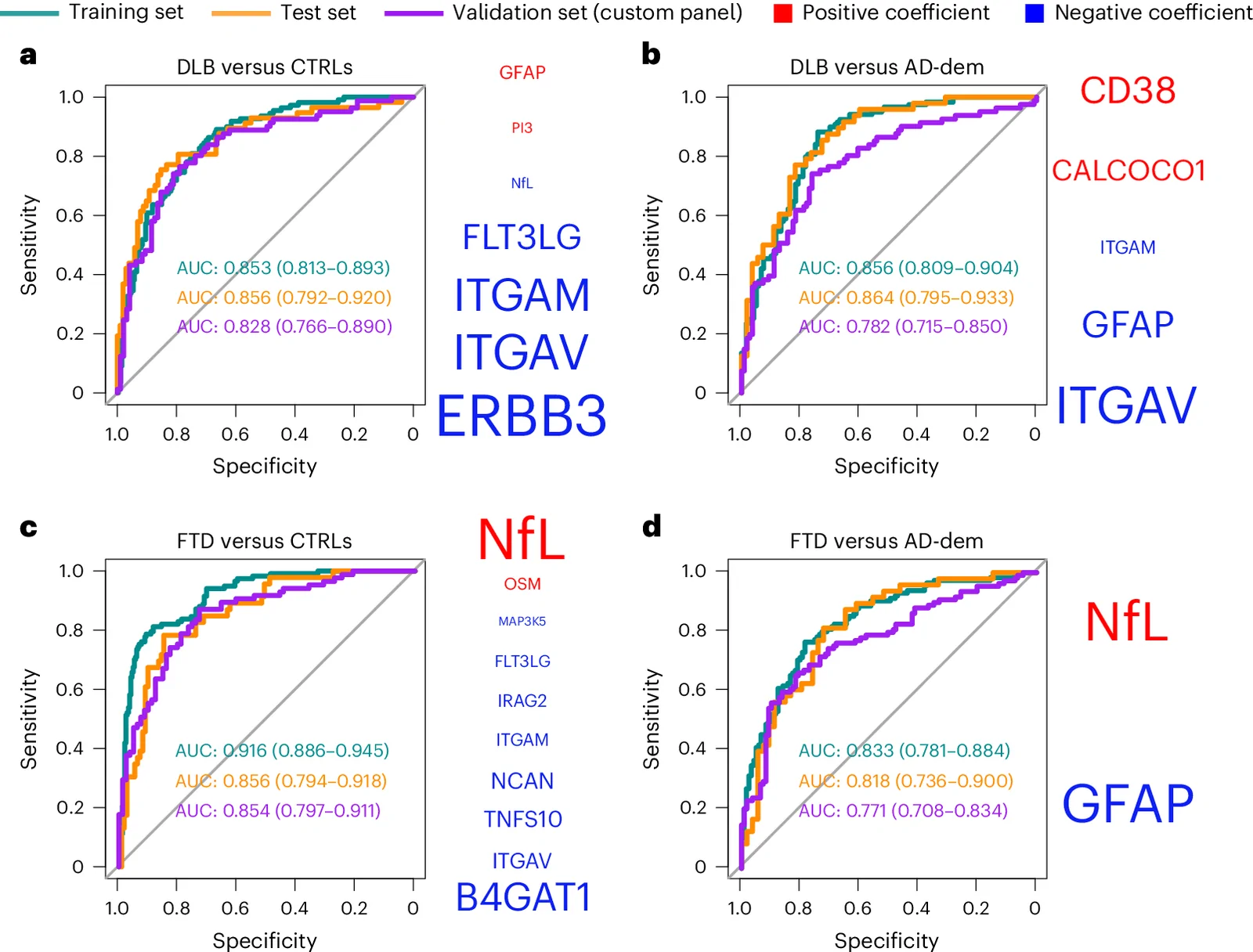
Diagnostic performance of multivariate models in the training and test sets in the discovery cohort and in the independent validation cohort. **a**–**d**, Receiver operating characteristic (ROC) curves for pairwise comparisons of clinical groups (AD-dem, DLB, FTD and CTRL) in the training (green), test (orange) and validation (purple) sets, the latter performed with a newly developed multiplex custom assay. AUC values with their 95% CIs are also displayed. The ROC curves were generated on the same training set used for panel selection and tested on an independent test set. For DLB versus AD-dem (**b**), 119 DLB and 120 AD-dem were used for model training, 48 DLB and 55 AD-dem were used for testing, and 81 DLB and 109 AD-dem were used for validation. For DLB versus CTRLs (**a**), 110 DLB and 260 CTRLs were used for training, 57 DLB and 102 CTRLs were used for testing, and 81 DLB and 96 CTRLs were used for validation. For FTD versus AD-dem (**d**), 115 FTD and 121 AD-dem were used for training, 48 FTD and 54 AD-dem were used for testing, and 109 FTD and 109 AD-dem were used for validation. For FTD versus CTRLs (**c**), 117 FTD and 250 CTRLs were used for training, 46 FTD and 112 CTRLs were used for testing and 109 FTD and 96 CTRLs were used for validation. The proteins contributing to each model are represented using the names of the genes that encode them with font size proportional to their relative coefficient in each model, and color indicative of coefficient sign. Source data

**Extended Data Fig. 4:**
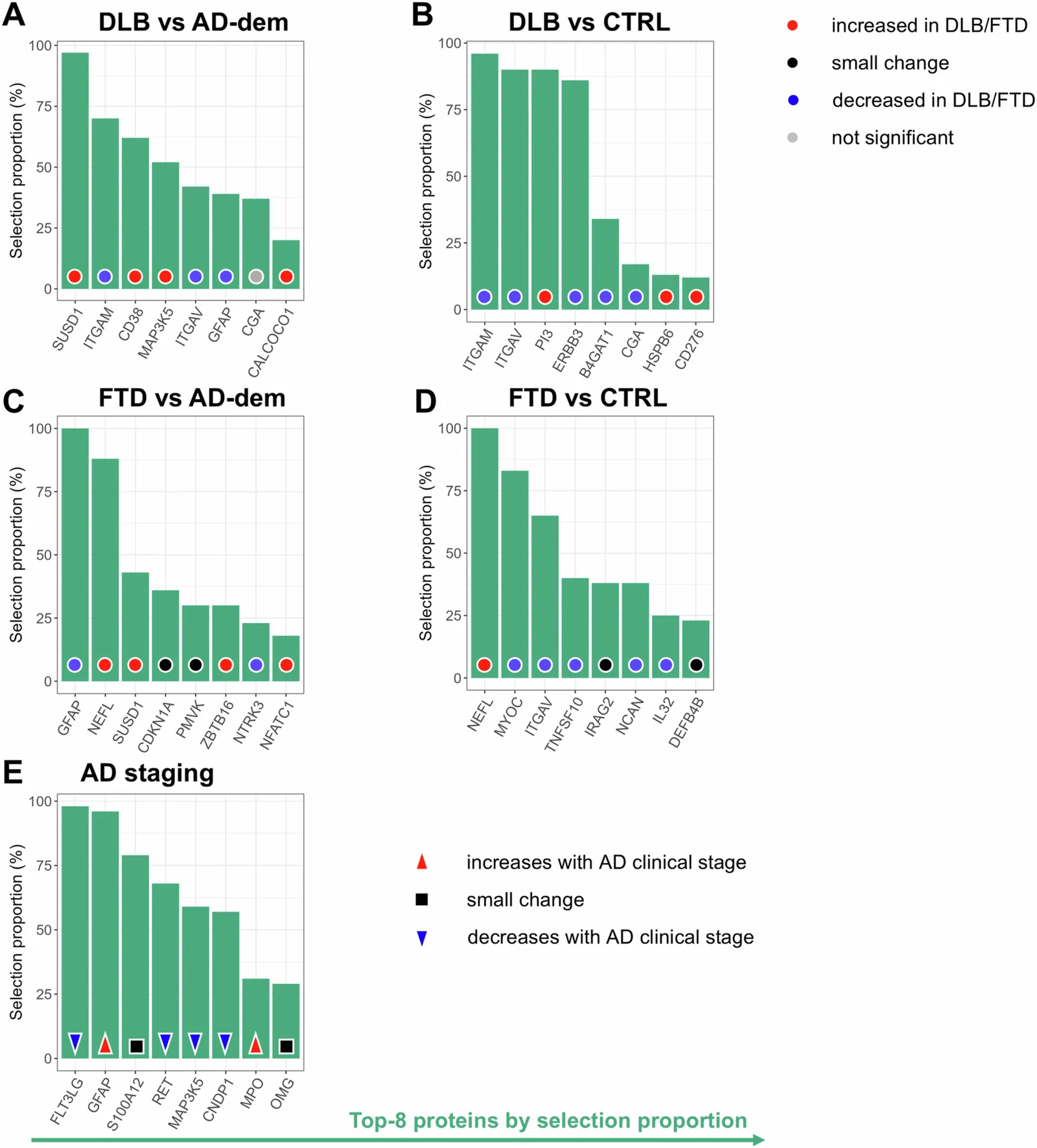
Protein selection within discovery training sets. For each of the DLB vs AD-dem (**a**), DLB vs CTRL (**b**), FTD vs AD-dem (**c**), and FTD vs CTRL (**d**) comparisons, we applied in training sets a 100-fold cross validated LASSO binomial regression and analyzed the selection proportion of PEA proteins in 5-variable models. To identify a composite biomarker for the molecular staging of AD (**e**), we applied Gaussian LASSO regression, using the clinical stages of AD as numeric variables (preAD=1, MCI-AD=2, AD-dem=3). For DLB vs AD-dem, n=119 DLB and n=120 AD-dem were used for model training, n=48 DLB and n=55 AD-dem were used for testing. For DLB vs CTRL, n=110 DLB and n=260 CTRL were used for training, n=57 DLB and n=102 CTRL were used for testing. For FTD vs AD-dem, n=115 FTD and n=121 AD-dem were used for training, n=48 FTD and n=54 AD-dem were used for testing. For FTD vs CTRL, n=117 FTD and n=250 CTRL were used for training, n=46 FTD and n=112 CTRL were used for testing. For AD staging, after filtering out samples with MMSE outside IQR, for model training, n=86 preAD, n=109 MCI-AD, and n=83 AD-dem were used. For model testing, n=41 preAD, n=48 MCI-AD, and n=38 AD-dem were used. Proteins are represented using the names of genes encoding for them, other abbreviations include AD: Alzheimer’s disease, AD-dem: Alzheimer’s disease at dementia stage, CTRL: controls, DLB: dementia with Lewy bodies, FTD: frontotemporal dementia, LASSO: least absolute shrinkage selection operator, MCI-AD: mild cognitive impairment due to AD, preAD: preclinical AD. Source data

**Extended Data Fig. 5:**
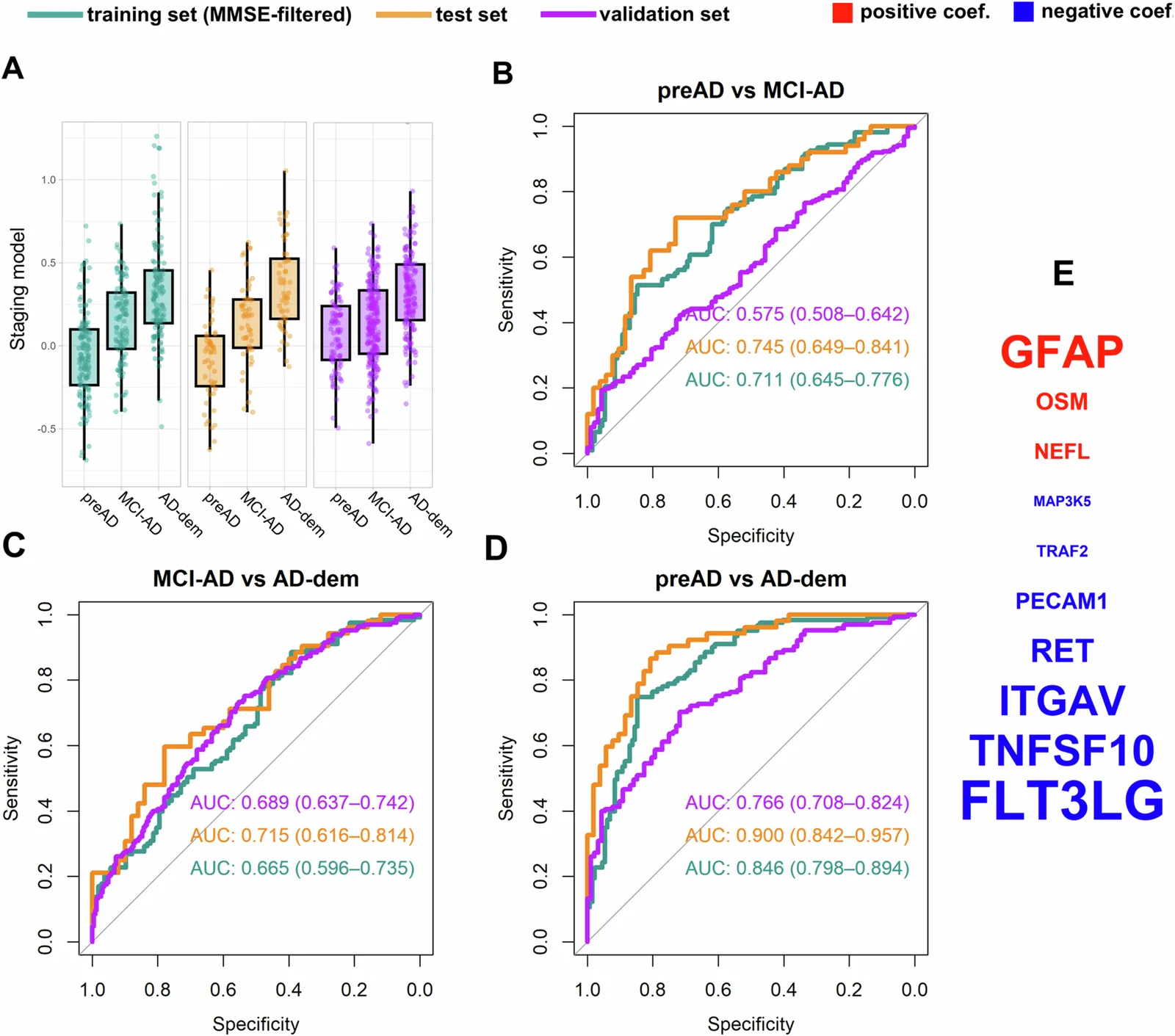
Performance of composite marker for AD staging. **a**) Boxplots representing the values of the staging model for clinical stages of AD in the training (MMSE-filtered), test and validation sets. The height of the boxes represents the interquartile range, with the horizontal line representing the median NPX value. Whiskers reflect the first/third quartile -/+ 1.5 times the interquartile range. **b–d**) ROC curves for pairwise comparisons of clinical stages of AD in training, test and validation sets, AUC values are displayed with their 95% CI. After filtering out samples with MMSE outside IQR, for model training, n=86 preAD, n=109 MCI-AD, and n=83 AD-dem were used. For model testing, n=41 preAD, n=48 MCI-AD, and n=38 AD-dem were used. For model validation, n=92 preAD, n=109 MCI-AD, and n=109 AD-dem were used, autopsy-confirmed cases were excluded. E) The proteins contributing to the staging model are represented using the names of genes encoding for them with font size proportional to their relative coefficient in the model, and color indicative of coefficient sign. Other abbreviations include AD: Alzheimer’s disease, AD-dem: AD at dementia stage, AUC: area under the ROC curve, CTRL: controls, MCI-AD: mild cognitive impairment due to AD, preAD: preclinical AD. Source data

Considering that most of the markers identified showed diagnostic performances (AUCs) below 0.8 (Supplementary Tables 3–5), for any of the comparisons considered, we next investigated whether a minimal combination of proteins could discriminate the groups of interest with higher performance. For AD, the plasma signature obtained did not outperform the current plasma biomarkers used to detect AD (that is, amyloid peptides and phosphorylated Tau forms), which were also analyzed in this same bPRIDE discovery cohort^2^. Thus, we prioritized identifying the minimum number of markers allowing specific identification of DLB and FTD, compared with CTRLs and AD, and molecular AD staging. Of note, 21 markers could be combined into a single PEA custom panel. Thus, we set a maximum of five proteins per model for each of the four purposes to identify the markers with high selection prevalence after 100-fold cross-validated least absolute shrinkage selection operator (LASSO) regression within a training set (70% of the bPRIDE discovery cohort; Methods and Extended Data Fig. 4). After identification of the most promising 21 proteins, multivariable logistic models were then trained in the same training set and validated in the independent test set (remaining 30% of the original dataset). The panel proteins used to generate the models, and their coefficients, are shown in Supplementary Table 6. We identified two plasma protein models that could discriminate DLB from CTRLs and AD with AUCs of 0.85 and 0.86, respectively, in both the training and test sets (Fig. 5a,b). ITGAV was one of the proteins that contributed most to the discrimination of DLB compared to both CTRLs and AD. ITGAM and ERBB3 were the main contributors to the discrimination of DLB from CTRLs (Fig. 5a), while CD38 substantially contributed to the discrimination of DLB from AD (Fig. 5b). For FTD, NfL together with B4GAT1 were the main contributors in a model that could discriminate FTD from CTRLs with an AUC of 0.92 (Fig. 5c). For the discrimination of FTD and AD, only NfL and GFAP were needed to achieve an AUC of 0.83 (Fig. 5d). For AD staging, we identified a ten-protein plasma model, in which GFAP together with FLT3LG and TNSF10 were the main contributors (Extended Data Fig. 5). Notably, all the models showed comparable performances in both the training and test sets in all the analyses performed.

**Extended Data Fig. 6:**
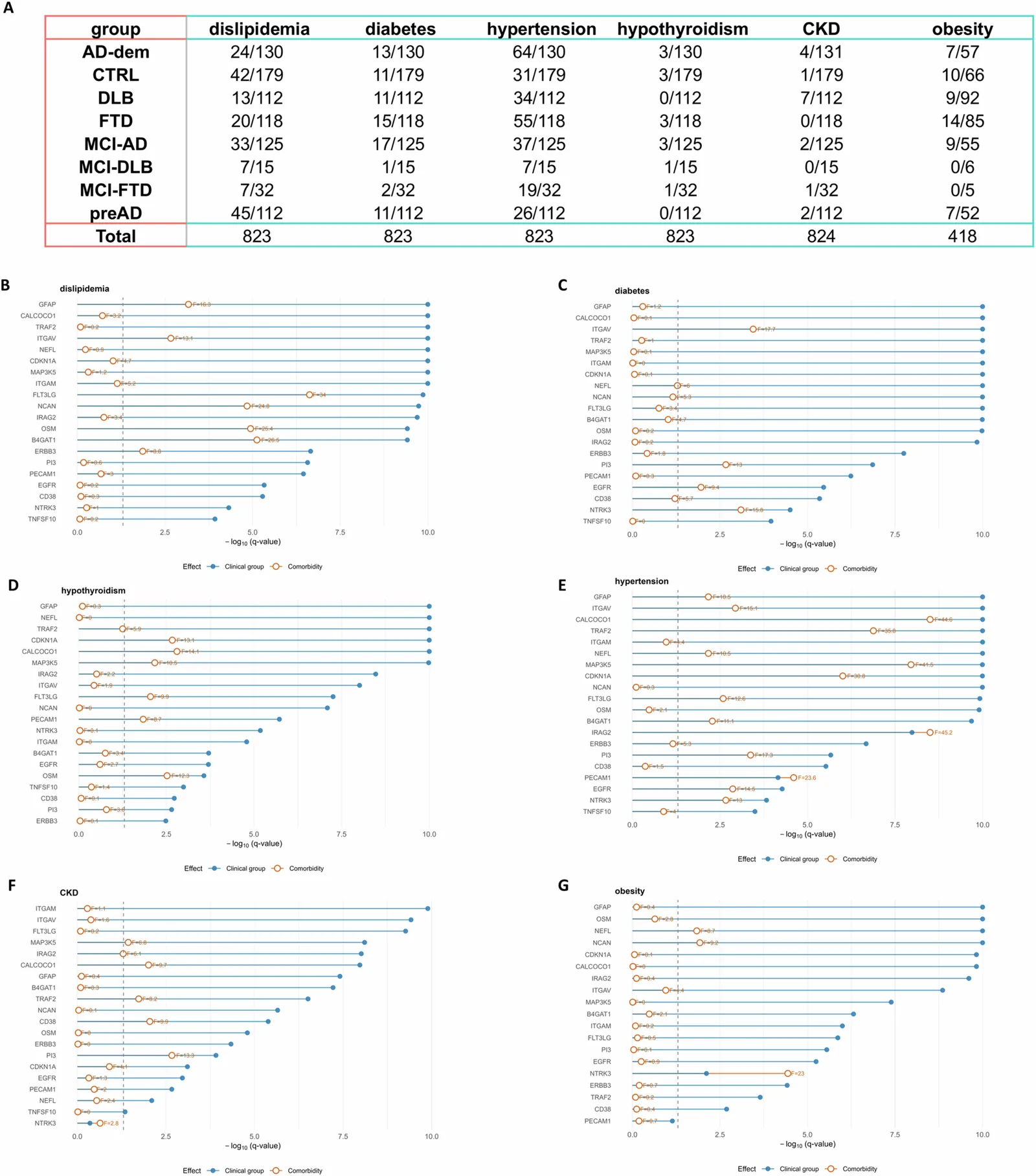
Impact of systemic co-morbidities on the 21 selected proteins within the discovery cohort. **a**) Summary table reporting the number of patients for whom anamnestic information about comorbidities was available together with the number of patients affected by each systemic condition. **b–g**) Lollipop plots showing the –log_10_(q-values) and F statistics for the effect of each comorbidity (orange markers) and –log_10_(q-values) for clinical group (blue markers) across proteins. Dashed vertical lines indicate the significance threshold (q = 0.05). Source data

We next evaluated the impact of relevant comorbidities (dyslipidemia, diabetes, hypertension, hypothyroidism, chronic kidney disease) on the levels of the selected proteins because they can influence the interpretation of blood-based biomarkers^5–7^. The selected proteins were largely unaffected by systemic comorbidities, with clinical group explaining most of the variance in protein levels. Only three proteins, NTRK3 (associated with obesity) and PECAM1 and IRAG2 (both associated with hypertension), showed significant comorbidity effects that were comparable or exceeded those produced by the disease group (Extended Data Fig. 6). These proteins were not among the main contributors driving discrimination across the different dementia types.

### Development and validation of custom multiplex PEA plasma protein panel

**Fig. 6:**
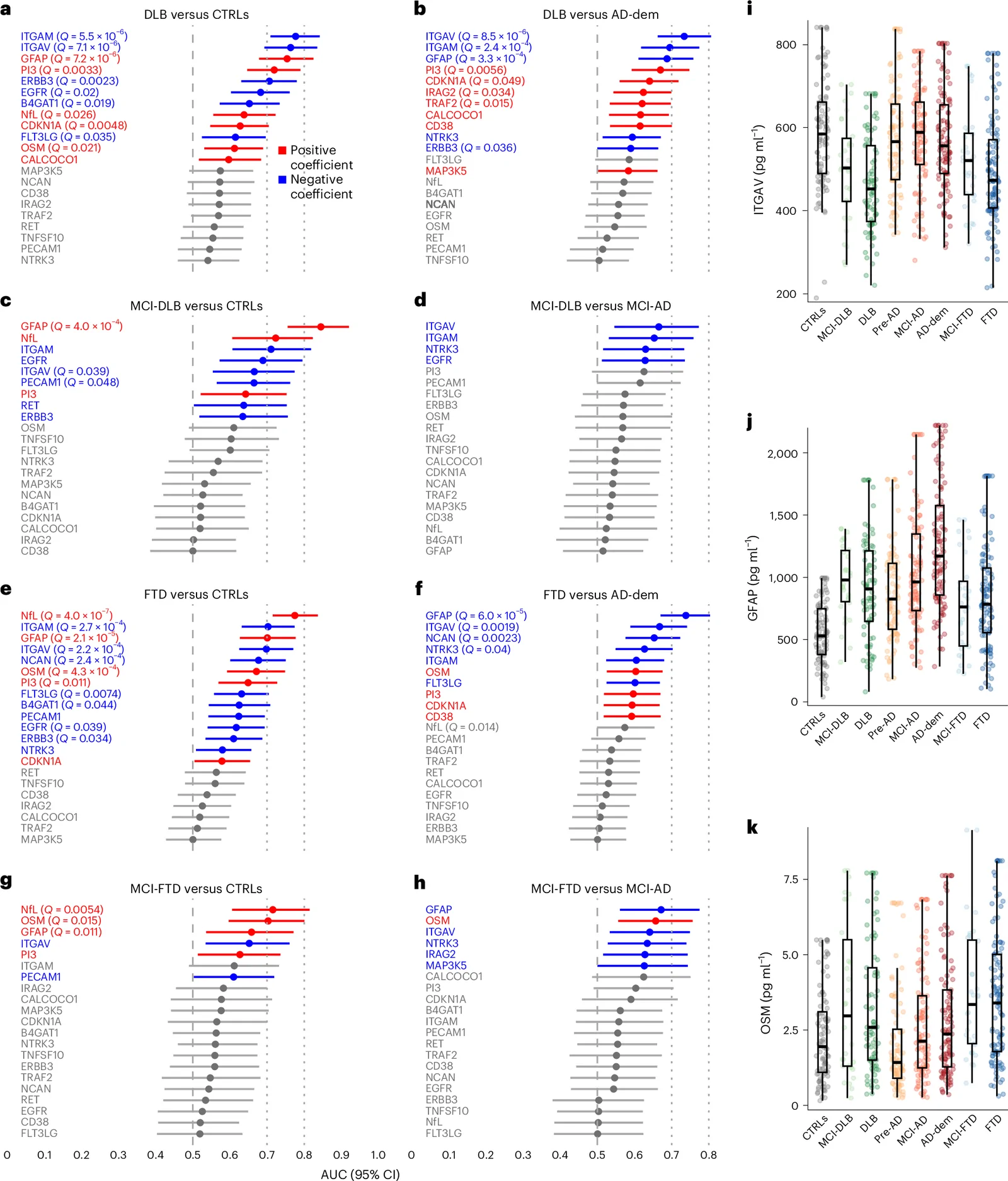
Diagnostic performance of the 21 plasma biomarkers and distribution of selected proteins in the validation cohort analyzed with the custom multiplex quantitative assay. **a**–**h**, Forest plots show the AUC values with 95% CIs for each of the 21 plasma biomarkers measured in the validation cohort. Comparisons include DLB versus CTRLs (*n* = 81 versus *n* = 96) (**a**), DLB versus AD-dem (*n* = 81 versus *n* = 109) (**b**), MCI-DLB versus CTRLs (*n* = 30 versus *n* = 96) (**c**) and MCI-DLB versus MCI-AD (*n* = 30 versus *n* = 109) (**d**), as well as the corresponding contrasts for FTD: FTD versus CTRLs (*n* = 109 versus *n* = 96) (**e**), FTD versus AD-dem (*n* = 109 versus *n* = 109) (**f**), MCI-FTD versus CTRLs (*n* = 32 versus *n* = 96) (**g**) and MCI-FTD versus MCI-AD (*n* = 32 versus *n* = 109) (**h**). Each point represents the mean AUC for an individual protein, with the horizontal bars indicating the 95% CIs estimated using 2,000 bootstrap replicates. *Q* values resulting from logistic regression models, including age as a covariate, with *P* values adjusted for multiple testing using the Benjamini–Hochberg method are reported next to the protein names for *Q* < 0.05. **i**–**k**, Box plots showing plasma concentrations (pg ml^−1^) of ITGAV (**i**), GFAP (**j**) and OSM (**k**) across diagnostic groups in the validation cohort. The boxes indicate the IQR, with the internal horizontal line representing the median; whiskers extend to 1.5 times the IQR. Values were clipped at the 95th percentile within each group for visual clarity. Plots for the remaining proteins within the custom multiplex assay can be found in Extended Data Fig. 8. Proteins are represented using the names of the genes that encode them. Source data

**Extended Data Fig. 7:**
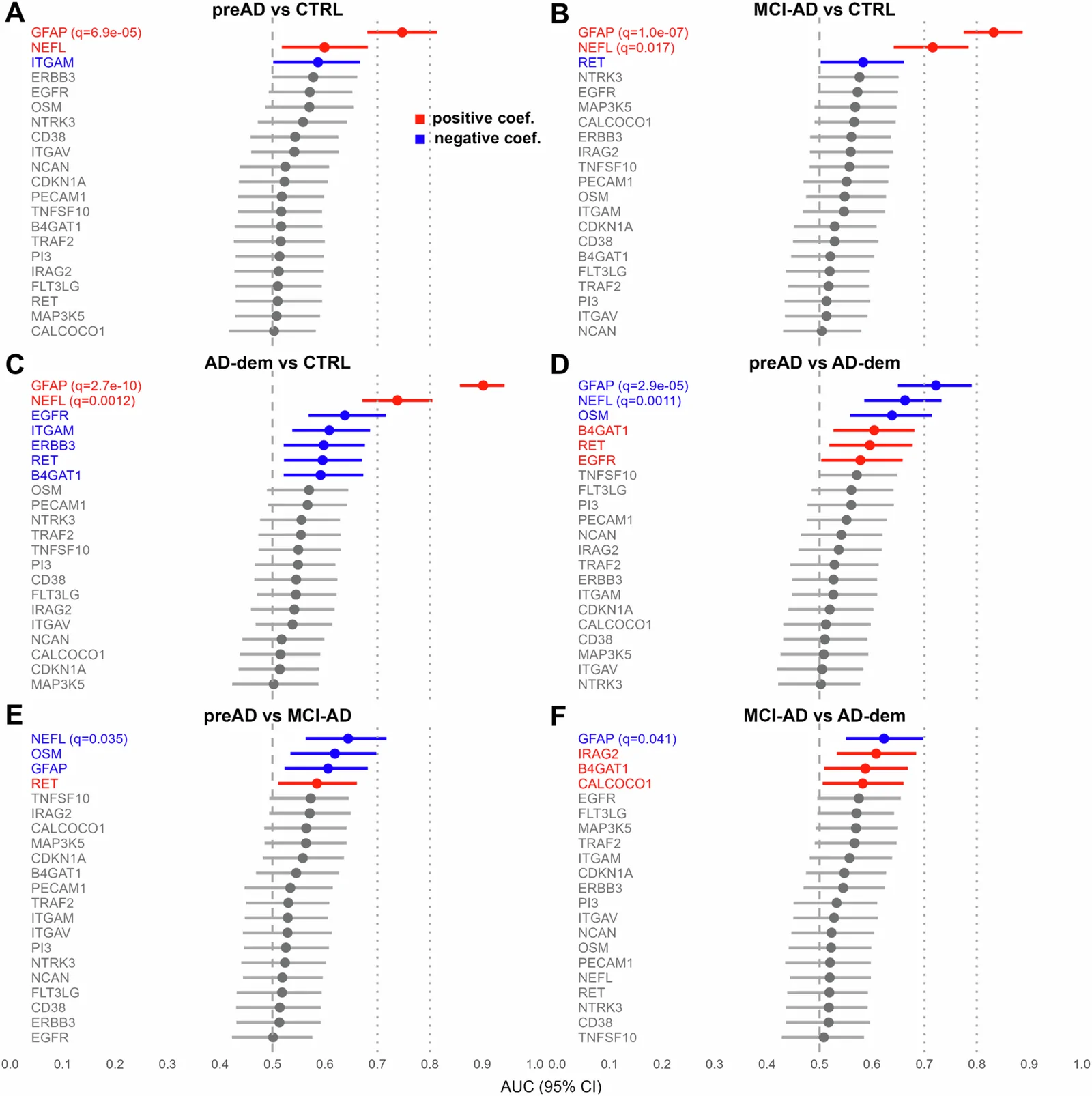
Diagnostic performance of the 21 plasma biomarkers and distribution of selected proteins in differentiating AD from CTRL and among AD stages within the validation cohort. **a–f**) Forest plots showing the area under the curve (AUC) values with 95% confidence intervals for each of the 21 plasma biomarkers measured in the validation cohort. Comparisons include preAD vs CTRL (n=92 vs n=96), MCI-AD vs CTRL (n=109 vs n=96), AD-dem vs CTRL (n=109 vs n=96), preAD vs AD-dem (n=92 vs n=109), preAD vs MCI-AD (n=92 vs n=109), and MCI-AD vs AD-dem (n=109 vs n=109). Each point represents the mean AUC for an individual protein, with horizontal bars indicating 95% confidence intervals estimated by bootstrapping. Q-values next to protein names are p-values adjusted for multiple testing using the Benjamini–Hochberg method (q-values) generated from logistic regression models including age as a covariate. For AUCs greater than 0.5 with 95% confidence intervals entirely above 0.5, red indicates a positive logistic regression coefficient, whereas blue indicates a negative coefficient. Proteins are represented using the names of the genes encoding them. Other abbreviations include AD: Alzheimer’s disease; AD-dem: AD at dementia stage; AUC: area under the ROC curve; CTRL: controls; MCI-AD: mild cognitive impairment due to AD; preAD: preclinical AD. Source data

**Extended Data Fig. 8:**
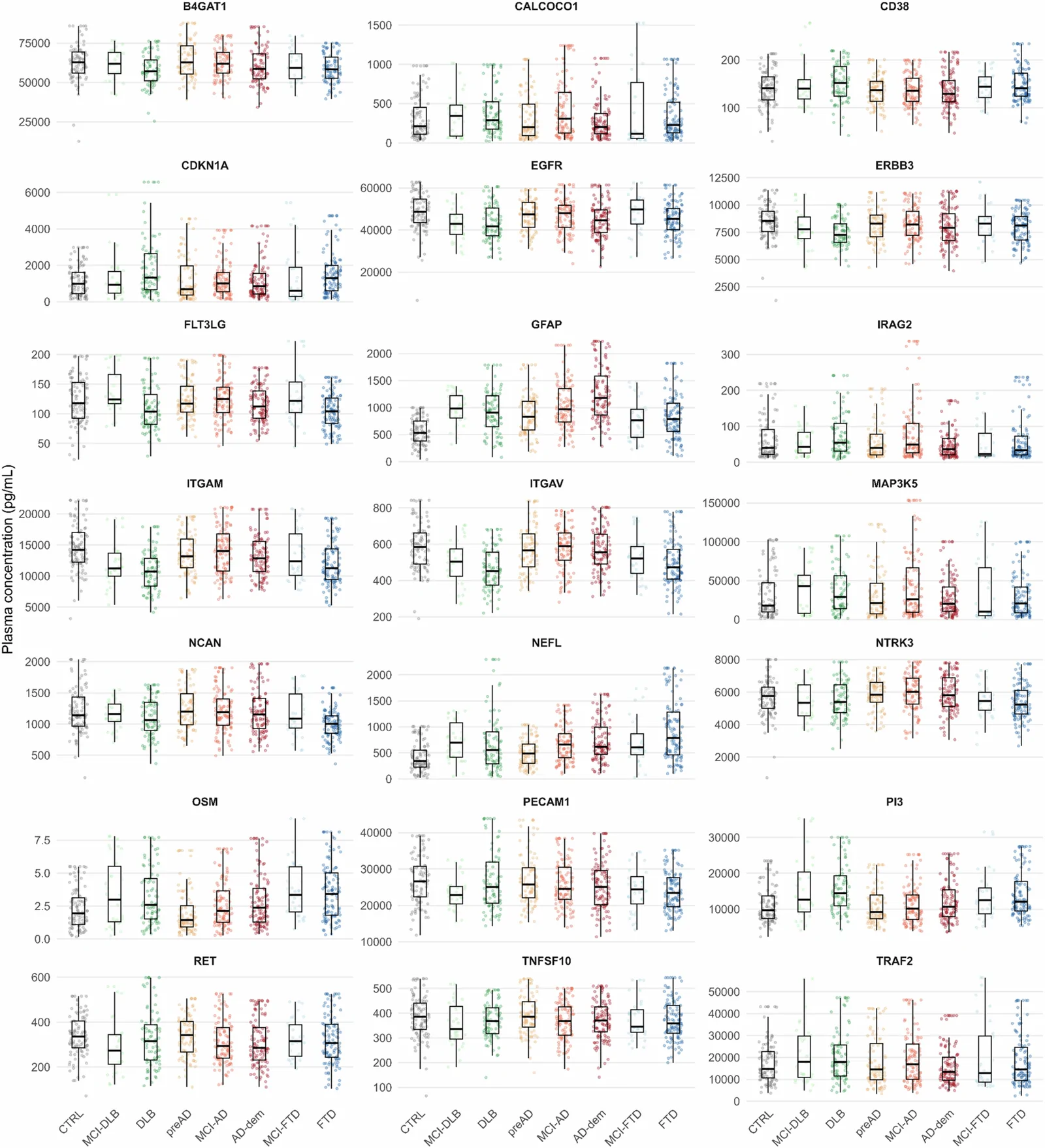
Levels of bPRIDE 21-plex Plasma Dementia Panel proteins in the validation cohort. Boxplots show the distribution of plasma concentrations (pg/mL) for 21 proteins quantified with the custom multiplex panel in the bPRIDE validation cohort (group sizes are reported in Table 1). Each box indicates the interquartile range with the median line. Whiskers reflect the first and third quartiles ±1.5 times the interquartile range. Values were clipped at the 95th percentile within each diagnostic group for visual clarity. Proteins are represented using the names of the genes encoding them. Other abbreviations include AD: Alzheimer’s disease; AD-dem: AD at dementia stage; CTRL: controls; DLB: dementia with Lewy bodies; FTD: frontotemporal dementia; MCI-AD: mild cognitive impairment due to AD; MCI-DLB: mild cognitive impairment associated with DLB; MCI-FTD: mild cognitive impairment associated with FTD; preAD: preclinical AD. Source data

To validate the discovery findings, we developed a custom PEA panel, enabling absolute quantification of the 21 selected proteins. The custom multiplex assay showed optimal coefficients of variation (CVs) (intra-CVs and inter-CVs less than 20% for most of the proteins; Supplementary Table 8). This panel was validated in an independent multicenter cohort (*n* = 722, after precleaning; Table 1). The main DLB candidates, ITGAM and ITGAV, were again reduced in MCI-DLB and DLB (and to a lesser extent in MCI-FTD and FTD) compared to CTRLs and the prodromal and dementia stages of AD (Fig. 6a–d and Extended Data Fig. 8). The main FTD candidates, NfL and OSM, were again elevated in FTD versus CTRL (Fig. 6e–h). Like the discovery cohort, we observed that GFAP was associated with AD across stages (Extended Data Fig. 7) and had the greatest performance in differentiating AD from CTRLs at any clinical stage (AUC of 0.75, 0.83 and 0.9 for pre-AD, MCI-AD and AD-dem versus CTRL, respectively). The distribution of all measured biomarkers across clinical groups is shown in Extended Data Fig. 8.

We next evaluated whether the models developed within the discovery cohort could discriminate the groups of interest in the independent validation cohort measured with the custom 21-panel in absolute quantification. To this end, the protein quantified values measured in the validation cohort were log_2_-transformed and calibrated before model application (Fig. 5). We observed that the models discriminating DLB or FTD from CTRLs could again discriminate such groups with similar performance to those observed in the discovery cohort (AUC of 0.83 and 0.85, respectively; Fig. 5a,c). The models differentiating DLB or FTD from AD had slightly lower, but still fair, performances compared to those of the discovery cohort (AUC 0.78 and 0.77 respectively; Fig. 5b,d). The model for AD staging showed limited performance in the validation cohort, especially in differentiating between pre-AD and MCI-AD (Extended Data Fig. 5).

### Proteins within the plasma biomarker panel correlate with neuropathological hallmarks of AD and DLB in an independent autopsy-confirmed cohort

**Fig. 7:**
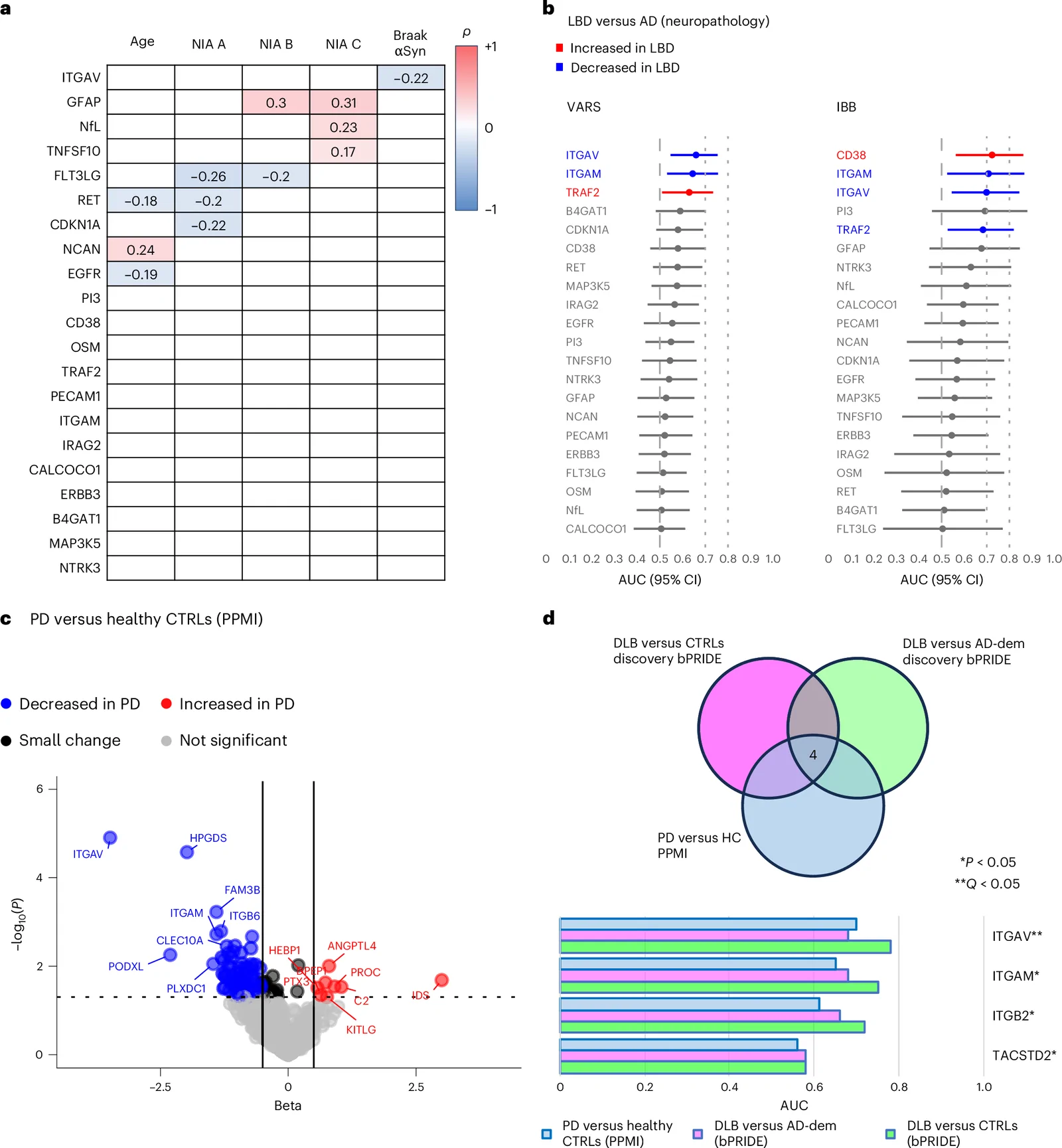
bPRIDE markers in PPMI and neuropathological cohorts. **a**, Spearman’s correlation analysis between bPRIDE markers and neuropathological scores of the VARS cohort (*n* = 129). Only coefficients associated with *P* < 0.05 are displayed. **b**, Forest plots of AUC values with 95% CIs of the bPRIDE proteins for the comparison of patients with LBD pathology and those with AD without consistent LBD pathology from the VARS cohort (*n* = 36 LBD; *n* = 79 AD) and in the IBB cohort (*n* = 8 LBD; *n* = 56 LBD). AUC values greater than 0.5 within their 95% CIs are depicted in red for plasma proteins increased in individuals with LBD and in blue for those that were decreased. **c**, Volcano plot displaying −log_10_ *P* values adjusted for age and sex on the *y* axis and coefficients (Beta) resulting from applying logistic regression on *z*-scored NPX data for PD (*n* = 67) versus healthy CTRLs (*n* = 97) at baseline from the PPMI 9000 study. In the volcano plots, the horizontal line refers to *P* = 0.05; the two vertical lines represent arbitrary thresholds (±0.5) for Beta. **d**, Venn diagram summarizing the proteins consistently decreased in PD and DLB from the PPMI 9000 study and bPRIDE, respectively. For each comparison, the AUC value is also reported together with the statistical significance of *P* and *Q* values (*P* value adjusted for multiple testing). Proteins are represented using the names of the genes that encode them. Source data

To explore potential associations between the proteins of the bPRIDE 21-plex Plasma Dementia Panel and neuropathology scores (that is, Consortium to Establish a Registry for Alzheimer’s Disease and α-synuclein (αSyn) Braak stages), we analyzed the proteins of interest in the PEA Explore data independently generated from antemortem plasma samples from patients of the autopsy-confirmed VARS cohort consisting of AD, DLB and vascular dementia cases. Most patients in this cohort exhibited mixed pathologies and were, on average, 15 years older at the time of plasma collection compared with those in the bPRIDE study (Supplementary Table 1). Despite analytical differences and variations in demographic and clinical features between the VARS and bPRIDE cohorts, we observed that the strongest plasma proteins associated with AD and DLB within the panel correlated with the corresponding classical hallmarks in the neuropathology cohort (Fig. 7a). The proteins associated with AD (that is, FLT3LG, RET and CDKN1A) negatively correlated with amyloid plaque burden (*r* = −0.2 to −0.3). FLT3LG together with GFAP correlated with neurofibrillary tangle pathology (*r* = −0.2 and 0.3, respectively; Fig. 7a). GFAP, NfL and TNFSF10 correlated positively with the degree of neuritic plaques (*r* = 0.31, 0.23 and 0.17, respectively). ITGAV, the protein most associated with DLB, negatively correlated with αSyn Braak stage pathology (Fig. 7a, *r* = −0.22). We next took advantage of the high degree of mixed pathology to gain a better insight into the link between PEA proteins and Lewy body disease (LBD) and investigate which of the 21 selected proteins could be distinguished with an AUC greater than 0.5 within its 95% confidence interval (CI) between AD cases with (Braak αSyn stage > 3) and without (Braak αSyn stage ≤ 3) consistent LBD pathology in the VARS cohort. A similar analysis was conducted on the IBB cohort, included in the independent validation; patients were stratified based on their primary pathology (LBD or AD). In both cohorts, ITGAV and ITGAM emerged as markers associated with LBD (Fig. 7b).

### Plasma DLB biomarkers are also dysregulated in PD

**Extended Data Fig. 9:**
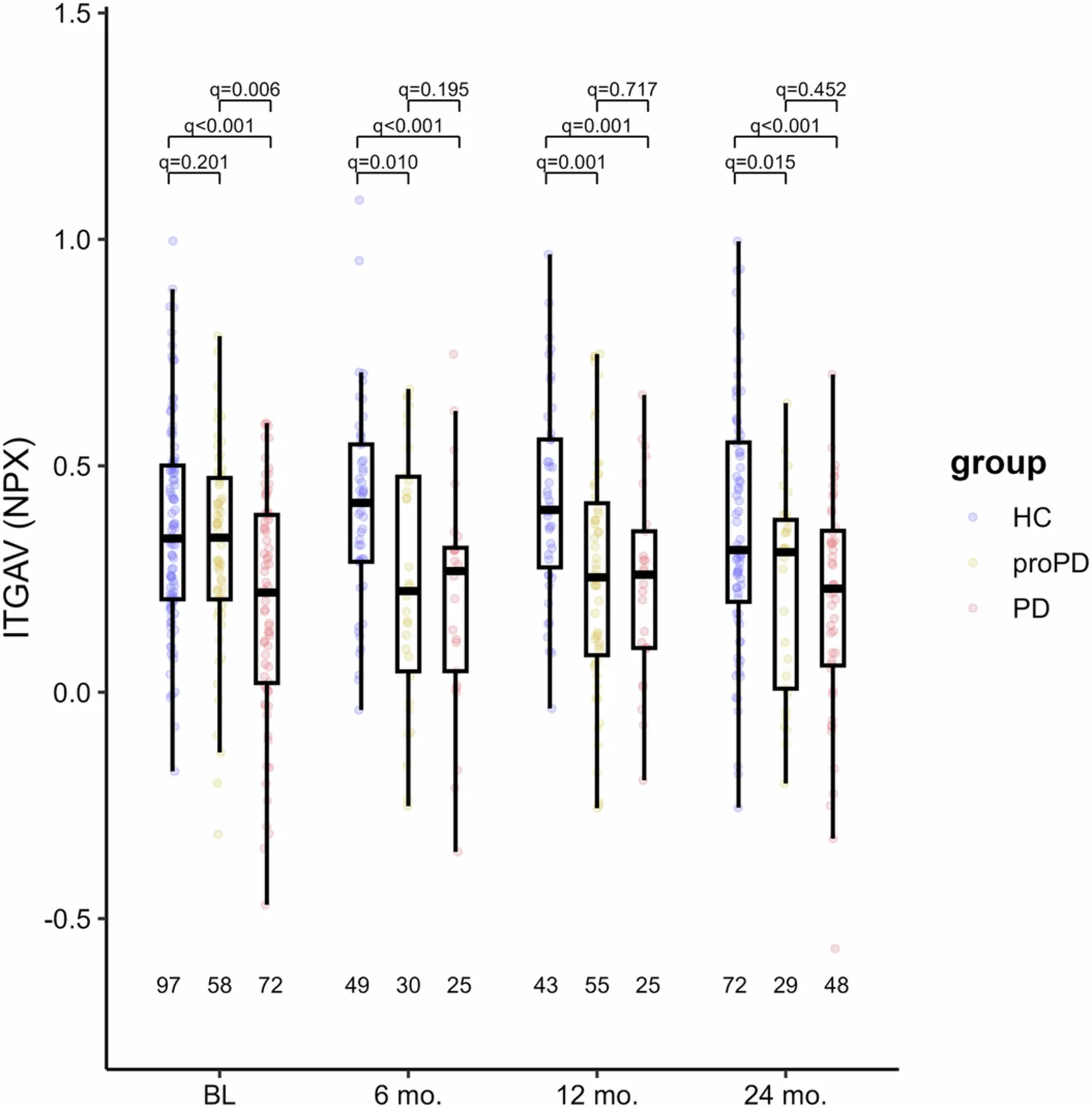
Boxplots displaying the NPX values of ITGAV at different timepoints in PD, proPD, and HC. Boxplots show plasma ITGAV levels (NPX) at baseline (HC n = 97, proPD n = 58, PD n = 72), 6 months (HC n = 49, proPD n = 30, PD n = 25), 12 months (HC n = 43, proPD n = 55, PD n = 25), and 24 months (HC n = 72, proPD n = 29, PD n = 48). Boxes indicate the median and interquartile range, with whiskers extending to 1.5× the interquartile range. Longitudinal trajectories were analyzed using linear mixed-effects models including fixed effects for visit, diagnostic group, their interaction, age, and sex, with a random intercept for participant ID to account for repeated measurements. Pairwise group comparisons within each visit were estimated from marginal means and adjusted for multiple testing using the Benjamini–Hochberg procedure. Abbreviations include HC: healthy controls, PD: Parkinson’s disease, proPD: prodromal PD. Source data

We next analyzed whether the plasma DLB biomarkers identified in this study were also dysregulated in PD, the most common clinical presentation of LBD, using plasma explore PEA data from the PPMI 9000 project, part of which has been recently analyzed^8^. As already observed in DLB and AD with LBD co-pathology, ITGAV and ITGAM were also decreased in PD, with ITGAV exhibiting the lowest *P* value among all proteins measured using the PEA in the PPMI cohort (Fig. 7c,d). HPGDS (hematopoietic prostaglandin D synthase) showed the second most significant effect in PD. In the bPRIDE dataset, HPGDS was also decreased in DLB versus CTRL (Beta = −1.35, *Q* = 0.00002), but its levels were not different between DLB and AD-dem; thus, it was not considered DLB-specific. These two proteins are still significant even after stringent multiple testing correction despite the limited sample size of the PPMI cohort. Analysis of these proteins in the longitudinal PD PPMI cohort, which also include prodromal PD cases (pro-PD), showed that from the 6-month follow-up ITGAV was significantly decreased already in pro-PD compared to healthy CTRLs (Extended Data Fig. 9).

## Discussion

Distinguishing between AD, DLB and FTD remains a clinical challenge, particularly in the early stages or atypical presentations. While FTD can often be distinguished from AD and DLB according to age at onset and symptom profile, overlap still occurs; AD and DLB differentiation is further complicated by frequent mixed pathologies^3,9,10^. Of note, there are no plasma biomarkers for identifying either DLB or FTD. These limitations highlight the need for fluid biomarkers that can complement clinical assessment, improve diagnostics and support patient selection and stratification in research and therapeutic settings. In this cross-sectional multicenter study, we used large-scale PEA proteomics to identify the minimal set of biomarkers for the differential diagnosis of AD, DLB and FTD, and for the molecular staging of AD. We next translated the findings into a custom 21-plex PEA, which was validated in an independent validation cohort. Specific biomarker candidates for DLB were further validated in independent autopsy cohorts.

Unlike observed in previous cerebrospinal fluid (CSF) proteomics studies^11^, most AD-associated plasma proteins were decreased compared to CTRLs, DLB and FTD. This widespread downregulation observed in plasma has also been reported in studies using SomaScan, mass spectrometry and in the recent Plasma Proteome Atlas^12–16^. This generalized reduction may reflect a systemic response to neurodegenerative stress, potentially redirecting resources to the affected organ. It may also result from alterations in protein metabolism or other physiological disturbances in AD. Of note, CALCOCO1, BIN2 and MAP3K5 were among the strongest downregulated proteins specifically associated with AD. CALCOCO1, previously reported as downregulated in AD plasma proteomics^17^, and MAP3K5, which progressively declines across the AD continuum, both regulate endoplasmic reticulum-mediated autophagy^18^, a process associated with AD pathophysiology^19^. Interestingly, BIN2 is involved in phagocytosis, and has been identified as a microglial Aβ-response-associated protein in transgenic murine models of amyloidosis^20^. These findings are in line with the results of the pathway enrichment analysis, which pinpointed to mechanisms primarily related to dysfunctional autophagy in the three AD stages considered.

Among all the plasma proteins that changed along the AD continuum versus CTRLs, GFAP was the one showing the highest discriminative power. This finding aligns with those associating GFAP increase to Aβ-dependent astrogliosis and the onset of tauopathy^21–23^. Still, other studies^21^, including ours using the same bPRIDE samples^2^, have shown that proteins directly reflecting the main AD pathological features (that is, Aβ and Tau pathology) are more sensitive and specific than GFAP in detecting AD. We can thus conclude that none of the proteins detected through PEA proteomics outperforms the plasma biomarkers currently being implemented in routine diagnostics.

Of note, we identified markers that progressively changed across the full AD continuum and might be useful as staging biomarkers for AD. Several efforts have been made to achieve a biological staging of AD using CSF, blood and imaging biomarkers^21,24,25^. Our findings do not only confirm that GFAP and NfL levels increase with disease clinical stage, but also show that other proteins, such as FLT3LG, MAP3K5, PECAM1 and RET decrease with AD clinical progression. We combined these proteins into a composite biomarker score, which exhibited fair and consistent performance in distinguishing pre-AD, MCI-AD and AD-dem. However, such a model did not perform as well in the validation cohort, which might be explained by demographic differences between cohorts or calibration issues. Future studies are needed to validate the performance of this staging model with different measures of clinical and biological disease progression.

Our subsequent analyses were focused on the identification of DLB and FTD and their differential diagnosis with AD. Few plasma proteomic studies in DLB have been performed to date^26–28^; these were limited by sample size or protein coverage. In this study, we analyzed more than 1,000 proteins in up to 167 cases with DLB in the discovery cohort and further tested the 21 selected proteins in 81 cases with DLB. We observed that from the 71 dysregulated proteins in DLB compared to CTRLs, only 19 were also differentially regulated between DLB and AD, including ITGAV, ITGAM, PI3 and ERBB3. Among them, ITGAV and ITGAM showed decreased levels in DLB independent of Aβ biomarker positivity. These two proteins were also to some extent decreased in FTD, although with lower statistical power. ITGAV, ITGAM and ERBB3 were also decreased in the cases with DLB and MCI-DLB included in the independent validation cohort measured with our 21-plex custom panel, in patients with PD in the PPMI cohort, and in cases with LBD pathology in the autopsy-confirmed VARS and IBB cohorts. An independent study conducted in parallel to ours also reported downregulation of ITGAV, ITGAM and ERBB3 in PD^29^. These results underpin the strong potential of these proteins as the first plasma biomarkers for LBD. Integrins mediate cell-to-cell and cell-to-matrix interactions, with known roles in immune regulation, synaptic remodeling and blood–brain barrier integrity. However, their involvement in neurodegenerative diseases remains largely unknown. Of note, ITGAV forms a functional signaling complex with ERBB3 (ref. ^30^). This integrin–ERBB3 complex is activated by neuregulin 1 (NRG1), a trophic factor essential for neuronal survival, axonal guidance and synaptic plasticity. Notably, NRG1 levels have previously been reported to be reduced in PD^31^; NRG1 has been investigated as a pharmacological target for restoring dopaminergic function^32^. Therefore, the coordinated downregulation of ITGAV and ERBB3 may reflect impaired NRG1-mediated neurotrophic signaling in LBD, potentially contributing to synaptic dysfunction or reduced neuronal resilience. In line with this, ITGAV has been shown to mediate dopaminergic neurite outgrowth and connectivity in an experimental model^33^, suggesting that integrin dysregulation could represent a downstream consequence of dopaminergic pathway impairment, a key dysfunctional pathway in DLB and PD. The modest reduction of these integrins in plasma from patients with FTD further supports this hypothesis, in line with evidence of dopaminergic dysfunction in approximately one-third of cases with FTD^34^ and the CSF alterations in dopaminergic dysfunction markers observed also in FTD^35^.

These integrins could distinguish DLB from both CTRLs and AD-dem, although their individual performance was modest (AUC between 0.70 and 0.80). Thus, we applied multivariate classification modeling to identify the smallest combination of markers providing optimal discrimination for DLB. This approach yielded compact panels of five and seven proteins, including ERBB3, ITGAV and ITGAM, which together achieved good performance in distinguishing DLB from AD-dem and CTRLs. The models showed consistent accuracy across training and test sets in the discovery cohort (AUC ~ 0.85–86) and retained comparable performance in the independent validation cohort (AUC ~ 0.78–0.83), despite expected variability from assay calibration differences between the high-throughput and custom PEA platforms (Fig. 5).

In the case of FTD, we identified 23 proteins that were dysregulated compared to both AD-dem and CTRLs in the discovery cohort. NfL showed the greatest effect size compared to CTRLs, while GFAP had the most significant effect compared with AD-dem. The LASSO routine identified a signature with 10 and 2 proteins that could discriminate FTD from CTRLs and AD-dem well, respectively, in both the discovery and independent validation cohorts (Fig. 5). The strongest contributors to these two models were GFAP and NfL. Considering that NfL is not an FTD-specific biomarker and has limited performance to discriminate FTD from AD-dem^36,37^, GFAP is probably the biomarker driving the strongest effect to discriminate these groups. In line with previous studies^38^, we observed that GFAP was elevated in FTD compared to CTRLs, but to a considerable lesser extent than in AD, suggesting a different or reduced astrocytic response in FTD compared to AD. Other studies have shown increased levels of plasma GFAP only in cases with FTD with a *GRN* mutation^39^. Differences in plasma GFAP levels have also been recently observed across FTD pathological subtypes^40^, further indicating that the levels of plasma GFAP vary across genetic and pathological FTD subtypes. We also observed that OSM and MMP9 were increased in MCI-FTD compared with both CTRLs and MCI-AD, the former further validated in the validation cohort. Although MMP9 could not be included in the custom assay because of technical incompatibilities, similar changes were also observed in our recent CSF proteomic study of FTD^35^. OSM and MMP9, which have been previously associated with the inflammatory response^41^ and brain–blood barrier disruption^42^, respectively, are promising plasma biomarker candidates specifically associated with FTD that may probe earlier pathogenic events in FTD and NfL changes. Future studies are needed to understand the expression of these proteins along the complex spectrum of FTD subtypes.

This study is not without limitations: (1) the DLB and FTD diagnoses were based solely on clinical evaluation, which does not exclude the possibility of misdiagnosis. However, samples came from clinical memory units specialized in the evaluation of such disorders that, together with the large sample size used, minimize the impact of potential misdiagnosis; (2) we cannot exclude the presence of αSyn co-pathology in patients with AD, which is particularly common^3^, especially at the dementia stage^9,10^; (3) despite promising FTD candidate biomarkers having been identified, the group with FTD included a large spectrum with different genetic and pathological subtypes, thus introducing heterogeneity, which may have hampered the identification of a larger number of FTD plasma biomarkers; (4) we could not identify a robust plasma biomarker diagnostic model for cases with pro-DLB and FTD, which is probably explained by the relatively low sample size in the discovery cohort (*n* = 25 and *n* = 45, respectively) and limited phenotyping of these groups. Still, promising single biomarker leads were detected (for example, ITGAV and ITGAM for LBD and OSM for FTD); and (5) the results obtained are to some extent platform-dependent, and additional proteins might be identified with other alternative and complementary approaches. Despite these limitations, we consistently detected key AD-associated, DLB-associated and FTD-associated plasma proteins across two large multicenter cohorts and in autopsy-confirmed cases, supporting the robustness of our findings. In summary, this cross-sectional multicenter plasma proteome study identifies disease-specific plasma proteins and establishes a custom quantitative multiplex assay enabling biomarker-based differential diagnosis across the main causes of dementia.

## Methods

### Ethics statement

All procedures involving human participants were conducted in accordance with the 2013 revision of the Declaration of Helsinki and the ethical standards of the relevant institutional and national research committees. All participants provided written informed consent for the use of clinical data and biomaterials for research purposes. The study was approved by the relevant local ethics committees at participating centers, including the Comitato Etico Aziende Sanitarie Regione Umbria (19369/AV and 20942/21/OV), the Amsterdam UMC VUmc Medical Ethics Committee (2019.102), the Sant Pau Ethics Committee cohort, the Ethics Committees of Motol University Hospital and St. Anne’s University Hospital (CBAS) cohort and the relevant ethics and biobank governance bodies overseeing the IBB/BIODEM cohort. Local health authorities approved the VARS patient follow-up program at the Queen Sofia Alzheimer’s Center (ref. no. MCB/RMSFC, record no. 12672).

### Participants and inclusion criteria

The total discovery cohort (1,318 plasma samples initially included in this project) were collected from individuals enrolled across six different international cohorts: Amsterdam Dementia Cohort (ADC) (the Netherlands; *n* = 221; all groups^43^), Sant Pau Initiative on Neurodegeneration (SPIN) (Spain, *n* = 220; all groups^44^), University of Perugia (UNIPG) (Italy, *n* = 167; all groups^21^), Ulm University (UU) (Germany, *n* = 174; all groups except MCI-DLB^45^), BioFINDER (Sweden, *n* = 383; CTRLs and complete AD-cont^46^) and AIBL (Australia, *n* = 153; controls and complete AD continuum^47^). An independent large multicenter cohort similar to the discovery one from ADC (*n* = 179; all groups), SPIN (*n* = 137; all groups), UNIPG (*n* = 119; all groups), UU (*n* = 38, controls and FTD), AIBL (*n* = 104; controls and complete AD continuum) and CBAS^48^ (*n* = 159; all groups) together with an autopsy-confirmed AD/DLB cohort coming from BIODEM and the neurobiobank of the IBB/UAntwerp^49^ (total *n* = 69; AD and DLB) was used to validate the custom panels.

For all the samples, the presence of AD pathology was tested either by using AD CSF biomarkers (CSF Aβ_42_ or Aβ_42/40_, Tau phosphorylated at threonine 181 (pTau_181_) and total Tau (t-Tau), all cohorts) or amyloid and Tau-PET (most patients from the AIBL cohort). The clinical groups included were cognitively unimpaired controls (CTRLs) without biomarker evidence of Aβ pathology (*n*_discovery_ = 374; *n*_validation_ = 108); cognitively unimpaired individuals with Aβ pathology (pre-AD; *n*_discovery_ = 186; *n*_validation_ = 108); individuals with MCI because of AD (MCI-AD; *n*_discovery_ = 164; *n*_validation_ = 116, with positive AD biomarkers); individuals with MCI because of FTD (MCI-FTD; *n*_discovery_ = 46; *n*_validation_ = 34); individuals with MCI because of DLB (MCI-DLB; *n*_discovery_ = 25; *n*_validation_ = 36); individuals with AD-dem (*n*_discovery_ = 182; *n*_validation_ = 171, with positive AD biomarkers); individuals with FTD (*n*_discovery_ = 170; *n*_validation_ = 126); and individuals with DLB (*n*_discovery_ = 171; *n*_validation_ = 106). In the discovery cohort, two patients with FTD (with *MAPT* and *C9ORF72* mutations) and 12 with DLB had missing Aβ status. In the validation cohort, five CTRLs and 33 individuals with FTD from UU, together with two individuals with DLB and four with MCI-DLB from the CBAS cohort, had missing Aβ status. CTRLs included individuals with subjective cognitive decline, in whom objective cognitive and laboratory investigations were normal (that is, criteria for MCI, dementia or any other neurological or psychiatric disorder not fulfilled) with negative AD biomarkers (except from the few exceptions within the validation autopsy cohort mentioned above)^43,50^. All participants of each cohort underwent standard neurological and cognitive assessments; the diagnosis was assigned according to international consensus criteria for MCI-AD^51^, AD-dem^52^, DLB^53^ and FTD^54,55^. Cases with DLB in the discovery cohort with available Aβ biomarkers were further split for Aβ co-pathology (*n* = 86 Aβ^+^, *n* = 70 Aβ^−^). The MCI-DLB and MCI-FTD discovery groups contained a very small number of participants with regard to the number of measurements and were considered just for basic univariate analyses. Plasma samples were collected according to standardized international guidelines^56^. The only two reported preanalytical differences across centers were the addition of prostaglandin E1 to EDTA tubes collected by AIBL and the use of citrate plasma for VARS.

The MMSE or the Montreal Cognitive Assessment test were used as a measure of global cognition. CSF markers were analyzed locally as part of the diagnostic procedure using commercially available kits (ADC, AIBL and UU: ELISA INNOTEST, Fujirebio; ADC: Elecsys biomarker assays, Roche Diagnostics; SPIN, UNIPG and UU: Lumipulse G600, Fujirebio; BioFINDER: Euroimmun ELISA assays). All samples from the AIBL cohort were defined based on Aβ and Tau-PET. A positive CSF AD biomarker profile was defined locally in the different cohorts as follows: in ADC as Aβ^+^ (INNOTEST Aβ_42_ < 813 pg ml^−1^; Elecsys Aβ_42_ < 1,000 pg ml^−1^) and Tau^+^ (INNOTEST p-Tau_181_ > 52 pg ml^−1^; Elecsys p-Tau_181_ > 24 pg ml^−1^); in BioFINDER as Aβ_42_/Aβ_40_ < 0.088 and either p-Tau_181_ > 65.04 pg ml^−1^ or t-Tau greater than 462.91 pg ml^−1^; in SPIN as Aβ_42_/Aβ_40_ < 0.062 and either t-Tau greater than 456 pg ml^−1^ or p-Tau_181_ > 63 pg ml^−1^; in UNIPG as Aβ_42_/Aβ_40_ < 0.072 and p-Tau_181_ > 50 pg ml^−1^ (regardless of t-Tau); in UU as Aβ_42_ < 450 pg ml^−1^ and either t-Tau greater than 380 pg ml^−1^ or p-Tau greater than 65 pg ml^−1^; in CBAS as either CSF biomarkers (Aβ_42_ < 526 pg ml^−1^ and p-Tau_181_ > 50.2 pg ml^−1^) or amyloid PET positivity; and in AIBL based on amyloid and Tau-PET (amyloid PET^+^ greater than 25 centiloids; Tau-PET^+^ meta-temporal standardized uptake value ratio greater than 1.19). The methodology and cutoff values used for each cohort and each biomarker are summarized in Supplementary Table 7. In addition, for the discovery cohort, anamnestic data on six common systemic comorbidities (dyslipidemia, diabetes, hypertension, hypothyroidism, chronic kidney disease and obesity with a body mass index greater than 30), were collected for most participants (*n* = 823 for most comorbidities; *n* = 418 for obesity). These data were used to assess potential confounding effects on plasma protein levels. The autopsy IBB cohort analyzed in the validation of the custom assay included cases with a definite diagnosis according to international neuropathological examination guidelines for DLB^53,57^ and AD^58^. Within the autopsy-defined DLB group (*n* = 10), coexisting AD pathological changes were reported in five cases. Coexisting cerebrovascular lesions (*n* = 1), TDP-43 pathology (*n* = 1) were reported within the autopsy-confirmed AD neuropathological group (*n* = 60).

Independent data (which include the same 384 PEA panels: cardiometabolic, inflammation, neurology and oncology) generated from the plasma samples collected from patients at the dementia stage with autopsy confirmation of AD, vascular dementia and LBD pathology in the VARS cohort^59,60^ (*n* = 129) were used to explore associations between the selected biomarkers and neuropathological scores (that is, Aβ, Tau and αSyn pathology). The composite neuropathological score used to characterize the VARS cohort was the ABC method^58^ recommended by the National Institute on Aging (NIA) and defined as NIA A, NIA B and NIA C to refer to (A) diffuse Aβ plaque burden (based on Thal phases; 0 = 0, 1 = 1/2, 2 = 3, 3 = 4/5); (B) neurofibrillary tangle burden (based on Braak stages; 0 = none, 1 = I/II, 2 = III/IV, 3 = V/VI); and (C) neuritic plaque location and density (based on the Consortium to Establish a Registry for Alzheimer’s Disease score; 0 = none, 1 = sparse, 2 = moderate, 3 = frequent). Each of the three components is scored 0–3. αSyn pathology was characterized using the Braak αSyn stages^61^, which are scored from 1 to 6, 1 being disease onset in the lower brainstem and 6 the most severe stage of the disease affecting the neocortex. A summary of these neuropathological scores in the VARS cohort, along with demographic details, is presented in Supplementary Table 1.

The PEA data generated on 221 individuals from the PPMI^62^ 9000 study (projects 196 and 222) were also included to test whether some of the proteins associated to DLB could be associated to other neuronal αSyn diseases^63^. This cohort included patients with PD (*n* = 73), healthy CTRLs (*n* = 97), individuals with pro-PD and individuals with symptoms or genetic mutations suggestive of PD without objective motor deficits (*n* = 58). All PD and healthy CTRLs were drug-naive at the time of blood sampling; patients labeled as PD but with neuronal αSyn disease staging of 0 (5 of 73) were excluded from the analysis. Data used in the preparation of this article were obtained on 31 August 2024 from the PPMI database (www.ppmi-info.org/access-data-specimens/download-data), research resource identifier: SCR_006431. For up-to-date information on the study, visit www.ppmi-info.org. Data from samples collected at baseline, 6 months, 12 months and 24 months were used for the analysis. The baseline clinical and demographic details of the included individuals are summarized in Supplementary Table 2.

### Plasma protein profiling

Proteomic measurements were performed on randomized sample plates according to standardized assay procedures, with laboratory personnel blinded to diagnostic group allocation. In the discovery cohort, a total of 1,536 plasma proteins were quantified using the four specific and validated multiplex-antibody-based protein panels based on the PEA, which were available when the analysis was performed (Explore 384 Cardiometabolic, Explore 384 Inflammation, Explore 384 Neurology, Explore 384 Oncology, Olink Proteomics). Briefly, samples were randomized across 18 plates containing appropriate intra-plate and inter-plate quality controls (QCs) from the manufacturer. Intra-plate and inter-plate CVs for all panels are reported in Supplementary Table 9, with average CVs being below 20%. Each assay has an experimentally determined lower limit of detection (LLOD) estimated as three standard deviations above the noise level from the negative controls that are included on every plate. As QC measures at the individual protein level, we excluded from the analysis protein duplicates, those proteins with more than 15% values below the LLOD and those with more than 15% values associated with QC warnings. As QC measures at the sample level, samples producing more than 35% QC warnings were removed from the subsequent analyses because this typically indicates broader technical issues or suboptimal sample quality. For the included proteins, NPX values below the LLOD were not imputed or substituted because they represent only 0.74% of the total data and we considered the measured values to represent the best available estimates. Following these procedures, 520 proteins and 41 samples were removed (seven AD-dem, 12 CTRLs, four DLB, seven FTD, six MCI-AD, one MCI-FTD, four pre-AD), resulting in 1,016 unique proteins measured in 1,277 individuals. Sensitivity analysis showed that none of the excluded proteins offered better discriminatory value than those retained in the biomarker panel.

### Development of custom PEA

A custom-designed multiplex PEA was developed by the manufacturer, according to standardized protocols^64,65^, to quantify the 21 proteins selected in the discovery cohort. The custom assay was then tested in an independent validation cohort (Table 1). Besides clinical samples, each plate included three replicates of four plasma QC samples (two plasma pools made from patients with AD and two plasma pools made from CTRLs) and three calibrators used for normalization. QC samples and calibrators were measured in triplicate. Each custom assay has an experimentally determined LLOD defined as for the discovery panels. Precision (intra-assay and inter-assay CVs) were calculated using the four QC samples. No cross-reactivity between assays for specific proteins was detected. Assay parameters including LLOD and CVs are included in Supplementary Table 8. Samples from the validation cohorts (*n* = 805) were randomized across plates and normalized for any plate effect using the built-in inter-plate controls according to the manufacturers’ recommendations, with laboratory personnel blinded to diagnostic group allocation. Protein abundance was reported both in NPX and in absolute quantification (pg ml^−1^). The same QC measures described above for the discovery cohort were applied within the validation cohort using the custom PEA assay. A total of 83 samples (*n* = 13 CTRLs, *n* = 16 pre-AD, *n* = 6 MCI-AD, *n* = 16 AD-dem, *n* = 6 MCI-DLB, *n* = 2 MCI-FTD, *n* = 15 DLB, *n* = 9 FTD) were not included in the analysis because of QC warnings present in more than 19 of the 21 proteins measured within the panel (*n* = 62) because of sample QC warnings (*n* = 20) or both (*n* = 1).

### Statistics and reproducibility

Statistical analyses were performed in Python v.3.0, R v.4.2.3 and OriginLab 9. All hypothesis tests were two-sided unless otherwise specified. Sample sizes were determined based on the availability of well-characterized participants across cohorts, with a minimum target of more than 25 individuals per group in the discovery phase. In the validation phase, sample sizes were further expanded to enable the assessment of effects in prodromal groups. No randomization was used to allocate participants to clinical groups, as diagnostic categories were defined according to clinical and biomarker criteria. Differences in demographic variables among groups were assessed using Dunn’s test for continuous variables (that is, age and MMSE) and Fisher’s exact test for categorical variables (that is, sex). Principal component analysis coupled to analysis of variance was used to assess center effects on scaled proteomic data. Logistic regression was applied to analyze the differential protein expression for the pairwise comparison of clinical groups using *z*-scored NPX or absolute quantification data, adding age or sex as covariates and adjusting *P* values for multiple testing with Benjamini–Hochberg correction^66^. The corrected *P* values have been referred as *Q* values. Along with *Q* values and Beta coefficients, Cohen’s *d* and AUC ROC were also computed for each protein and pairwise comparison. AUC values were averaged between comparisons to rank proteins for their association with AD, DLB and FTD. A similar approach was used to identify promising staging biomarkers for AD by averaging −log_10_ of *Q* values and Beta coefficients resulting from logistic regression applied for pre-AD versus MCI-AD, MCI-AD versus AD-dem and pre-AD versus AD-dem comparisons. In the plots, a nonparametric Theil–Sen regression line fitting the data from pre-AD to AD-dem was also added for visualization purposes.

Pathway enrichment analysis was performed using the effect sizes (Beta) and *P* values obtained from the protein comparison between each diagnostic group (pre-AD, MCI-AD, AD-dem, DLB and FTD) to the CTRL group. We used the R package KEGGREST for the analyses, calculating the difference (Wilcoxon signed-rank test) between the *P* values in each pathway compared to the background (all other measured pathways). A threshold was set at a minimum count of three proteins measured within the pathway. Otherwise, the pathway was excluded. The pathway effect size is defined as the median Beta of all measured proteins within the pathway. The obtained *P* values for the pathways were FDR-corrected. The main classes are ranked according to the number of pathways associated with the largest class at the top. Subclasses within each main class were similarly ranked based on the number of pathways, with individual pathways displayed as the final hierarchical level.

For the 21-protein panel selection, we performed multivariable modeling analysis, in which the bPRIDE discovery cohort was randomly split into training (70% of the data) and test (remaining 30%) sets. Each comparison was randomized for each clinical group to keep group prevalences consistent in the training and test sets. For each of the DLB versus CTRLs, DLB versus AD-dem, FTD versus CTRL and FTD versus AD-dem comparisons, 100-fold cross-validated LASSO binomial regression was applied in the training set to analyze the selection proportion of proteins in models by considering a fixed model size of five. The glmnet^67^ R package was used for this purpose. For the molecular staging of AD, candidates for a composite biomarker were identified using Gaussian LASSO regression, including the clinical stages of AD as numeric variables (pre-AD = 1, MCI-AD = 2, AD-dem = 3). To make the training of this staging composite biomarker as robust as possible, we first filtered the training set by removing participants with MMSE values outside the IQR of each AD clinical stage (*n* = 82 individuals removed on *n* = 361 within the training set). The results of the selection proportion analysis, for each comparison, were used to select 21 proteins for a dementia-oriented PEA panel (21 is the maximum allowed size for the physical realization of a custom PEA panel). To evaluate the potential confounding impact of systemic comorbidities (that is, dyslipidemia, diabetes, hypertension, hypothyroidism, chronic kidney disease and obesity) on protein levels, we performed a nonparametric two-way analysis of variance for each protein, using comorbidity status (present/absent) and clinical group as independent variables.

We next applied binomial LASSO regression to train models within the 21-marker panel for the different group comparisons (that is, DLB versus AD-dem, DLB versus CTRLs, FTD versus AD-dem and FTD versus CTRL) and tested their performance on the remaining 30% of the discovery cohort (test set). The internal cross-validation algorithm within the glmnet R package was used to determine the optimal model size based on the penalization parameter at one standard error from the maximum AUC. Subsequently, for the composite biomarker for the molecular staging of AD, we applied Gaussian LASSO, as described previously.

The developed models were validated in the independent validation cohort analyzed with the custom 21-biomarker panel developed within the study. To this purpose, the log_2_ of absolute quantification data was considered. To account for the different nature of the assay used on the validation cohort, biomarker values from the new dataset were recalibrated against the distribution observed in the discovery cohort. For each analyte, we derived scale parameters (median and IQR) from the discovery CTRL group. For the validation data, log_2_-transformed values (*Q*′) were transformed into standardized *z*-scores using the median and IQR, and then rescaled to match the reference distribution:

$${Q}^{{\prime} }={\log }_{2}Q$$

$${Q}^{{\prime} {\prime} }=\frac{{Q}^{{\prime} }-\mathrm{median}\left({Q}_{{\mathrm{validation}}}^{{\prime} }\right)+\mathrm{median}\left({{\mathrm{NPX}}}_{{\mathrm{CTRL}}_{\mathrm{discovery}}}\right)}{{\mathrm{IQR}}\left({Q}_{\mathrm{validation}}^{{\prime} }\right)}{\mathrm{IQR}}\left({Q}_{{\mathrm{CTRL}}_{\mathrm{discovery}}}\right)$$

The calibration coefficients are present in Supplementary Table 10.

Each model’s performance was then evaluated using ROC analysis. For the FTD versus CTRL comparison, *n* = 38 missing values of B4GAT1 were imputed using mean *Q*″ values in the CTRL and FTD groups. The 95% CIs of the AUCs were calculated by using 2,000 bootstrap replicates in the training and test sets using the R package pROC^68^. Spearman’s correlation analysis was applied to explore possible associations between the 21 bPRIDE biomarkers and neuropathological scores of the VARS cohort. Longitudinal changes in plasma ITGAV levels within PD, pro-PD and healthy CTRL participants from the PPMI cohort were assessed using linear mixed-effects models, including age and sex as covariates and participant ID as a random intercept. Post-hoc within-group comparisons across visits were adjusted using the Benjamini–Hochberg procedure.

### Reporting summary

Further information on research design is available in the Nature Portfolio Reporting Summary linked to this article.

## Supplementary information

Supplementary Information Supplementary Figs. 1 and 2 and Tables 1–10.

Reporting Summary

Peer Review File

Supplementary Data 1 Source data for Supplementary Figs. 1 and 2.

## Source data

Source Data Fig. 2 Statistical source data for discovery AD analyses, including differential protein expression results across AD stages versus controls and AD-dem versus DLB/FTD, with beta coefficients, p-values, q-values, Cohen’s d and AUC values.

Source Data Fig. 3 Statistical source data for AD molecular staging analyses, including individual biomarker values across preAD, MCI-AD and AD-dem, pairwise comparison statistics, and trend/regression source data for selected staging markers.

Source Data Fig. 4 Statistical source data for discovery DLB and FTD analyses, including differential protein expression results for DLB and FTD versus CTRL and AD-dem, with beta coefficients, p-values, q-values, Cohen’s d and AUC values.

Source Data Fig. 5 Statistical source data for multivariable diagnostic models, including LASSO model coefficients, predicted scores/probabilities, ROC source data and AUC statistics for DLB and FTD versus CTRL and AD-dem in training, test and validation sets.

Source Data Fig. 6 Validation cohort source data for selected DLB- and FTD-associated biomarkers, including individual plasma concentrations and statistical results for ITGAV, ITGAM, NEFL and OSM across diagnostic groups.

Source Data Fig. 7 Statistical source data for independent neuropathology and Parkinson’s disease validation analyses, including the Queen Sofia Foundation’s Vallecas Alzheimer Project (VARS) neuropathological correlation results, LBD classification AUCs, PPMI differential protein statistics and longitudinal ITGAV model outputs.

Source Data Extended Data Fig. 1 Statistical source data for KEGG pathway enrichment and BRITE hierarchy analyses in the AD continuum, including pathway annotations, measured protein counts, median beta effects, p-values and FDR-adjusted p-values.

Source Data Extended Data Fig. 2 Statistical source data for DLB analyses stratified by amyloid biomarker status, including differential expression results for DLB Aβ+ and DLB Aβ− versus CTRL and AD-dem, with beta coefficients, p-values, q-values and Cohen’s d values.

Source Data Extended Data Fig. 3 Statistical source data for MCI-DLB and MCI-FTD analyses, including differential protein expression results and biomarker performance metrics versus CTRL and MCI-AD.

Source Data Extended Data Fig. 4 Protein selection frequencies from cross-validated LASSO regression used to prioritize proteins for the 21-plex plasma dementia biomarker panel.

Source Data Extended Data Fig. 5 Statistical source data for the AD molecular staging composite model, including model scores in training, test and validation sets, ROC curve source data, AUC statistics and model coefficients.

Source Data Extended Data Fig. 6 Statistical source data evaluating the impact of systemic comorbidities on the 21 selected proteins, including available sample counts and two-way ANOVA results for comorbidity and clinical group effects.

Source Data Extended Data Fig. 7 Validation cohort source data for AD diagnostic performance analyses, including AUC values, 95% confidence intervals, p-values, q-values and plasma concentration data for AD-stage comparisons.

Source Data Extended Data Fig. 8 Individual plasma concentrations (pg/mL) for all 21 proteins quantified with the custom multiplex panel in the bPRIDE validation cohort, underlying the validation cohort boxplots.

Source Data Extended Data Fig. 9 Longitudinal PPMI source data and statistical results for plasma ITGAV across HC, prodromal PD and PD participants, including visit-level biomarker measurements and mixed-effects model outputs.

## Data Availability

The source data underlying all figures in the main text, Extended Data, and Supplementary Information are provided with this paper. The proteomics datasets supporting the findings of this study have been deposited in Synapse and are publicly available at https://www.synapse.org/Synapse:syn75233598. Source data are provided with this paper.

## Code availability

Custom scripts were used for data preprocessing, QC, statistical analyses, model implementation, calibration and figure generation, based on established and publicly available methods described in Methods. No new software or proprietary computational algorithm central to the study conclusions was developed.
