# Supplementary material for: Plasma proteome profiling identified biomarkers for the differential diagnosis and molecular staging of neurodegenerative dementias
Source: Nat Aging. 2026 Jul 20;6(8):1717–32. doi: 10.1038/s43587-026-01162-7 (PMC13472907; doi:10.1038/s43587-026-01162-7)
Supplement: Supplementary file 1 [file 43587_2026_1162_MOESM1_ESM.pdf]

nature aging

Supplementary information https://doi.org/10.1038/s43587-026-01162-7

Plasma proteome profiling identified biomarkers for the differential diagnosis and molecular staging of neurodegenerative dementias

In the format provided by the authors and unedited

Supplementary material

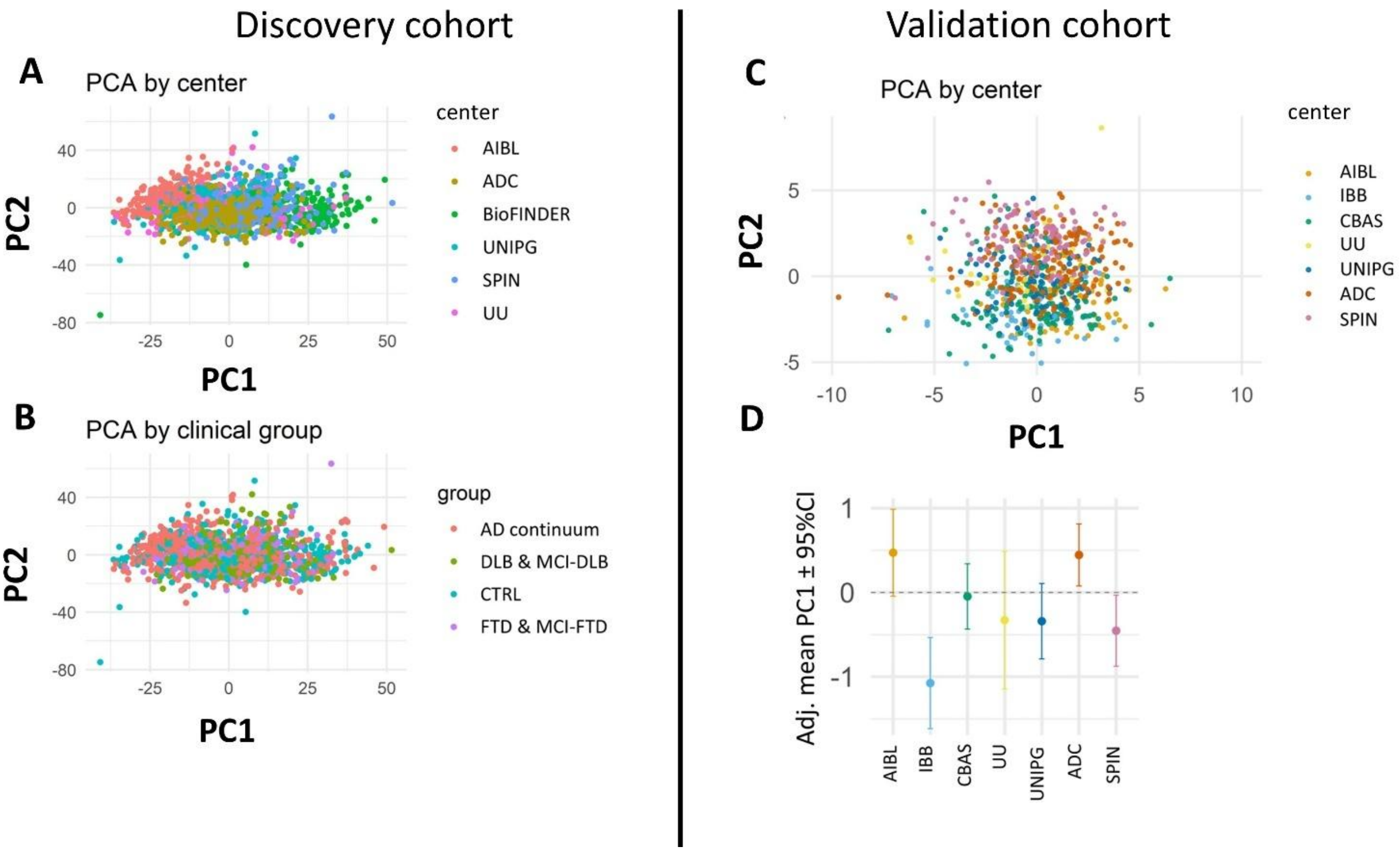

Supplementary Figure 1. Principal component analysis (PCA) of the included samples and proteins. The samples in the PCA plot for the discovery cohort were colored according to A) center and B) clinical group. Center was found significantly associated with the first principal component (PC1, $p<0.001$) by one-way analysis of variance. This effect was, at least partially, motivated by group imbalance among centers, e.g., most subjects from AIBL belonged to the AD continuum group, while most CTRL belonged to BioFINDER. C) Samples in the PCA plot for the validation cohort were colored according to center. Clinical groups were more balanced in the validation cohort and did not produce any visible or significant variation in the PCA plot (not shown). D) Adjusted mean ±95% confidence intervals of PCA component 1 (PC1) across cohorts, estimated from linear models including clinical group as covariate. Centers with non-overlapping CIs or large deviations from zero indicate potential systematic cohort effects (e.g., IBB autopsy-confirmed cohort). Abbreviations used: AD, Alzheimer's disease; CTRL, controls; DLB, dementia with Lewy bodies; FTD, frontotemporal dementia; MCI, mild cognitive impairment; PC, principal component; PCA, principal component analysis.

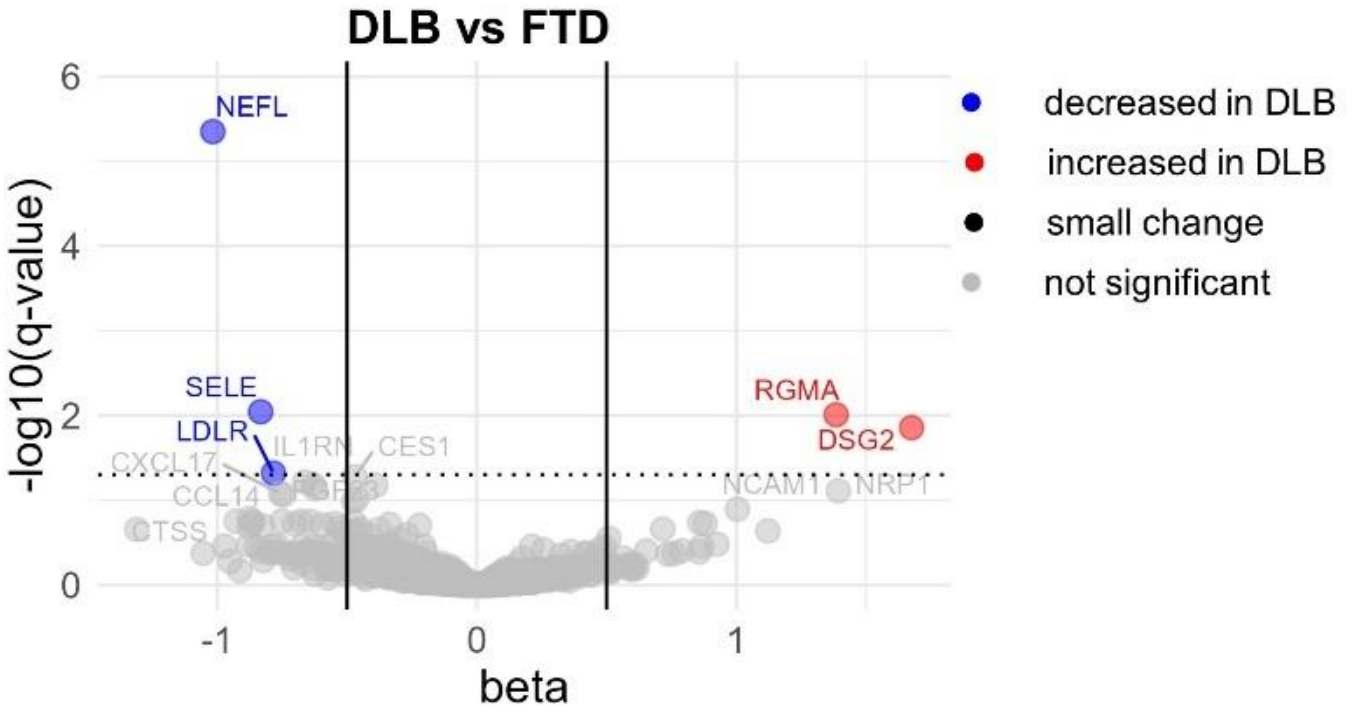

Supplementary Figure 2. DLB vs FTD. Volcano plots displaying -$\log_{10}$ of q-values (p-values adjusted for age, sex and multiple comparisons) on the y-axis and coefficients (beta) resulting from applying logistic regression on z-scored NPX data for the DLB (n=167) vs FTD (n=163). The horizontal line refers to q-value = 0.05, the two vertical represent arbitrary thresholds (± 0.5) for beta. Proteins are represented using the names of genes encoding for them, other abbreviations include DLB: dementia with Lewy bodies, FTD: frontotemporal dementia.

Prevalent pathology	N	age (y)	sex (%M)	NIA A	NIA B	NIA C	Braak α-syn	N Braak α-syn >3)
**AD**	103	86.8±6.8	17%	3.0 ± 0.1	3.0±0.1	2.8±0.5	1.7±2.2	24
**Lewy**	12	87.7±6.7	25%	2.8 ± 0.6	2.3±0.6	1.6±1.1	5.8±0.4	12
**Vascular**	14	86.5±4.2	29%	2.4 ± 0.9	1.6±0.6	1.4±1.1	0.2±0.8	0

Supplementary Table 1. Demographic and neuropathological features of the VARS cohort. *NIA A*, *NIA B* and *NIA C* refer to diffuse Aβ plaque burden (based on Thal phases; 0=0, 1=1/2, 2=3, 3=4), neurofibrillary tangle burden (based on Braak stages; 0=None, 1=I/II, 2=III/IV, 3=V/VI), and neuritic plaque location and density (based on the Consortium to Establish a Registry for Alzheimer's Disease [CERAD] score; 0=None, 1=Sparse, 2=Moderate, 3=Frequent), respectively. Each of the 3 components is scored 0–3. *Braak α-syn* refers to the Braak stages for α-synuclein, scored from 1-6, being 1 the onset of the disease with Lewy bodies in the lower brainstem and 6 the most severe stage of the disease with the presence of Lewy bodies in the neocortex. Numerical variables are reported as mean ± standard deviation, categorical variables as percentage. The reported age refers to the age at the time of blood sampling.

Group	N BL	Age at BL (y)	Sex (%M)	DD (y)	MoCA	H&Y	UPDRS-III	LEDD
**HC**	97	60.8±10.6	70%		28.2±1.1	0±0.1	1.1±2	0
**PD**	67	61.4±11.2	70%	0.4±0.5	27±2.2	1.5±0.5	20.7±9	1.8±13.2*
**proPD**	57	68.6±6.3	70%		26.3±3.2	0.1±0.4	4.2±4.5	0

Supplementary Table 2. Demographic and clinical characteristics at baseline (BL) of the participants of the PPMI 9000 study. Numerical variables are reported as mean ± standard deviation, categorical variables as percentage. Abbreviations BL: baseline, DD: disease duration, HC: healthy controls, H&Y: Hoen and Yahar scale, LEDD: levodopa equivalent dose; MoCA: Montreal cognitive assessment, M: males, UPDRS-III: unified Parkinson's disease rating scale part III. *A single proPD subject had 100 baseline LEDD.

protein	↑/↓ in AD	preAD vs CTRL	MCI-AD vs CTRL	AD-dem vs CTRL	AD-dem vs FTD	AD-dem vs DLB	avg
GFAP	↑	0.68 (0.64-0.73)	0.78 (0.73-0.83)	0.83 (0.79-0.87)	0.76 (0.71-0.81)	0.7 (0.65-0.76)	0.74
CALCOCO1	↓	0.62 (0.57-0.67)	0.7 (0.64-0.75)	0.8 (0.76-0.84)	0.71 (0.65-0.76)	0.74 (0.69-0.79)	0.72
MAP3K5	↓	0.6 (0.55-0.65)	0.71 (0.66-0.76)	0.79 (0.75-0.83)	0.68 (0.62-0.74)	0.74 (0.68-0.79)	0.71
BIN2	↓	0.61 (0.56-0.66)	0.7 (0.65-0.75)	0.79 (0.75-0.83)	0.68 (0.63-0.74)	0.72 (0.67-0.77)	0.7
SUSD1	↓	0.6 (0.54-0.65)	0.67 (0.62-0.72)	0.76 (0.71-0.8)	0.69 (0.63-0.74)	0.74 (0.68-0.79)	0.7
TRAF2	↓	0.6 (0.55-0.65)	0.68 (0.63-0.73)	0.78 (0.73-0.82)	0.68 (0.62-0.74)	0.72 (0.67-0.77)	0.7
TJAP1	↓	0.6 (0.55-0.65)	0.68 (0.63-0.73)	0.78 (0.73-0.82)	0.69 (0.63-0.74)	0.71 (0.66-0.77)	0.69
TBC1D23	↓	0.59 (0.54-0.65)	0.68 (0.63-0.74)	0.78 (0.74-0.83)	0.68 (0.62-0.73)	0.71 (0.66-0.76)	0.69
ZBTB16	↓	0.6 (0.55-0.66)	0.64 (0.58-0.69)	0.73 (0.69-0.78)	0.7 (0.64-0.75)	0.71 (0.66-0.77)	0.69
NFATC1	↓	0.59 (0.53-0.64)	0.63 (0.58-0.68)	0.74 (0.7-0.79)	0.71 (0.66-0.77)	0.7 (0.64-0.75)	0.69
MAVS	↓	0.59 (0.54-0.64)	0.69 (0.64-0.74)	0.78 (0.74-0.83)	0.68 (0.62-0.73)	0.7 (0.64-0.75)	0.69
EIF4G1	↓	0.61 (0.56-0.66)	0.69 (0.63-0.74)	0.77 (0.72-0.81)	0.67 (0.61-0.73)	0.7 (0.64-0.75)	0.68
SKAP2	↓	0.6 (0.55-0.65)	0.69 (0.63-0.74)	0.77 (0.72-0.81)	0.67 (0.61-0.73)	0.7 (0.65-0.76)	0.68
CRACR2A	↓	0.6 (0.55-0.66)	0.72 (0.67-0.77)	0.76 (0.72-0.81)	0.64 (0.59-0.7)	0.71 (0.65-0.76)	0.68
PDLIM7	↓	0.6 (0.55-0.66)	0.68 (0.63-0.73)	0.77 (0.73-0.82)	0.66 (0.61-0.72)	0.7 (0.65-0.76)	0.68
IRAG2	↓	0.6 (0.54-0.65)	0.72 (0.67-0.77)	0.78 (0.73-0.82)	0.63 (0.57-0.69)	0.71 (0.66-0.77)	0.68
TBC1D5	↓	0.61 (0.56-0.66)	0.73 (0.68-0.78)	0.76 (0.72-0.81)	0.64 (0.58-0.7)	0.71 (0.65-0.76)	0.68
STX8	↓	0.59 (0.54-0.65)	0.71 (0.66-0.76)	0.76 (0.72-0.81)	0.65 (0.59-0.71)	0.7 (0.65-0.76)	0.68
TARBP2	↓	0.59 (0.54-0.64)	0.65 (0.6-0.7)	0.75 (0.71-0.8)	0.68 (0.62-0.73)	0.69 (0.64-0.75)	0.68
CASP2	↓	0.6 (0.55-0.65)	0.67 (0.61-0.72)	0.75 (0.7-0.8)	0.67 (0.61-0.73)	0.69 (0.63-0.74)	0.68
SRC	↓	0.6 (0.55-0.65)	0.66 (0.61-0.71)	0.76 (0.71-0.81)	0.67 (0.61-0.72)	0.69 (0.64-0.75)	0.68
SCAMP3	↓	0.59 (0.54-0.64)	0.68 (0.63-0.73)	0.76 (0.71-0.81)	0.66 (0.61-0.72)	0.69 (0.63-0.74)	0.68
CLIP2	↓	0.59 (0.54-0.65)	0.65 (0.6-0.7)	0.75 (0.7-0.8)	0.68 (0.62-0.73)	0.69 (0.63-0.74)	0.68
HEXIM1	↓	0.59 (0.54-0.64)	0.65 (0.6-0.7)	0.74 (0.69-0.79)	0.68 (0.62-0.73)	0.69 (0.64-0.75)	0.68
ARHGEF12	↓	0.59 (0.54-0.64)	0.66 (0.6-0.71)	0.75 (0.7-0.79)	0.68 (0.63-0.74)	0.68 (0.62-0.74)	0.68
F11R	↓	0.57 (0.51-0.62)	0.66 (0.61-0.72)	0.74 (0.7-0.79)	0.67 (0.61-0.72)	0.7 (0.65-0.76)	0.67
AXIN1	↓	0.59 (0.54-0.64)	0.63 (0.58-0.69)	0.73 (0.68-0.78)	0.69 (0.64-0.75)	0.67 (0.62-0.73)	0.67
STX4	↓	0.61 (0.56-0.66)	0.71 (0.65-0.76)	0.76 (0.71-0.8)	0.64 (0.59-0.7)	0.68 (0.63-0.74)	0.67
MAP2K6	↓	0.59 (0.54-0.64)	0.65 (0.6-0.7)	0.74 (0.7-0.79)	0.67 (0.61-0.73)	0.69 (0.63-0.74)	0.67
IRAK1	↓	0.59 (0.54-0.64)	0.66 (0.61-0.72)	0.74 (0.7-0.79)	0.67 (0.61-0.73)	0.67 (0.62-0.73)	0.67
CORO1A	↓	0.61 (0.56-0.66)	0.71 (0.66-0.76)	0.77 (0.72-0.81)	0.63 (0.57-0.69)	0.67 (0.62-0.73)	0.67
YES1	↓	0.59 (0.54-0.64)	0.62 (0.57-0.68)	0.74 (0.69-0.78)	0.68 (0.62-0.73)	0.67 (0.62-0.73)	0.67
VPS37A	↓	0.59 (0.54-0.64)	0.66 (0.61-0.71)	0.75 (0.7-0.8)	0.66 (0.61-0.72)	0.67 (0.61-0.72)	0.67
IKBKG	↓	0.59 (0.53-0.64)	0.64 (0.59-0.7)	0.72 (0.68-0.77)	0.68 (0.63-0.74)	0.66 (0.6-0.72)	0.66
BANK1	↓	0.59 (0.53-0.64)	0.63 (0.57-0.68)	0.74 (0.69-0.78)	0.69 (0.64-0.75)	0.65 (0.6-0.71)	0.66
MPIG6B	↓	0.59 (0.54-0.64)	0.63 (0.58-0.68)	0.71 (0.66-0.76)	0.67 (0.61-0.72)	0.68 (0.63-0.74)	0.66
INPPL1	↓	0.59 (0.54-0.64)	0.61 (0.56-0.67)	0.71 (0.66-0.76)	0.69 (0.63-0.74)	0.67 (0.61-0.72)	0.66
SNAP29	↓	0.6 (0.55-0.65)	0.64 (0.58-0.69)	0.72 (0.68-0.77)	0.67 (0.61-0.72)	0.67 (0.61-0.72)	0.66
MGLL	↓	0.6 (0.55-0.65)	0.63 (0.58-0.69)	0.73 (0.68-0.77)	0.67 (0.61-0.72)	0.67 (0.61-0.72)	0.66
CDKN1A	↓	0.6 (0.54-0.65)	0.6 (0.55-0.66)	0.72 (0.67-0.77)	0.69 (0.63-0.75)	0.65 (0.6-0.71)	0.66
PLXNA4	↓	0.58 (0.53-0.64)	0.64 (0.59-0.69)	0.7 (0.65-0.75)	0.67 (0.61-0.73)	0.67 (0.61-0.73)	0.66
DBNL	↓	0.6 (0.55-0.65)	0.66 (0.6-0.71)	0.74 (0.69-0.78)	0.65 (0.59-0.71)	0.66 (0.61-0.72)	0.66
MVK	↓	0.59 (0.54-0.65)	0.63 (0.58-0.68)	0.71 (0.66-0.76)	0.68 (0.63-0.74)	0.65 (0.6-0.71)	0.66
AIFM1	↓	0.59 (0.54-0.64)	0.64 (0.58-0.69)	0.72 (0.67-0.76)	0.66 (0.61-0.72)	0.66 (0.61-0.72)	0.66
CASP3	↓	0.6 (0.54-0.65)	0.63 (0.57-0.68)	0.71 (0.67-0.76)	0.67 (0.61-0.73)	0.66 (0.6-0.72)	0.66
CIAPIN1	↓	0.6 (0.55-0.65)	0.64 (0.58-0.69)	0.73 (0.68-0.78)	0.65 (0.6-0.71)	0.66 (0.6-0.72)	0.66
CLEC1B	↓	0.6 (0.55-0.65)	0.62 (0.57-0.68)	0.71 (0.67-0.76)	0.66 (0.6-0.72)	0.67 (0.61-0.72)	0.66

PMVK	↓	0.61 (0.56-0.66)	0.6 (0.55-0.66)	0.7 (0.65-0.75)	0.67 (0.61-0.73)	0.67 (0.61-0.73)	0.66
ABL1	↓	0.58 (0.53-0.64)	0.66 (0.61-0.72)	0.74 (0.7-0.79)	0.64 (0.58-0.7)	0.67 (0.61-0.72)	0.66
CD40	↓	0.56 (0.51-0.61)	0.63 (0.57-0.68)	0.72 (0.68-0.77)	0.65 (0.59-0.71)	0.68 (0.63-0.74)	0.66

Supplementary Table 3. Area under the ROC curve (AUC) values for top 50 AD-specific plasma proteins ranked by average AUC in the bPRIDE discovery cohort. Average AUC value (avg) was calculated by averaging AUC values of the preAD vs CTRL, MCI-AD vs CTRL and AD-dem vs CTRL comparisons and then average this value with those produced by the AD-dem vs DLB and AD-dem vs FTD comparisons. AUC values for each comparison are also shown, with 95% confidence intervals in brackets calculated by the Hanley and McNeil's method. AD specificity was defined based on the consistent sign of beta values (logistic regression coefficient) and q-value < 0.05 in all the above-considered comparisons. Arrows are used to describe whether the protein considered was found to be increased (↑) or decreased (↓) in AD.

protein	↑/↓ in DLB	DLB vs CTRL	DLB vs AD-dem	avg
ITGAV	↓	0.78 (0.73-0.82)	0.68 (0.63-0.74)	0.73
ITGAM	↓	0.75 (0.71-0.8)	0.68 (0.62-0.73)	0.72
ITGB2	↓	0.72 (0.67-0.77)	0.66 (0.6-0.71)	0.69
PI3	↑	0.71 (0.66-0.76)	0.65 (0.59-0.7)	0.68
B4GAT1	↓	0.75 (0.7-0.8)	0.61 (0.56-0.67)	0.68
DPP4	↓	0.73 (0.68-0.77)	0.62 (0.56-0.68)	0.67
CRTAC1	↓	0.65 (0.6-0.71)	0.65 (0.59-0.71)	0.65
MYOC	↓	0.71 (0.66-0.76)	0.57 (0.51-0.63)	0.64
OMG	↓	0.68 (0.63-0.73)	0.6 (0.54-0.66)	0.64
GDNF	↑	0.63 (0.58-0.68)	0.63 (0.57-0.69)	0.63
CLEC4A	↓	0.62 (0.57-0.67)	0.63 (0.57-0.69)	0.63
CD276	↑	0.64 (0.59-0.69)	0.59 (0.53-0.65)	0.61
COMP	↓	0.63 (0.58-0.68)	0.59 (0.53-0.65)	0.61
TFF3	↑	0.6 (0.55-0.65)	0.56 (0.5-0.62)	0.58
IGFBP1	↓	0.57 (0.52-0.63)	0.59 (0.53-0.65)	0.58
TACSTD2	↓	0.58 (0.53-0.63)	0.58 (0.52-0.64)	0.58
CA6	↓	0.58 (0.53-0.63)	0.58 (0.51-0.64)	0.58
OGN	↑	0.58 (0.52-0.63)	0.54 (0.48-0.6)	0.56
DSG2	↑	0.56 (0.5-0.61)	0.55 (0.49-0.62)	0.56

Supplementary Table 4. Area under the ROC curve (AUC) values for 19 DLB-specific plasma proteins ranked by average AUC. Average AUC value (avg) was calculated by averaging AUC values of the DLB vs CTRL and DLB vs AD-dem comparisons. AUC values for each comparison are also shown, with 95% confidence intervals in brackets calculated by the Hanley and McNeil's method. DLB specificity was defined based on the consistent sign of beta values (logistic regression coefficient) and q-value < 0.05 in the two comparisons. Arrows are used to describe whether the protein considered was found to be increased (↑) or decreased (↓) in DLB.

protein	↑/↓ in FTD	FTD vs CTRL	FTD vs AD-dem	avg
NEFL	↑	0.81 (0.76-0.85)	0.64 (0.58-0.69)	0.72
ITGAV	↓	0.73 (0.68-0.78)	0.64 (0.58-0.69)	0.68
NCAN	↓	0.68 (0.63-0.73)	0.67 (0.61-0.73)	0.68
MYOC	↓	0.73 (0.68-0.78)	0.6 (0.54-0.66)	0.67
COMP	↓	0.67 (0.62-0.73)	0.63 (0.57-0.69)	0.65
NTRK3	↓	0.65 (0.59-0.7)	0.65 (0.59-0.7)	0.65
ITGAM	↓	0.68 (0.63-0.73)	0.61 (0.55-0.67)	0.64
CRTAC1	↓	0.64 (0.59-0.69)	0.64 (0.58-0.69)	0.64
CD34	↓	0.65 (0.6-0.7)	0.6 (0.54-0.66)	0.63
CNTN4	↓	0.65 (0.6-0.7)	0.59 (0.53-0.66)	0.62
PON3	↓	0.65 (0.6-0.7)	0.59 (0.53-0.65)	0.62
NOTCH3	↓	0.61 (0.56-0.67)	0.62 (0.57-0.68)	0.62
DEFB4A	↑	0.63 (0.58-0.68)	0.59 (0.53-0.66)	0.61
CST6	↑	0.62 (0.57-0.67)	0.6 (0.54-0.66)	0.61
HPGDS	↓	0.66 (0.61-0.72)	0.56 (0.5-0.62)	0.61
IGFBP1	↓	0.6 (0.55-0.66)	0.62 (0.56-0.68)	0.61
F3	↓	0.62 (0.56-0.67)	0.6 (0.54-0.66)	0.61
RGMA	↓	0.64 (0.59-0.69)	0.56 (0.5-0.62)	0.6
SCGB3A2	↓	0.6 (0.55-0.66)	0.58 (0.52-0.64)	0.59
COL6A3	↑	0.56 (0.5-0.61)	0.58 (0.52-0.64)	0.57
CA6	↓	0.56 (0.51-0.62)	0.56 (0.5-0.62)	0.56
FABP4	↑	0.52 (0.46-0.57)	0.52 (0.46-0.58)	0.52
MMP3	↑	0.51 (0.46-0.56)	0.53 (0.47-0.59)	0.52

Supplementary Table 5. Area under the ROC curve (AUC) values for 23 FTD-specific plasma proteins ranked by average AUC. Average AUC value (avg) was calculated by averaging AUC values of the FTD vs CTRL and FTD vs AD-dem comparisons. AUC values for each comparison are also shown, with 95% confidence intervals in brackets calculated by the Hanley and McNeil's method FTD specificity was defined based on the consistent sign of beta values (logistic regression coefficient) and q-value < 0.05 in the two comparisons. Arrows are used to describe whether the protein considered was found to be increased (↑) or decreased (↓) in FTD.

panel protein	DLB vs AD-dem	DLB vs CTRL	FTD vs AD-dem	FTD vs CTRL	AD staging
ITGAM	-0.12	-0.8		-0.197	
ITGAV	-0.7	-0.9		-0.325	-0.217
CD38	0.44				
CALCOCO1	0.24				
FLT3LG		-0.4		-0.056	-0.32
MAP3K5				-0.01	-0.004
IRAG2				-0.13	
B4GAT1				-0.81	
TRAF2					-0.019
TNFSF10				-0.325	-0.23
OSM				0.138	0.075
NCAN				-0.315	
NEFL			1	1.319	0.06
RET					-0.144
PI3		0.03			
GFAP	-0.36	0.07	-1		0.245
PECAM1					-0.07
CDKN1A					
EGFR					
ERBB3		-1			
NTRK3					

Supplementary Table 6. Coefficients of multivariable models. The LASSO coefficients are reported for the DLB vs AD-dem, DLB vs CTRL, FTD vs AD-dem, FTD vs CTRL comparisons and AD staging. Proteins are represented using the names of genes encoding for them, other abbreviations include AD: Alzheimer's disease, AD-dem: Alzheimer's disease at dementia stage, CTRL: controls, DLB: dementia with Lewy bodies, FTD: frontotemporal dementia, LASSO: least absolute shrinkage selection operator.

Cohort	Aβ assay and cutoff	Tau assay and cutoff
ADC	Innotest ELISA CSF $A\beta_{42}$<813 pg/mL<br>Elecsys CSF $A\beta_{42}$<1000[1] pg/mL	Innotest ELISA CSF pTau>52 pg/mL<br>Elecsys CSF pTau>24 pg/mL[2]
AIBL	Aβ PET >25 centiloids[3]	Tau PET meta temporal SUVR>1.19[4]
BioFINDER	Euroimmun ELISA CSF $A\beta_{42}/A\beta_{40}$<0.088	Euroimmun pTau181>65.04 pg/mL or tTau>462.91 pg/mL
SPIN	Lumipulse CSF $A\beta_{42}/A\beta_{40}$<0.062	Lumipulse CSF pTau181>63 pg/mL or tTau>456 pg/mL
UNIPG	Lumipulse CSF $A\beta_{42}/A\beta_{40}$<0.072[5]	Lumipulse CSF pTau181>50 pg/mL[5]
UU	Lumipulse CSF $A\beta_{42}$<450 pg/mL	Lumipulse pTau181>65 pg/mL or tTau>380 pg/mL
CBAS	Lumipulse CSF $A\beta_{42}$<526 pg/mL<br>or A Aβ PET (visual assessment)[6]	Lumipulse p-tau181 > 50.2 pg/mL[6]

Supplementary Table 7. Biomarker assays and cutoff values for determining the A and T biological anchors in the multicentric bPRIDE cohort. Abbreviations. Aβ: amyloid-beta, ADC: Amsterdam Dementia Cohort; AIBL: Australian Imaging Biomarker & Lifestyle Flagship Study of Ageing cohort, BioFINDER: The Swedish BioFINDER study cohort, CSF; cerebrospinal fluid, ELISA: enzyme-linked immunosorbent assay, SPIN: Sant Pau Initiative on Neurodegeneration, Ulm: University of Ulm cohort, UNIPG: University of Perugia cohort.

Assay	CV intra-plate (%)	CV inter-run (%)	CV Intra-run inter-plate (%)	CV overall (%)
B4GAT1	5.9	6.7	10.2	11.9
CALCOCO1	5.9	11.8	9.6	15.0
CD38	5.4	12.7	6.4	14.3
CDKN1A	11.3	12.6	7.6	17.0
EGFR	7.0	13.8	6.6	15.5
ERBB3	5.9	13.0	5.7	14.8
FLT3LG	6.0	13.3	6.7	15.3
GFAP	8.4	25.2	10.3	25.8
IRAG2	6.9	8.7	7.3	12.4
ITGAM	5.4	10.6	4.2	12.2
ITGAV	4.4	8.3	7.2	11.1
MAP3K5	18.3	19.3	16.2	26.8
NCAN	5.5	11.6	4.6	13.4
NEFL	16.8	19.6	15.3	27.2
NTRK3	7.0	14.9	6.7	16.8
OSM	6.4	12.7	6.7	14.8
PECAM1	6.3	14.4	7.2	16.0
PI3	6.5	17.1	6.6	18.7
RET	5.9	13.3	5.8	14.9
TNFSF10	4.9	10.8	5.8	12.6
TRAF2	5.5	11.2	6.2	13.2

Supplementary Table 8. Mean assay-specific coefficients of variation (CVs) for the custom panel, summarizing precision across runs and plates. For each analyte, we calculated four CVs, intra-replicate, inter-plate (within run), inter-run, and overall, based on absolute quantification values obtained from 3 technical replicates × 4 internal control plasma pools included on every plate in each run. Reported CVs are means across the four control pools. All mean CVs were <30% with the great majority being<20%, indicating robust technical performance of the custom PEA panel applied for validation.

Mean CV intra-plate control (%)					
**Panel**	**plate A**	**plate B**	**plate C**	**plate D**	**Average**
Cardiometabolic	14.34	18.31	5.75	14.66	13.26
Inflammation	17.00	16.32	5.58	12.77	12.92
Neurology	20.50	14.93	8.39	11.06	13.72
Oncology	16.94	15.06	6.50	12.06	12.64
**Mean CV inter-plate control (%)**					
**Panel**	**plate A**	**plate B**	**plate C**	**plate D**	**Average**
Cardiometabolic	20.37	23.77	10.06	15.88	17.52
Inflammation	21.74	20.60	10.75	16.45	17.39
Neurology	25.64	19.28	17.34	17.57	19.96
Oncology	23.34	21.74	12.73	17.83	18.91

Supplementary Table 9. Protein-specific intra- and inter-plate coefficients of variation (CVs) across PEA panels. Four types of CVs were calculated for each protein using both internal control samples (2 per plate) and plate controls (3 per plate): intra-plate CVs and inter-plate CVs for both control types. The table reports average CVs by panel across four assay plates (A–D). All mean CVs were below 20%, supporting the technical reliability of the measurements performed in the discovery cohort.

Protein	Slope	Intercept
B4GAT1	1.07	-16.27
CALCOCO1	0.89	-1.14
CD38	0.98	-6.21
CDKN1A	0.98	-5.35
EGFR	0.69	-10.29
ERBB3	0.73	-9.14
FLT3LG	0.83	-5.03
GFAP	0.62	-5.23
IRAG2	1.23	-3.96
ITGAM	0.74	-9.23
ITGAV	0.47	-3.67
MAP3K5	0.97	-9.11
NCAN	1.09	-10.31
NEFL	0.55	-4.05
NTRK3	0.89	-10.59
OSM	0.75	-0.49
PECAM1	1.07	-14.73
PI3	0.81	-10.49
RET	0.90	-8.47
TNFSF10	0.97	-7.69
TRAF2	1.07	-11.58

Supplementary Table 10. Discovery/Validation data calibration coefficients. Slope and intercept values needed to convert log_2 of quantified values obtained from the validation database into ranges of discovery database NPX values.
