# Supplementary material for: Plasma proteome profiling identified biomarkers for the differential diagnosis and molecular staging of neurodegenerative dementias
Source: Nat Aging. 2026 Jul 20;6(8):1717–32. doi: 10.1038/s43587-026-01162-7 (PMC13472907; doi:10.1038/s43587-026-01162-7)
Supplement: Supplementary file 2 [file 43587_2026_1162_MOESM2_ESM.pdf]

nature portfolio

Corresponding author(s): Giovanni Bellomo, Charlotte Teunissen and Marta del Campo

Last updated by author(s): Apr 15, 2026

Reporting Summary

Nature Portfolio wishes to improve the reproducibility of the work that we publish. This form provides structure for consistency and transparency in reporting. For further information on Nature Portfolio policies, see our Editorial Policies and the Editorial Policy Checklist.

Statistics

For all statistical analyses, confirm that the following items are present in the figure legend, table legend, main text, or Methods section.

n/a	Confirmed	
☐	☒	The exact sample size (n) for each experimental group/condition, given as a discrete number and unit of measurement
☐	☒	A statement on whether measurements were taken from distinct samples or whether the same sample was measured repeatedly
☐	☒	The statistical test(s) used AND whether they are one- or two-sided<br>*Only common tests should be described solely by name; describe more complex techniques in the Methods section.*
☐	☒	A description of all covariates tested
☐	☒	A description of any assumptions or corrections, such as tests of normality and adjustment for multiple comparisons
☐	☒	A full description of the statistical parameters including central tendency (e.g. means) or other basic estimates (e.g. regression coefficient) AND variation (e.g. standard deviation) or associated estimates of uncertainty (e.g. confidence intervals)
☐	☒	For null hypothesis testing, the test statistic (e.g. F, t, r) with confidence intervals, effect sizes, degrees of freedom and P value noted<br>*Give P values as exact values whenever suitable.*
☒	☐	For Bayesian analysis, information on the choice of priors and Markov chain Monte Carlo settings
☐	☒	For hierarchical and complex designs, identification of the appropriate level for tests and full reporting of outcomes
☐	☒	Estimates of effect sizes (e.g. Cohen's d, Pearson's r), indicating how they were calculated

Our web collection on statistics for biologists contains articles on many of the points above.

Software and code

Policy information about availability of computer code

Data collection	Plasma protein data were generated using Olink Proteomics proximity extension assay (PEA) platforms (Explore 384 panels and custom assays), following manufacturer protocols. Clinical and demographic data were collected using standardized procedures at each participating center.
Data analysis	No relevant custom code was developed for this study. Statistical analyses were performed using Python 3.0, R version 4.2.3, and OriginLab 9. Key analytical packages included glmnet (LASSO regression), pROC (ROC analysis and bootstrap confidence intervals), and KEGGREST (pathway enrichment analysis), as described in the Methods. Proteomic measurements were generated using validated Olink Explore 384 PEA and custom Olink PEA assay workflows. BioRender was used for graphical elements in Figure 1.

For manuscripts utilizing custom algorithms or software that are central to the research but not yet described in published literature, software must be made available to editors and reviewers. We strongly encourage code deposition in a community repository (e.g. GitHub). See the Nature Portfolio guidelines for submitting code & software for further information.

April 2023

Data

Policy information about availability of data

All manuscripts must include a data availability statement. This statement should provide the following information, where applicable:

- Accession codes, unique identifiers, or web links for publicly available datasets
- A description of any restrictions on data availability
- For clinical datasets or third party data, please ensure that the statement adheres to our policy

The source data underlying all figures in the main text, Extended Data, and Supplementary Material are provided with this paper. The proteomics datasets supporting the findings of this study have been deposited in Synapse and are publicly available at https://www.synapse.org/Synapse:syn75233632.

Research involving human participants, their data, or biological material

Policy information about studies with human participants or human data. See also policy information about sex, gender (identity/presentation), and sexual orientation and race, ethnicity and racism.

Reporting on sex and gender	Biological sex was recorded for all participants and included in the statistical analyses as a covariate where appropriate. No analyses based on gender identity were performed.
Reporting on race, ethnicity, or other socially relevant groupings	Information on race and ethnicity was not systematically collected (and used) across all cohorts. However, participants were predominantly of White/European ancestry, reflecting the geographical origin of the cohorts. Race and ethnicity were not included in the statistical analyses.
Population characteristics	Age and sex were consistently used as covariates, a specific analysis on co-morbindities was also included
Recruitment	Participants were recruited from multiple international cohorts, primarily memory clinic–based studies, including the Amsterdam Dementia Cohort (ADC), Sant Pau Initiative on Neurodegeneration (SPIN), University of Perugia (UNIPG), Ulm University (UU), BioFINDER, and the Australian Imaging, Biomarkers and Lifestyle study (AIBL) in the discovery phase, and ADC, SPIN, UNIPG, UU, AIBL, and the Czech Brain Aging Study (CBAS) in the validation phase. Inclusion criteria and diagnostic classification were defined locally at each site according to established international consensus criteria.
Ethics oversight	All procedures involving human participants were conducted in accordance with the Declaration of Helsinki and the ethical standards of the relevant institutional and national research committees. All participants provided written informed consent for the use of clinical data and biomaterials for research purposes. The study was approved by the relevant local ethics committees at participating centers, including the Comitato Etico Aziende Sanitarie Regione Umbria (19369/AV and 20942/21/OV), the Amsterdam UMC VUmc Medical Ethics Committee (2019.102), the Sant Pau Ethics Committee (SPIN cohort), the Ethics Committees of Motol University Hospital and St. Anne's University Hospital (CBAS cohort), and the relevant ethics/biobank governance bodies overseeing the Institute Born-Bunge/BIODEM cohort. Local health authorities approved the VARS patient follow-up program at the Queen Sofía Alzheimer's Center (Ref: MCB/RMSFC, Expte: 12672).

Note that full information on the approval of the study protocol must also be provided in the manuscript.

Field-specific reporting

Please select the one below that is the best fit for your research. If you are not sure, read the appropriate sections before making your selection.

☒ Life sciences ☐ Behavioural & social sciences ☐ Ecological, evolutionary & environmental sciences

For a reference copy of the document with all sections, see nature.com/documents/nr-reporting-summary-flat.pdf

Life sciences study design

All studies must disclose on these points even when the disclosure is negative.

Sample size	Sample sizes were determined based on the availability of well-characterized participants across cohorts, with a minimum target of >25 individuals per group in the discovery phase. In the validation phase, sample sizes were further expanded to enable the assessment of effects in prodromal groups.
Data exclusions	Predefined quality control criteria were applied. Samples with more than 35% quality control warnings and proteins with more than 15% values below the limit of detection or associated with quality control warnings were excluded, as described in the Methods section.
Replication	Findings from the discovery cohort were replicated in an independent multicenter validation cohort analyzed using a quantitative custom multiplex assay. Additional validation was performed in independent external datasets.
Randomization	No randomization was used to allocate participants to diagnostic groups, as group assignment was based on predefined clinical and biomarker criteria. Plasma samples were randomized across assay plates prior to proteomic measurements to minimize potential batch effects, and participants were randomly assigned to training and test sets using stratified sampling within each diagnostic group to preserve class proportions during model development.

Blinding	Proteomic measurements were performed blinded to clinical diagnosis in both the discovery and validation cohorts.

Reporting for specific materials, systems and methods

We require information from authors about some types of materials, experimental systems and methods used in many studies. Here, indicate whether each material, system or method listed is relevant to your study. If you are not sure if a list item applies to your research, read the appropriate section before selecting a response.

Materials & experimental systems

n/a	Involved in the study
☒	☐ Antibodies
☒	☐ Eukaryotic cell lines
☒	☐ Palaeontology and archaeology
☒	☐ Animals and other organisms
☐	☒ Clinical data
☒	☐ Dual use research of concern
☒	☐ Plants

Methods

n/a	Involved in the study
☒	☐ ChIP-seq
☒	☐ Flow cytometry
☒	☐ MRI-based neuroimaging

Clinical data

Policy information about clinical studies

All manuscripts should comply with the ICMJE guidelines for publication of clinical research and a completed CONSORT checklist must be included with all submissions.

Clinical trial registration	This study is not a clinical trial and was therefore not registered.
Study protocol	The study protocol was defined a priori within the framework of the multicenter bPRIDE (blood Proteins for early Discrimination of dEmentias) project, funded under the JPND (EU Joint Programme – Neurodegenerative Disease Research) initiative, and is described in the Methods section.
Data collection	Data collection was performed using standardized procedures across participating cohorts, including clinical assessments and plasma protein measurements using Olink proximity extension assay (PEA) platforms.
Outcomes	The primary outcomes of the bPRIDE project were the identification of plasma protein biomarkers for the differential diagnosis of Alzheimer's disease, dementia with Lewy bodies, and frontotemporal dementia, and the development and validation of a multiplex biomarker panel.

Plants

Seed stocks	This study did not involve plants.
Novel plant genotypes	This study did not involve plants.
Authentication	This study did not involve plants.
