# Supplementary material for: Plasma proteome profiling identified biomarkers for the differential diagnosis and molecular staging of neurodegenerative dementias
Source: Nat Aging. 2026 Jul 20;6(8):1717–32. doi: 10.1038/s43587-026-01162-7 (PMC13472907; doi:10.1038/s43587-026-01162-7)
Supplement: Supplementary file 3 [file 43587_2026_1162_MOESM3_ESM.pdf]

nature portfolio

Peer Review File

Plasma proteome profiling identified biomarkers for the differential diagnosis and molecular staging of neurodegenerative dementias

Corresponding Author: Professor Giovanni Bellomo

This manuscript has been previously reviewed at another journal. This document only contains information relating to versions considered at Nature Aging.

Version 0:

Reviewer comments:

Reviewer #1

(Remarks to the Author)
The authors have satisfactorily addressed each of my critiques.

Reviewer #2

(Remarks to the Author)
I am happy with this revised version of the manuscript.

Reviewer #3

(Remarks to the Author)
The authors have satisfactorily addressed all my comments, and I am pleased with the revisions made.
I have no further concerns.

Open Access This Peer Review File is licensed under a Creative Commons Attribution 4.0 International License, which permits use, sharing, adaptation, distribution and reproduction in any medium or format, as long as you give appropriate credit to the original author(s) and the source, provide a link to the Creative Commons license, and indicate if changes were made.
In cases where reviewers are anonymous, credit should be given to 'Anonymous Referee' and the source.
The images or other third party material in this Peer Review File are included in the article's Creative Commons license, unless indicated otherwise in a credit line to the material. If material is not included in the article's Creative Commons license and your intended use is not permitted by statutory regulation or exceeds the permitted use, you will need to obtain permission directly from the copyright holder.
To view a copy of this license, visit https://creativecommons.org/licenses/by/4.0/

Herewith we provide a point-by-point response (in black) to the Reviewers' comments (in blue). Significant changes within the text are highlighted in yellow to facilitate review process. Taken together, we have carefully addressed all points raised by the referees and we hope that reviewers and the editor find our paper now acceptable for publication.

Many thanks for considering our manuscript.

Best regards,

Marta Del Campo, Giovanni Bellomo and Charlotte Teunissen

Reviewer #1

This paper used Olink to examine the plasma proteome for biomarkers for neurodegenerative dementias in a large cohort of AD, DLB and FTD patients. The results confirmed GFAP and NfL as promising biomarkers for AD and neurodegeneration, respectively (the methods used did not include p-tau markers for AD pathology). A few new potential biomarkers for DLB (a disease in urgent need of better diagnostics) were identified (e.g., the integrin proteins ITGAV and ITGAM), but these data were not replicated. The work resulted in a 21-biomarker panel (this number of biomarkers was chosen based on the technical limitations of Olink). The diagnostic performance of the 21-biomarker panel was not tested on external cohorts, and not in relation to existing biomarkers for AD, FTD and DLB pathophysiology. The 21-biomarker panel had minimal overlap with a previously published 21-biomarker Olink panel for AD (Jiang et al., A&D, 2024; this study is cited but not discussed).

We agree with the reviewer that replication and validation of discovery findings are necessary. It should be noted that we have already observed consistent changes (e.g., in the newly identified DLB biomarkers ITGAV and ITGAM among others) across multiple independent datasets, including the PPMI and VARS cohorts, both of which involve individuals with synucleinopathies. In the autopsy-confirmed cohort, we further demonstrated a significant correlation between ITGAV levels and α-synuclein Braak staging, reinforcing its potential pathological relevance. These findings offer strong supportive evidence across different disease models. Importantly, in this new revised version we have added information and results around our newly developed and validated a custom multiplex assay, capable of quantifying the 21 biomarkers with absolute concentrations (pg/mL). We have performed clinical validation of this assay in an independent, multicenter cohort comprising 805 subjects (722 after precleaning) including patients spanning the full AD continuum, as well as prodromal and symptomatic DLB and FTD cases, which show similar biomarker changes and diagnostic performances as observed with the discovery data. The Reviewer may find all the newly added info and analyses in the multiple highlighted sections throughout the manuscript.

In particular, the basic clinical and demographic details of the newly added multicenter validation cohort are summarized in the revised Table 1, the diagnostic performance of the 21 quantified plasma biomarkers is summarized int newly added Figure 7, while biomarkers distributions across all diagnostic groups are shown in Figure S10. We also updated Figure 6 by including the performance of the previously trained multivariable models in the validation cohort. Herewith, we thus provide a single, externally validated, multiplex tool for plasma-based differential diagnosis of the most common neurodegenerative dementias, which we consider to be an important advancement for both clinical and research applications.

Reviewer #2

Remarks to the Author:

In the present study authors brought together 6 international cohorts to examine the extent to which plasma proteins were differentially expressed in and could discriminate between Alzheimer's disease, frontotemporal dementia (FTD), and dementia with Lew bodies (DLB) using proteins measured from the Olink proximity extension assay (PEA). The authors identify a number of differentially expressed proteins in plasma and make a multi-protein panel that can differentiate dementia from non-dementia, stage dementia types, and differentiate among dementia etiologies with good accuracy. Many of the proteins have been identified previously, but there are a number of novel candidate proteins as well. This paper doesn't elucidate the biological significance of these findings much, but rather focuses on diagnostic score development. The application of the same proteomic platform to multiple well-phenotyped cohorts with differing dementia etiologies is commendable. I have several critiques for the authors to consider.

The authors state "However, distinguishing between AD, DLB, and FTD remains difficult, given the overlapping symptoms and high degree of co-pathology/mixed pathologies across the different neurodegenerative diseases." However, this isn't necessarily the case, especially not for FTD. The clinical course (e.g., age of onset and symptom presentation) differ considerably from AD and DLB. There is not a whole lot of concern about – for example – mistaking AD for FTD. AD vs DLB there is more overlap, but the issue is less distinguishing one from the other. It's more about to what degree is there DLB pathology in the this AD patient and vice versa. Consider revising the discussion to better reflect the current clinical needs and shortcomings.

We appreciate the reviewer's comment and agree that in some cases, particularly in later disease stages, FTD can be differentiated from AD and DLB based on clinical presentation. However, substantial diagnostic uncertainty remains, especially early in the disease course or in patients with atypical features. For example, the behavioral variant of FTD can overlap clinically with atypical AD or primary psychiatric disorders, and the semantic or non-fluent variants of primary progressive aphasia may also pose significant diagnostic challenges.

Even in the context of AD, where clinical criteria are well established, biomarker-informed approaches considerably improve diagnostic accuracy. Palmqvist et al. (2024) demonstrated that accuracy based solely on clinical assessment is approximately 73% among dementia specialists and 61% among primary care physicians but increases to 91% when applying the Amyloid Probability Score 2 (APS2). These findings underscore the added value of biomarker-based tools in improving diagnostic precision across neurodegenerative diseases. Additionally, the presence of co-pathologies further complicates differential diagnosis based on clinical features alone. Despite clinical phenotypes being considered, fluid biomarkers are needed to streamline and improve not only diagnostic workflow but also patient selection and stratification in clinical trials.

To reflect this nuance, we have revised the sentence in the introduction and discussion as follows:

1. Introduction (Page 2, lines 24-29): "However, fluid biomarkers for AD staging or for reliably identifying FTD and DLB are still lacking: This poses a significant challenge in distinguishing between AD, DLB, and FTD, as these conditions often exhibit overlapping clinical symptoms and considerable heterogeneity, especially in early stages or atypical presentations. The presence of co-existing pathologies further complicates the diagnostic process by blurring disease-specific features and reducing diagnostic specificity.[5]"
2. Discussion (Page 12, lines 16-22): "Distinguishing between AD, DLB, and FTD remains a clinical challenge, particularly in early stages or atypical presentations. While FTD can often be distinguished from AD and DLB by age of onset and symptom profile, overlap still occurs,

and AD–DLB differentiation is further complicated by frequent co-pathologies[49–51]. Of note, there are no plasma biomarkers for either DLB or FTD. These limitations highlight the need for fluid biomarkers that can complement clinical assessment, improve diagnostic and support patient selection and stratification in research and therapeutic settings."

I suspect that what proteins come up as top candidates here depends heavily on which proteomic platforms were employed. Please clarify why the select proteomic platforms were selected, especially Oncology and Cardiometabolic, which have a less obvious connection to neurodegenerative disease. In the discussion the authors should comment on how the proteomic panel selection – as opposed to, for example, untargeted mass spec proteomics - may have affected their results.

The main aim of the study is to identify biomarkers that can be translated into optimal tools for diagnostic purposes. We decided to use the PEA-Olink as discovery platform based on our previous successful CSF proteomics and follow-up studies, which show the capabilities of this technology to identify markers for each type of dementia and to efficiently translate findings into custom analytical tools, including those developed in different platforms (e.g., ELLA or Simoa; Hok-a-Hin et al, molecular neurodegeneration, 2025; Hok-a-Hin et al, AR&T, in press; Del Campo et al, Nat Med 2022; Del Campo et al, Nat Comm 2023; Del Campo et al, Molec Neurodegeneration 2024; Hok-A-Hin et al, AD:DADM, 2023; Hok-a-Hin et al, J.Neuroinflamm, 2023). We would like to clarify that the labels like "Oncology", "Inflammation", "Neurology" and "Cardiometabolic" are just commercial designations. For example, GFAP, a protein of central relevance in the field of neurodegenerative disorders, was included in the Oncology panel by the PEA kit producer (based on the evidence that increased GFAP levels are associated to glioblastoma). Regarding the selection of proteins measured by PEA, we used <u>all panels</u> that were available at the time of measurement (i.e., Explore 384 Cardiometabolic, Explore 384 Inflammation, Explore 384 Neurology, Explore 384 Oncology) and therefore did not apply any selection criteria. This was indicated in the method section in the original version (now page 5, lines 6-7). Concerning the choice of methods and the importance of orthogonal methodologies (like MS), we have now added the following information:

1. Discussion (page 12, lines 22-26): "In this cross-sectional multicentric study, we used large-scale PEA proteomics to identify the minimal set of biomarkers for the differential diagnosis of AD, DLB, and FTD, as well as for the molecular staging of AD. We next translated findings into a custom 21-plex PEA which was validated in an independent validation cohort. Specific biomarker candidates for DLB were further validated in independent autopsy cohorts."
2. Limitations (page 14, lines 41-42): "v) The results obtained are to some extent platform dependent, and additional proteins might be also identified with other alternative and complementary approaches.

The authors exclude proteins with >15% below the LOD. However, it seems as though these proteins can still be examined – at least in supplementary exploratory analyses – as a binary indicator with values below LOD coded as 0 and values above LOD coded as 1. The 'absence' of some proteins is likely informative, and this approach would allow you to determine if this is the case.

We agree this is a stringent quality control step. Although values below the limit of detection (LOD) may still carry biological information, our primary objective was to identify robust and reliable biomarkers suitable for clinical translation. When a measurement falls below the LOD, it is impossible to determine whether it reflects a true low biological value or an assay-related issue. Moreover, repeated testing of the same sample could yield substantial variability within this range. For these reasons, applying a 15% threshold is a prudent choice to ensure robustness. Including proteins with

high proportions of undetectable values could hinder assay development and implementation, as recent experience with our CSF AD biomarker panels (Hok-a-Hin et al, AR&T, in press).

Nonetheless, in response to the reviewer's comment, we conducted a sensitivity analysis using NPX values (rather than binary coding) to evaluate the relevance of excluded proteins (those excluded for both LOD and QC warnings). Although there were proteins showing significant differences ($q < 0.05$), none demonstrated to be a stronger biomarkers candidate than those already included in the panel.

We provide below a list of the proteins excluded that showed significant differences across any of the comparisons, their AUCs, and relative rankings on average AUC to be compared to those in Tables S6–S8. To avoid confusion, the tables below have not been added to the manuscript. Instead, we have better clarified this in the method section (page 5, lines 22-24) as follows: "Sensitivity analysis showed that none of the excluded proteins offered better discriminatory value than those retained in the panel."

Excluded plasma proteins with significant differences across AD continuum. *Area under the ROC curve (AUC) is reported for each of the excluded proteins and each comparison, for proteins producing an adjusted p-value (q-value) below 0.05 in alla the five comparisons. Average AUC and its rank among all the tested proteins are also reported.*

protein	↑/↓ in AD	preAD vs CTRL	MCI-AD vs CTRL	AD-dem vs CTRL	AD-dem vs FTD	AD-dem vs DLB	avg	rank
CNST	0	0.57	0.62	0.71	0.7	0.71	0.68	14
DOK2	0	0.6	0.64	0.74	0.69	0.69	0.68	17
CD69	0	0.59	0.63	0.73	0.68	0.71	0.68	21
CEP85	0	0.6	0.68	0.76	0.67	0.69	0.68	22
SESTD1	0	0.59	0.65	0.77	0.68	0.68	0.68	26
DNMBP	0	0.6	0.69	0.76	0.64	0.7	0.67	32
PRKAR1A	0	0.59	0.63	0.74	0.66	0.69	0.67	37
WASF1	0	0.58	0.64	0.73	0.67	0.68	0.67	38
STX6	0	0.59	0.69	0.76	0.63	0.69	0.67	40
MITD1	0	0.59	0.63	0.72	0.68	0.67	0.66	46
MYO9B	0	0.59	0.73	0.76	0.61	0.68	0.66	47
HSPB1	0	0.6	0.65	0.7	0.67	0.67	0.66	51
PTPN1	0	0.59	0.67	0.74	0.64	0.67	0.66	52
CC2D1A	0	0.59	0.67	0.75	0.64	0.66	0.66	56
SNAP23	0	0.58	0.6	0.69	0.68	0.67	0.66	63
TST	0	0.59	0.58	0.71	0.66	0.67	0.65	73
SMAD1	0	0.58	0.61	0.72	0.67	0.65	0.65	75
CA13	0	0.59	0.61	0.7	0.66	0.66	0.65	76
STX16	0	0.58	0.69	0.75	0.63	0.65	0.65	77
SERPINB9	0	0.59	0.63	0.68	0.66	0.66	0.65	79
DIABLO	0	0.59	0.63	0.71	0.65	0.66	0.65	82
MZT1	0	0.58	0.69	0.77	0.61	0.66	0.65	83
LACTB2	0	0.59	0.62	0.7	0.66	0.65	0.65	90
PLXNB3	0	0.59	0.64	0.7	0.65	0.65	0.65	93
FGF2	0	0.57	0.61	0.68	0.66	0.66	0.65	98

MAP4K5	0	0.58	0.58	0.68	0.67	0.65	0.65	99
RHOC	0	0.58	0.6	0.69	0.65	0.66	0.65	100
AKT3	0	0.57	0.6	0.71	0.65	0.65	0.64	102
MCFD2	0	0.58	0.6	0.69	0.65	0.65	0.64	117
EBAG9	0	0.58	0.6	0.68	0.64	0.65	0.64	121
CLPP	0	0.58	0.6	0.67	0.64	0.65	0.64	125
ICA1	0	0.59	0.62	0.7	0.64	0.64	0.64	129
TBCB	0	0.59	0.59	0.67	0.66	0.64	0.64	130
TXLNA	0	0.59	0.58	0.66	0.67	0.64	0.64	132
SNX9	0	0.58	0.59	0.68	0.65	0.64	0.64	133
PRKAB1	0	0.57	0.65	0.73	0.61	0.65	0.64	135
CRADD	0	0.59	0.61	0.68	0.65	0.64	0.64	136
CD63	0	0.57	0.63	0.67	0.64	0.65	0.64	138
TBCC	0	0.59	0.6	0.67	0.65	0.64	0.64	139
VSIR	0	0.58	0.62	0.7	0.63	0.64	0.64	140
FKBP5	0	0.59	0.61	0.68	0.63	0.64	0.63	143
PPP1R2	0	0.58	0.58	0.66	0.65	0.64	0.63	144
PRDX3	0	0.57	0.61	0.67	0.64	0.64	0.63	145
DARS1	0	0.59	0.6	0.68	0.63	0.65	0.63	149
WWP2	0	0.6	0.65	0.72	0.61	0.63	0.63	155
STK4	0	0.58	0.59	0.66	0.64	0.64	0.63	159
SRPK2	0	0.57	0.61	0.69	0.63	0.64	0.63	160
GRAP2	0	0.59	0.57	0.65	0.67	0.63	0.63	162
APRT	0	0.59	0.6	0.67	0.62	0.65	0.63	166
FADD	0	0.59	0.6	0.67	0.64	0.63	0.63	167
TYMP	0	0.58	0.61	0.69	0.63	0.63	0.63	169
PLPBP	0	0.59	0.6	0.67	0.64	0.62	0.63	170
CETN2	0	0.6	0.61	0.68	0.62	0.64	0.63	171
STAMBP	0	0.58	0.59	0.67	0.64	0.63	0.63	181
CEP20	0	0.59	0.59	0.66	0.64	0.62	0.63	182
ACY1	0	0.58	0.64	0.68	0.69	0.55	0.63	188
CHMP1A	0	0.58	0.56	0.63	0.65	0.63	0.63	189
PLIN3	0	0.57	0.63	0.69	0.61	0.64	0.62	191
MAX	0	0.59	0.6	0.66	0.63	0.63	0.62	193
FXYD5	0	0.59	0.58	0.66	0.65	0.61	0.62	194
STK24	0	0.59	0.59	0.7	0.62	0.62	0.62	200
ACAA1	0	0.59	0.62	0.68	0.63	0.61	0.62	202
GNE	0	0.58	0.6	0.69	0.62	0.62	0.62	203
FKBP1B	0	0.59	0.57	0.67	0.62	0.63	0.62	205
ANXA4	0	0.58	0.6	0.66	0.62	0.63	0.62	206
KYAT1	0	0.58	0.6	0.67	0.63	0.62	0.62	211
AHCY	0	0.6	0.6	0.65	0.62	0.63	0.62	212
DNAJB8	0	0.58	0.61	0.7	0.61	0.62	0.62	214
IMPA1	0	0.58	0.6	0.67	0.62	0.62	0.62	215
QDPR	0	0.59	0.61	0.67	0.64	0.6	0.62	216
BIRC2	0	0.58	0.58	0.68	0.62	0.63	0.62	220
CERT	0	0.6	0.6	0.68	0.62	0.61	0.62	221
SCLY	0	0.58	0.63	0.68	0.67	0.55	0.62	225
ATXN10	0	0.58	0.58	0.66	0.62	0.63	0.62	228

GUSB	0	0.57	0.63	0.67	0.66	0.57	0.62	230
CTF1	0	0.57	0.59	0.67	0.62	0.62	0.62	231
CD2AP	0	0.58	0.57	0.65	0.64	0.6	0.61	241
EIF4B	0	0.58	0.56	0.63	0.64	0.61	0.61	244
FKBP4	0	0.58	0.56	0.63	0.63	0.62	0.61	246
CDC27	0	0.58	0.6	0.69	0.6	0.61	0.61	247
TPP1	0	0.59	0.58	0.61	0.64	0.6	0.61	248
HNRNPK	0	0.59	0.56	0.65	0.62	0.61	0.61	255
CASP10	0	0.58	0.6	0.68	0.61	0.61	0.61	257
SLC27A4	0	0.61	0.6	0.69	0.61	0.59	0.61	260
PSRC1	0	0.58	0.62	0.68	0.62	0.58	0.61	262
MAPK9	0	0.56	0.59	0.67	0.61	0.61	0.61	263
RAD23B	0	0.58	0.55	0.65	0.61	0.61	0.61	264
LGMN	0	0.58	0.6	0.66	0.63	0.57	0.61	265
PSME1	0	0.58	0.59	0.64	0.61	0.61	0.61	266
BAIAP2	0	0.57	0.62	0.64	0.61	0.6	0.61	267
NUDT2	0	0.57	0.59	0.66	0.59	0.62	0.61	270
YTHDF3	0	0.56	0.57	0.63	0.63	0.6	0.6	277
NFKBIE	0	0.56	0.56	0.65	0.62	0.6	0.6	280
SELP	0	0.57	0.62	0.68	0.62	0.56	0.6	281
DPP7	0	0.58	0.58	0.62	0.62	0.58	0.6	284
CXCL5	0	0.58	0.58	0.66	0.61	0.57	0.6	289
SCRN1	0	0.57	0.58	0.63	0.58	0.61	0.6	292
ABHD14B	0	0.58	0.59	0.63	0.6	0.59	0.59	295
CCT5	0	0.58	0.59	0.64	0.58	0.59	0.59	298
EIF4EBP1	0	0.57	0.56	0.61	0.6	0.59	0.59	301
RRM2	0	0.58	0.6	0.65	0.62	0.54	0.59	302
SUMF2	0	0.56	0.58	0.64	0.62	0.55	0.59	306
creg1	0	0.57	0.58	0.58	0.61	0.56	0.58	308
VEGFA	0	0.6	0.58	0.61	0.6	0.54	0.58	309
NPY	0	0.58	0.57	0.62	0.62	0.52	0.58	310
PSME2	0	0.57	0.57	0.58	0.57	0.59	0.58	311
TNFSF14	0	0.56	0.6	0.62	0.59	0.55	0.58	312
CCS	0	0.59	0.58	0.61	0.57	0.55	0.57	315
DKK1	0	0.58	0.58	0.59	0.58	0.54	0.57	317
CCN1	0	0.56	0.56	0.65	0.59	0.51	0.56	318
AK1	0	0.57	0.56	0.58	0.57	0.55	0.56	319
TIMP1	0	0.53	0.54	0.6	0.55	0.57	0.56	320
SERPINE1	0	0.58	0.62	0.63	0.57	0.5	0.56	321
SDC4	0	0.59	0.57	0.57	0.57	0.53	0.56	322
SORT1	0	0.55	0.59	0.62	0.57	0.52	0.56	323
SPARC	0	0.58	0.57	0.56	0.58	0.51	0.55	324
FUT8	0	0.57	0.59	0.6	0.55	0.51	0.55	325
PDGFA	0	0.58	0.57	0.58	0.57	0.5	0.55	326
VEGFC	0	0.58	0.57	0.58	0.55	0.51	0.54	327
ANGPT1	0	0.57	0.57	0.55	0.55	0.51	0.54	328

Excluded plasma proteins with significant differences in DLB. *Area under the ROC curve (AUC) is reported for each of the excluded proteins and each comparison, for proteins producing an adjusted p-value (q-value) below 0.05 in the two comparisons. Average AUC and its rank among all the tested proteins are also reported.*

protein	↑/↓ in DLB	DLB vs CTRL	DLB vs AD-dem	avg	rank
ADGRG2	↓	0.68	0.657263	0.67	7
LEPR	↓	0.64	0.635449	0.64	11
GH1	↓	0.62	0.602772	0.61	14
SNCG	↑	0.55	0.560684	0.56	23

Excluded plasma proteins with significant differences in FTD. *Area under the ROC curve (AUC) is reported for each of the excluded proteins and each comparison, for proteins producing an adjusted p-value (q-value) below 0.05 in the two comparisons. Average AUC and its rank among all the tested proteins are also reported.*

protein	↑/↓ in FTD	FTD vs CTRL	FTD vs AD-dem	avg	rank
GH1	↓	0.65	0.633812	0.64	9
ADGRG2	↓	0.65	0.623874	0.64	10
CLEC4A	↓	0.60	0.607099	0.60	20
APLP1	↓	0.61	0.598808	0.60	21
BMP4	↓	0.58	0.590745	0.59	24
PLIN1	↑	0.53	0.577826	0.56	27

Please specify how you handled missing values below LOD for proteins with 0 to 15% of values below LOD. Did you impute values < LOD? Or did you use the provided <LOD values for analyses? Or did you simply exclude the measurement. The latter option could result in some bias, so consider imputing the < LOD results as part of a sensitivity analysis.

As recommended by the PEA platform provider (https://olink.com/knowledge/faq?query=lod) and in line with published literature (Bai et al., 2025; Eldjarn et al., 2023), we did not impute or exclude values below the limit of detection (LOD). Instead, we used the provided NPX values directly in all analyses, including those below the LOD. These values represent actual quantification estimates and are not considered missing data. Considering our stringent quality control criteria, only 0.74% data points had values below LOD. This limited % of data point is unlikely to cause any potential bias, and thus no imputation methods is required. In addition, retaining below-LOD values improves data distribution and statistical power and may reveal meaningful differences between groups. This approach is widely accepted in the field and avoids introducing bias through artificial dichotomization or exclusion. Accordingly, we added in the methods section (page 5, lines 20-22):

"For included proteins, NPX values below the LLOD were not imputed or substituted, as they represent only 0.74% of the total data and we considered the measured values to represent the best available estimates"

The authors state “proteins with more than 15% values associated with quality control (QC) warnings and then samples producing more than 35% QC warnings were removed from subsequent analyses.” It’s not exactly clear what the difference is between this 15% and this 35% and how QC warnings were determined. Please elaborate on this so that the reader can understand the rationale for QC exclusions.

We thank the reviewer for raising this important point. All pre-cleaning steps in the discovery cohort were performed in accordance with previous work and supported by Olink’s recommended guidelines to ensure the robustness, reproducibility, and translatability of the assay, which is particularly important for downstream development in clinical or high-throughput settings. QC warnings are automatically assigned by the Olink system when specific internal or external control criteria are not met. Specifically, a sample receives a QC warning if:

- The average matched counts per sample fall below 500,
- The incubation or amplification controls deviate more than ±0.3 NPX from the plate median,
- The negative control for an assay deviates more than 5 standard deviations from its predefined target.

To clarify our thresholds:

The 15% threshold was applied at the protein level: proteins with more than 15% of NPX values flagged with QC warnings were excluded to ensure data reliability and to prioritize proteins with good assay performance across samples.

The 35% threshold was applied at the sample level: samples with more than 35% of protein measurements flagged were excluded, as this typically indicates broader technical issues or suboptimal sample quality. This threshold was selected to balance data integrity with retention of valuable data, resulting in the exclusion of only 41 samples (~3% of the dataset). Notably, most samples showed less than 5% QC warnings (see histogram below).

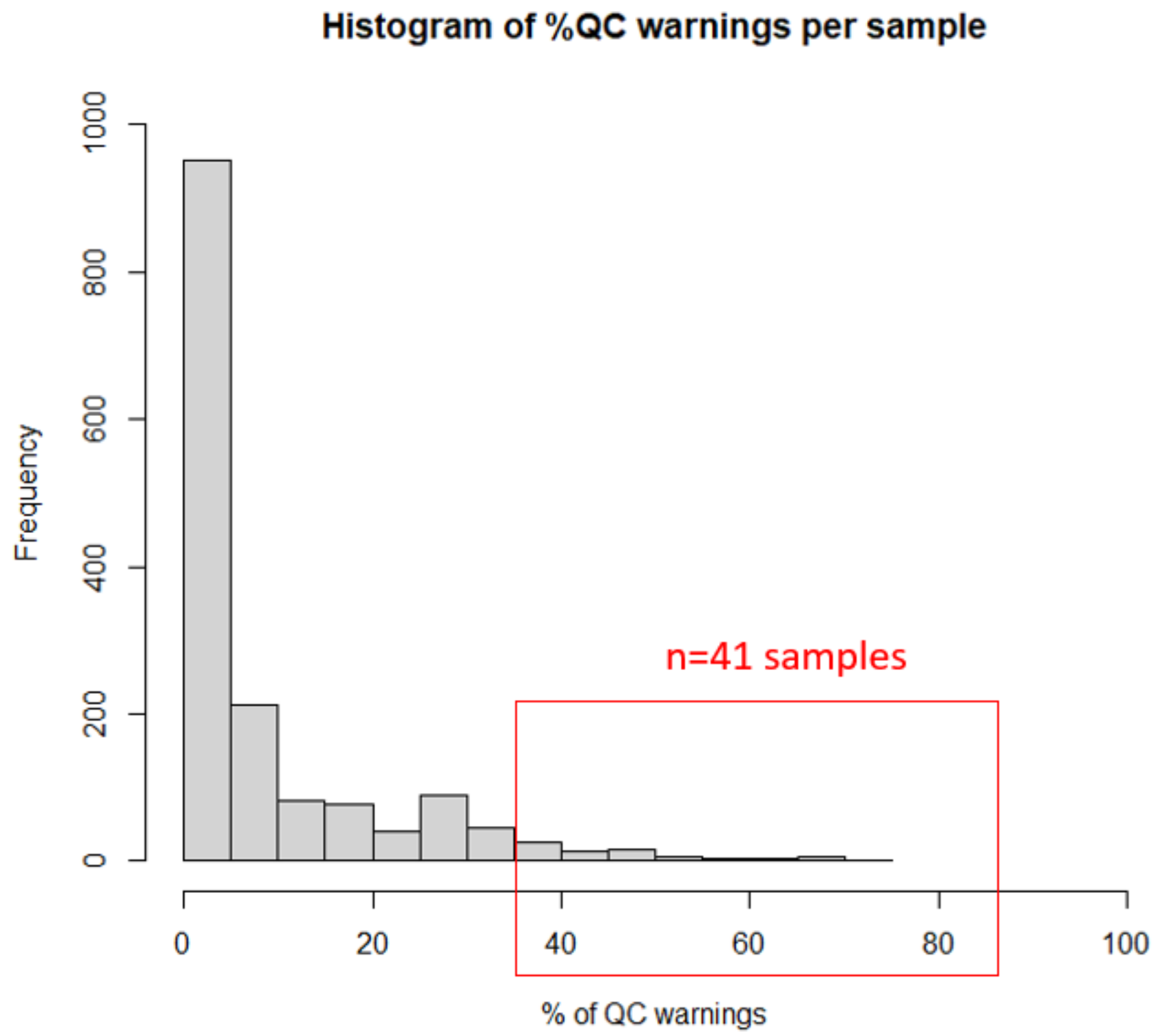

Similar procedures were applied in the validation cohort for absolute quantification values.

We have now clarified this is in the method section (page 5) as follows: “As quality control measures at the individual protein level, we excluded from sample analysis protein duplicates, those proteins with more than 15% values below the lower limit of detection (LLOD) and those proteins with more than 15% values associated with quality control (QC) warnings. As quality control measure for the sample level, samples producing more than 35% QC warnings were removed from subsequent analyses, as this typically indicates broader technical issues or suboptimal sample quality. For included proteins, NPX values below the LLOD were not imputed or substituted, as they represent only 0.74% of the total data and we considered the measured values to represent the best available estimates. Following these procedures, 520 proteins and 41 samples were removed (7 AD-dem, 12 CTRL, 4 DLB, 7 FTD, 6 MCI-AD, 1 MCI-FTD, 4 preAD), resulting in 1016 unique proteins measured in 1277 subjects. Sensitivity analysis showed that none of the excluded proteins offered better discriminatory value than those retained in the panel.”

“The same QC measures described above for the discovery cohort were applied within the validation using the custom PEA assay. A total of 83 samples (n=13 CTRL, n=16 preAD, n=5 MCI-AD, n=16 AD-dem, n=6 MCI-DLB, n=2 MCI-FTD, n=15 DLB, n=9 FTD) were not included within the following statistical analysis due to QC warning in more than 19 out of the 21 proteins measured within the panel (n=62), due to sample QC warnings (n=20) or both (n=1).”

Overall, and study/site-specific coefficient of variation (CV) information is needed. Were blind duplicates (technical replicates) examined? Blind duplicate intra- and inter-assay CV information for specific proteins should be used to provide the reader with information about reliability of measurements. This information is especially important for the candidate proteins.

Please note that all samples were run at Olink facilities at the same time and randomized across different plates. The diagnoses and centers were equally distributed across plates, please see the original schemes below:

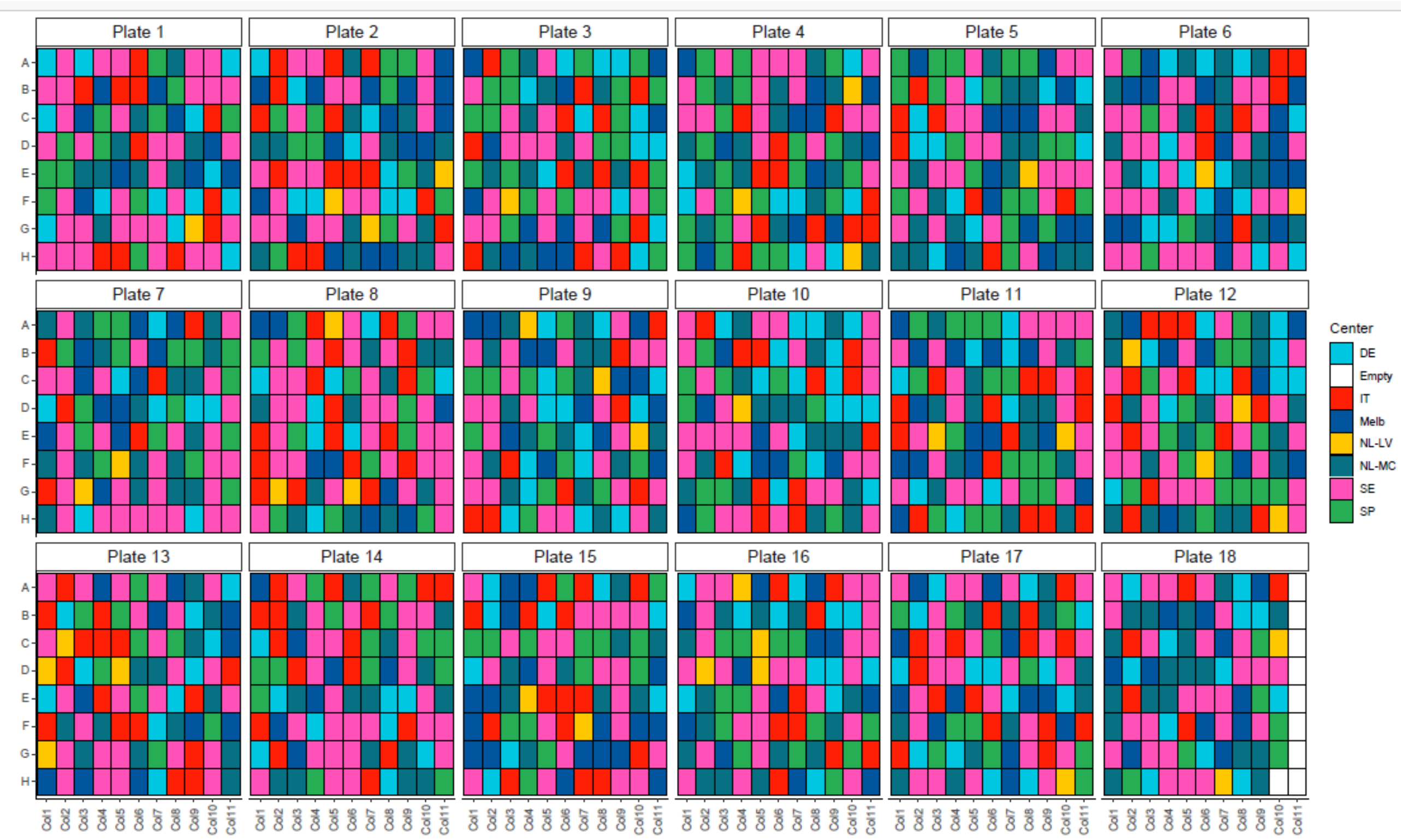

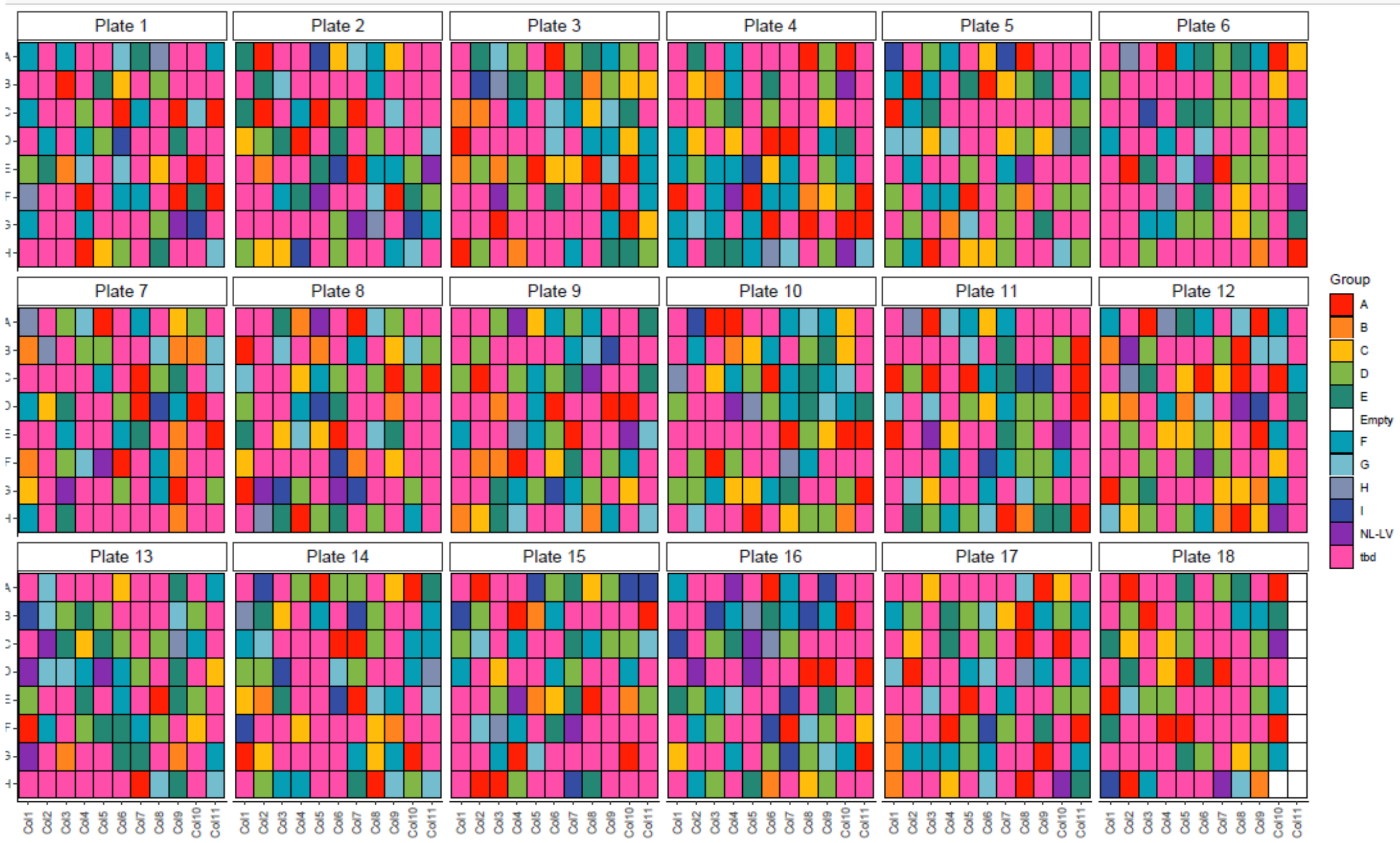

In the discovery phase, protein-specific CVs were calculated using both control samples (2 samples per plate) and plate controls (3 samples per plate). Four different types of CVs were calculated for each protein: Inter- and intra-plate CVs using control samples and inter and intra- plate CVs using plate controls. The averages of these CVs are shown below, with all mean CVs being <20%, a threshold optimal for performing measurement using targeted immunoassays. This information is now included as supplementary Table S4 and clarified in the methods section (page 5, Lines 8-13) as follows:

"Briefly, samples were randomized across 18 plates containing appropriate intra- and inter-plate quality controls (QC) from the manufacturer. Intra- and inter-plate coefficients of variation (CVs) for all panels are reported in Table S4, with average CVs being below 20%."

**Mean CV intra-plate control (%)**					
**Panel**	**plate A**	**plate B**	**plate C**	**plate D**	**Average**
Cardiometabolic	14.34	18.31	5.75	14.66	13.26
Inflammation	17.00	16.32	5.58	12.77	12.92
Neurology	20.50	14.93	8.39	11.06	13.72
Oncology	16.94	15.06	6.50	12.06	12.64
**Mean CV inter-plate control (%)**					
**Panel**	**plate A**	**plate B**	**plate C**	**plate D**	**Average**
Cardiometabolic	20.37	23.77	10.06	15.88	17.52
Inflammation	21.74	20.60	10.75	16.45	17.39
Neurology	25.64	19.28	17.34	17.57	19.96
Oncology	23.34	21.74	12.73	17.83	18.91

Table S4. Protein-specific intra- and inter-plate coefficients of variation (CVs) across PEA panels. Four types of CVs were calculated for each protein using both internal control samples (2 per plate) and plate controls (3 per plate): intra-plate CVs and inter-plate CVs for both control types. The table reports average CVs by panel across four assay plates (A–D). All mean CVs were below 20%, supporting the technical reliability of the measurements performed in the discovery cohort.

In the validation phase, a custom PEA panel, enabling absolute quantification of the 21 selected proteins, was manufactured and tested. The custom multiplex assay also showed fair CVs (intra- and inter- overall CVs <20% for most of the proteins. This information is now included as supplementary Table S5 and clarified in the methods section (page 5, lines 13-34) as follows:

"A custom-designed multiplex-PEA was developed by the manufacturer, following standardized protocols[34,35], to quantify the 21 proteins selected in the discovery cohort. The custom assay was then tested in an independent validation cohort (Table 1). Besides clinical samples, each plate included 3 replicates of four plasma QC samples (2 plasma pools made from AD patients and 2 plasma pools made from CTRL subjects) and three calibrators used for normalization. QC samples and calibrators were measured in triplicate. Each custom assay has an experimentally- determined LOD defined as for the discovery panels. Precision (intra- and inter-assay CV) were calculated using the 4 QC samples. No cross-reactivity between assays for specific proteins was detected. Assay parameters including LOD and CVs are included in Table S5."

Assay	CV intra-plate (%)	CV inter-run (%)	CV Intra-run inter-plate (%)	CV overall (%)
B4GAT1	5.9	6.7	10.2	11.9
CALCOCO1	5.9	11.8	9.6	15.0

CD38	5.4	12.7	6.4	14.3
CDKN1A	11.3	12.6	7.6	17.0
EGFR	7.0	13.8	6.6	15.5
ERBB3	5.9	13.0	5.7	14.8
FLT3LG	6.0	13.3	6.7	15.3
GFAP	8.4	25.2	10.3	25.8
IRAG2	6.9	8.7	7.3	12.4
ITGAM	5.4	10.6	4.2	12.2
ITGAV	4.4	8.3	7.2	11.1
MAP3K5	18.3	19.3	16.2	26.8
NCAN	5.5	11.6	4.6	13.4
NEFL	16.8	19.6	15.3	27.2
NTRK3	7.0	14.9	6.7	16.8
OSM	6.4	12.7	6.7	14.8
PECAM1	6.3	14.4	7.2	16.0
PI3	6.5	17.1	6.6	18.7
RET	5.9	13.3	5.8	14.9
TNFSF10	4.9	10.8	5.8	12.6
TRAF2	5.5	11.2	6.2	13.2

Table S5. Mean assay-specific coefficients of variation (CVs) for the custom panel, summarizing precision across runs and plates. For each analyte, we calculated four CVs, intra-replicate, inter-plate (within run), inter-run, and overall, based on absolute quantification values obtained from 3 technical replicates × 4 internal control plasma pools included on every plate in each run. Reported CVs are means across the four control pools. All mean CVs were <30% with the great majority being<20%, indicating robust technical performance of the custom PEA panel applied for validation.

The authors validated their assay on an internal, but independent, test set. However, the sizable effect of cohort-specific factors still leaves a major question about the generalizability of these results in a separate cohort. Specifically, what is needed is validation in a clinical or community-based cohort wherein participants are not include based on their AD, DLB, FTD diagnosis. This would provide a better sense of the assay's generalizability beyond the (impressive) multi-study cohort that you all have established.

In line with reviewers´ comment (both 1 and 2), we have now developed a multiplex custom assay for the absolute quantification of the 21 markers of interest and validated it in a completely independent cohort of 805 subjects (722 after precleaning). As can be seen from the newly added Figure 7, Figure S9, Figure S10, and from the modified Figure 6, Figure 8 and Figure S7, similar differences across groups and diagnostic performances have been obtained within this large, well-characterized independent validation cohort using a toolkit that can be used in the future by other research centers and clinical centers. Such data confirm our previous findings and the potential of this new tool to support the biological diagnosis of the main types of neurodegenerative diseases in plasma. We agree that the implementation of these biomarkers in clinical settings will require follow-up studies in community-based prospective cohorts, as currently being performed for the well-established AD plasma biomarkers (e.g., pTau217).

The authors need to discuss their use of covariates. I know this is less important when the goal is

discriminating one group from another, but there may need to be some consideration or at least discussion of the role of confounding factors. Is there a need to adjust for kidney function, BMI, age, or other systemic/demographic factors known to have an outsized effect on the proteome independent of disease/disease stage.

We thank the reviewer for this thoughtful comment. We agree that systemic and demographic factors can influence plasma protein levels and may act as confounders in biomarker discovery. Age and sex were included as covariates in all discovery univariate analyses, and all reported q-values are adjusted accordingly, as described in the "Statistical analysis" section of the manuscript.

As reviewer suggested, we also evaluated the potential impact of systemic comorbidities on the proteins included in the 21-biomarker panel. Anamnestic data were available for most participants and allowed us to classify comorbidities into six categories: dyslipidemia, hypertension, chronic kidney disease (CKD), obesity, hypothyroidism, and diabetes. For each comorbidity and each protein in the panel, we applied a nonparametric two-way ANOVA, using comorbidity status (present/absent) and clinical group as independent variables.

This analysis revealed that, in most cases, the clinical group had a substantially stronger effect than comorbidity status. Only three proteins showed a significant association where the comorbidity effect was comparable to or exceeded that of the clinical group: NTRK3 with obesity, and PECAM1 and IRAG2 with hypertension. Such comorbidities are not expected to change the results obtained as those proteins did not play a major effect on panel´s diagnostic performance. We believe this analysis reinforces the robustness of our biomarker findings and suggests that disease-related biological signals, rather than comorbid conditions, are the main drivers of the observed protein changes. These findings are now summarized in the newly added Supplementary Figure S8 and reported in the results in the revised manuscript (page 10, lines 33-40):

"We next evaluated the impact of relevant co-morbidities (dyslipidemia, diabetes, hypertension, hypothyroidism, chronic kidney disease) on the levels of the selected proteins, as they can influence the interpretation of blood-based biomarkers [45–47]. The selected proteins were largely unaffected by systemic comorbidities, with clinical group explaining most of the variance in protein levels. Only three proteins, NTRK3 (associated with obesity), and PECAM1 and IRAG2 (both associated with hypertension), showed significant comorbidity effects which were comparable or exceeded those produced by disease group (Figure S9). These proteins were not among the main contributors driving the discrimination across the different dementia types."

From the AD biomarker results, the 99% down-regulated finding is surprising. Is this consistent with other studies? Is it realistic to think, for example, that all pathways are downregulated in preAD, MCI-AD, and AD-dementia, as is found in the pathway analyses?

This is indeed an intriguing finding observed also in other plasma proteomic studies (Ali et al., 2025; Dammer et al., 2022; Heo et al., 2025; Jiang et al., 2022). In line with our results, the recently published Atlas of the plasma proteome also showed that diseases of the nervous system, including Alzheimer's disease, Vascular dementia, or Parkinson´s disease were grouped together in clusters in which most biological pathways (including autophagy as also observed in this study) were downregulated (Deng et al., 2025). We speculate that this generalized pathway outage might be a systemic respond of the body to the stress produced by the disease process and be able to redirect resources to very specific sites/organs. It might be also the result of alterations in protein metabolism or other systemic physiological disturbances (hydration levels, kidney function) associated with

Alzheimer's disease. Further research is thus needed to understand the biological reason behind the plasma proteome downregulation. This has now been included in the discussion (page 12, lines 28-40) as follows:

"Unlike observed in previous CSF proteomics studies[52], most AD-associated plasma proteins were decreased compared to CTRL, DLB, and FTD. This widespread downregulation observed in plasma has also been reported in studies using SomaScan, mass spectrometry, and in the recent plasma proteome Atlas[53–57]. This generalized reduction may reflect a systemic response to neurodegenerative stress, potentially redirecting resources to affected organ. It may also result from alterations in protein metabolism or other physiological disturbances in AD. Of note, CALCOCO1, BIN2, and MAP3K5 were among the strongest downregulated proteins specifically associated with AD. CALCOCO1, previously reported as downregulated in AD plasma proteomics[58], and MAP3K5, which progressively declines across the AD continuum, both regulate ER-mediated autophagy[59,60], a process associated to AD pathophysiology[61,62]. Interestingly, BIN2, is involved in phagocytosis, and has been identified as a microglial Aβ response-associated protein in transgenic murine models of amyloidosis[63]. These findings are in line with the results of the pathway enrichment analysis, which pinpointed to mechanisms primarily related to dysfunctional autophagy in the three AD stages considered. "

Reviewer #3 (Remarks to the Author):

The multicenter study of Bellomo et al. is an excellent and timely manuscript that addresses a highly relevant topic in the field of neurodegenerative diseases. The study is both comprehensive and methodologically robust, providing powerful and novel insights into the plasma proteomic signatures that distinguish Alzheimer's disease (AD), dementia with Lewy bodies (DLB), and frontotemporal dementia (FTD). The authors' use of large, well-characterized multicenter cohorts and advanced proteomic profiling represents a significant step forward for the field. The authors (i) identified GFAP as a robust marker for the AD continuum, with levels increasing from preclinical to dementia stages; (ii) discovered that integrins ITGAV and ITGAM are strongly downregulated in DLB and correlate with α-synuclein pathology in autopsy-confirmed cases; (iii) showed that elevated NEFL and reduced GFAP levels are the strongest markers specifically associated with FTD. Finally, the authors developed a 21-protein panel that achieves high diagnostic accuracy (AUC >0.87) for differential diagnosis and staging across dementia types. The methodology is rigorous, with appropriate quality control, statistical analysis, and validation in both autopsy-confirmed and independent cohorts. The statistical approach is sound, with logistic regression models adjusted for age and sex, multiple testing correction, and clear reporting of effect sizes and AUCs, and the authors are transparent about limitations such as the small MCI-DLB subgroup and potential center effects. The conclusions are robust and reliable, supported by the large sample size, multicenter validation, and biological plausibility of the identified markers, though the manuscript could be improved by more thoroughly discussing the potential influence of comorbidities (e.g., vascular or metabolic conditions), expanding on the mechanistic implications of the findings, and including additional external validation in more diverse populations or longitudinal analyses. References are generally appropriate and credit key prior work but could be strengthened by citing recent large-scale proteomic studies for context. I greatly appreciated reading the manuscript and have some revisions that the authors may wish to consider addressing.

1. Given that GFAP is elevated in AD but reduced in FTD, the authors may wish to discuss whether this disparity reflects divergent astrocytic responses or underlying disease-specific mechanisms (e.g., tau versus TDP-43 pathology).

We thank the reviewer for this thoughtful suggestion. We agree that differences in GFAP levels between AD and FTD may reflect distinct underlying pathophysiological mechanisms. Please note that, similar to other studies (Zhu et al., 2021), we observed higher levels of GFAP in FTD, although to a lesser extent than in AD. We have now further discussed this topic, including the role of GFAP across FTLD subtypes, as follows (page 14, lines 12-27)

"In the case of FTD, we identified 23 proteins that were dysregulated compared to both AD-dem and CTRL in the discovery cohort. NEFL showed the greatest effect size compared to CTRL, while GFAP had the most significant effect in comparison with AD-dem. LASSO routine identified a signature with 10 and 2 proteins that could well discriminate FTD from CTRL and AD-dem respectively, in both discovery and independent validation cohorts (Figure 6). The strongest contributors to these two models were GFAP and NFL. Considering that NEFL is not a FTD-specific biomarker and has limited performance to discriminate FTD from AD-dem[83–85], GFAP is likely the biomarker driving the strongest effect to discriminate these groups. In line with previous studies, we observed that GFAP was elevated in FTD compared to controls, but to a considerable lesser extent than in AD[86], suggesting a different or reduced astrocytic response in FTD compared to AD. It should be noted that other studies have shown increased levels of plasma GFAP only in FTD cases with GRN mutation[87]. Differences on plasma GFAP levels have also been recently observed across FTD pathological subtypes[88], further indicating that the levels of plasma GFAP vary across genetic and pathological FTD subtypes. We also observed that OSM and MMP9 were increased in MCI-FTD compared with both CTRL and MCI-AD, the former further validated in the validation cohort. Although MMP9 could not be included in the custom assay due to technical incompatibilities, similar changes were also observed in our recent CSF proteomic study of FTD[82]. OSM and MMP9, which have been previously associated to inflammatory response[89] and brain-blood barrier disruption[90,91], respectively, are promising plasma biomarker candidates specifically associated to FTD that may probe earlier pathogenic events in FTD and NEFL changes. Future studies are needed to understand the expression of these proteins along the complex spectrum of FTD subtypes."

2. The finding that ITGAV and ITGAM are decreased in DLB, with validation across cohorts including one with autopsy confirmation, is a notable strength. However, the biological interpretation of integrin downregulation remains speculative. The discussion touches briefly on their role in adhesion and signaling, but does not provide a mechanistic hypothesis for their involvement in α-synucleinopathies or explain how such downregulation would translate to systemic circulation. The authors may wish to provide a better biological contextualization

We thank the reviewer for this thoughtful comment. We agree that the mechanistic interpretation of integrin downregulation in α-synucleinopathies merits further elaboration. Accordingly, we have revised the Discussion to better contextualize the biological relevance of ITGAV, ITGAM, and ERBB3 in the setting of Lewy body disorders (LBD), emphasizing their roles in immune regulation, synaptic function, and neurotrophic signaling through the NRG1–ERBB3–integrin axis. We have also incorporated findings from a recent large-scale proteomic study (Gan et al., Nat Aging, 2025) that identified these same proteins as altered in plasma samples of individuals with Parkinson's disease in the UK Biobank cohort using the PEA platform, further supporting their potential relevance to α-synuclein-related pathology.

Revised discussion (page 13, lines 29-44):

"Integrins mediate cell-to-cell and cell-to-matrix interactions, with known roles in immune regulation, synaptic remodeling, and blood–brain barrier integrity. However, their involvement in neurodegenerative diseases remains largely unknown[76]. Of note, ITGAV forms a functional signaling complex with ERBB3[77]. This integrin–ERBB3 complex is activated by neuregulin-1 (NRG1), a trophic

factor essential for neuronal survival, axonal guidance, and synaptic plasticity. Notably, NRG1 levels have previously been reported to be reduced in Parkinson's disease[78] and has been investigated as a pharmacological target for restoring dopaminergic function[79]. The coordinated downregulation of ITGAV and ERBB3 may therefore reflect impaired NRG1-mediated neurotrophic signaling in LBD, potentially contributing to synaptic dysfunction or reduced neuronal resilience. In line with this, ITGAV has been shown to mediate dopaminergic neurite outgrowth and connectivity in experimental model[80], suggesting that integrin dysregulation could represent a downstream consequence of dopaminergic pathway impairment, a key dysfunctional pathway in DLB and PD. The modest reduction of these integrins in plasma from FTD patients further supports this hypothesis, in line with evidence of dopaminergic dysfunction in approximately one-third of FTD cases[81] and the CSF alterations in dopaminergic markers observed also in FTD.[82]"

3. The manuscript does not sufficiently address the potential role of comorbidities in explaining the observed proteomic signatures. While the authors carefully account for co-pathology (e.g., Aβ status in DLB cases), systemic comorbidities (e.g., cardiovascular/metabolic diseases) that could confound plasma protein levels are not discussed. The authors may wish to add a paragraph in the discussion on comorbid conditions as potential modifiers of the proteomic landscape.

In response to a similar comment raised by another reviewer, we performed an exploratory analysis to specifically assess the potential impact of common comorbidities on the proteins included in our final 21-biomarker panel. While age and sex were already included as covariates in all univariate analyses in the discovery cohort (as detailed in the "Statistical analysis" section), we further classified available anamnestic data into six major comorbidity categories: hypertension, dyslipidemia, diabetes, chronic kidney disease (CKD), obesity, and hypothyroidism. For each comorbidity and each protein, we applied a nonparametric two-way ANOVA with comorbidity status (present/absent) and clinical group as independent variables.

This analysis showed that, for nearly all proteins, the disease group explained substantially more variance in protein levels than comorbidity status. Only three proteins demonstrated comorbidity effects comparable to or stronger than those related to clinical diagnosis: NTRK3 (associated with obesity), and PECAM1 and IRAG2 (associated with hypertension). These results are now included in the revised manuscript (page 10, lines 33-40):

"We next evaluated the impact of relevant co-morbidities (dyslipidemia, diabetes, hypertension, hypothyroidism, chronic kidney disease) on the levels of the selected proteins, as they can influence the interpretation of blood-based biomarkers [45–47]. The selected proteins were largely unaffected by systemic comorbidities, with clinical group explaining most of the variance in protein levels. Only three proteins, NTRK3 (associated with obesity), and PECAM1 and IRAG2 (both associated with hypertension), showed significant comorbidity effects which were comparable or exceeded those produced by disease group (Figure S9). These proteins were not among the main contributors driving the discrimination across the different dementia types."

4. The discussion is dense and at times repetitive, especially where findings are reiterated without adding new interpretation (e.g. GFAP is discussed at length in multiple places; the integrin findings are repeated in both the DLB and general conclusion sections).

We thank the reviewer for her/his comment, we have now revised the discussion to avoid repetitions and redundancies.

5. Multiple testing correction: could the authors clarify whether Benjamini-Hochberg was applied to all 1016 proteins or per panel.

We thank the reviewer for the comment. Benjamini–Hochberg correction was applied across all 1016 proteins, as appropriate for the full dataset. In the VARS and IBB cohorts, only 21 proteins were available, so the appropriate correction was applied within that subset. This is indicated in the methods section as follows (page 6, lines 6-10): **"**Logistic regression (LR) was applied to analyze the differential protein expression for the pairwise comparison of clinical groups using z-scored NPX or absolute quantification data, adding age and/or sex as covariates and adjusting p-values for multiple testing with Benjamini and Hochberg correction[36]. Adjusted p-values were deemed to as q-values.**"**

6. The authors should verify the total number of subjects enrolled in the study. The sum of individuals reported in the Introduction is 1,318, whereas the manuscript consistently refers to 1,319 participants. This discrepancy appears to concern the AD cohort: the total number reported in the Introduction (n=532) does not match the sum of the AD subgroups listed in the Methods section (preAD n=187, MCI-AD n=164, AD-dem n=182; total n=533).

We apologize to the reviewer and thank her/him for noticing this inconsistency. Indeed, it is an error in the description of the original preAD patients during manuscript drafting. The error was just present in the manuscript text and did not impact any of the analyses performed. The correct initial number of total subjects (n = 1318) and the number of preAD cases (n =186) have been changed accordingly.

7. Line 74-76: To maintain consistency with the VARS study, the authors may wish to place the acronym PPMI in parentheses before the sample size, as they did for VARS (e.g. …PEA data from the Parkinson's Progression Markers Initiative 9000 study (PPMI, n=221) was also used…)
8. Line 84: For consistency with the rest of the paragraph and because the acronym "Ulm" is used in the manuscript (figures and tables), the authors should include the acronym in parentheses after "Ulm University" (e.g., Ulm University (Ulm; Germany; n=174)), as done for the other cohorts.

We made all the requested changes.

9. I believe it would be helpful for readers—especially those less familiar with the field—if the Participants and Inclusion Criteria section of the Methods explicitly stated the criteria used to define a "positive AD biomarkers" when referring to CSF markers analysis, as opposed to PET imaging data.

According to reviewer´s comment we have explicitly included in the main text all the criteria used to define positive AD biomarkers, which are also included in supplementary table 1. Now the text reads as follows (page 3, lines 10-28):

"CSF markers were analyzed locally as part of the diagnostic procedure using commercially available kits (ADC, AIBL and Ulm University: ELISA INNOTEST, Fujirebio, Gent, Belgium; ADC: Elecsys biomarker assays[21], Roche Diagnostics GmbH, Basel, Switzerland; SPIN, UNIPG, and Ulm University: Lumipulse G600, Fujirebio[8,22]; BioFINDER: Euroimmun ELISA assays, Lübeck, Germany)[7,21]. All samples from AIBL cohort were defined based on Aβ and Tau PET[23,24]. Positive CSF AD biomarker profile was defined locally in the different cohorts as follows: in ADC as Aβ+ (Innotest Aβ42 < 813 pg/mL; Elecsys Aβ42 < 1000 pg/mL) and Tau+ (Innotest p-tau181 > 52 pg/mL; Elecsys p-tau181 > 24 pg/mL); in BioFINDER as Aβ42/Aβ40 < 0.088 and either p-tau181 > 65.04 pg/mL or t-tau > 462.91 pg/mL; in SPIN as Aβ42/Aβ40 < 0.062 and either t-tau > 456 pg/mL or p-tau181 > 63 pg/mL; in UNIPG as Aβ42/Aβ40 < 0.072 and p-tau181 > 50 pg/mL (regardless of t-tau); in UU as Aβ42 < 450 pg/mL and either t-tau > 380 pg/mL or p-tau > 65 pg/mL; in CBAS as either CSF biomarkers (Aβ42 < 526 pg/mL and p-tau181 > 50.2 pg/mL) or amyloid PET positivity; and in

AIBL based on amyloid and tau PET (amyloid PET positive > 25 Centiloids; tau PET positive meta-temporal standardized uptake value ratio > 1.19). The methodology and cutoff values used for each cohort and each biomarker are summarized in Table S1."

10. The authors should correct a-synuclein to α-synuclein (e.g. see line 119)

We made the corrections.

11. The authors should standardize the nomenclature: α-Syn or αSyn

We standardized the nomenclature to "αSyn" in the main text, captions and figures.

12. Line 175: The authors should replace Figure 5B with Figure 5C
13. Line 283: The authors should replace Figure 5C with 5B
14. Line 289, the authors should correct CA with CA1
15. Supplementary Table 6: The authors should adjust the number of FTD-specific plasma proteins: 19 should be replaced with 23.
16. Reference to Figure 7B is missing in the text
17. The legend of Figures (e.g. Figure 3) should be checked for grammar mistakes
18. Figure 6: The authors should verify the figure legend. The color labeling, as the training set appears to be shown in green rather than blue, and the test set in orange rather than red

We made all the required minor changes and corrections, and sincerely thank the Reviewer for her/his efforts to improve the manuscript.
